# International Cardiovascular Development, Anatomy, and Regeneration (ICDAR) Community Meeting: Prague 2024

**DOI:** 10.3390/jcdd11120390

**Published:** 2024-12-04

**Authors:** David Sedmera, Antonio Baldini, Maurice van den Hoff, Bill Chaudhry

**Affiliations:** 1 Institute of Anatomy, First Faculty of Medicine, Charles University, 128 00 Prague, Czech Republic; 2 Department of Molecular Medicine and Medical Biotechnology, School of Medicine, University Federico II, 80131 Naples, Italy; bldntn@gmail.com; 3 Department of Medical Biology, University Medical Center Amsterdam, University of Amsterdam, 1105AZ Amsterdam, The Netherlands; m.j.vandenhoff@amsterdamumc.nl; 4 Biosciences Institute, Newcastle University, Centre for Life, Central Parkway, Newcastle upon Tyne NE1 3BZ, UK; bill.chaudhry@newcastle.ac.uk

**Keywords:** heart development, cardiac progenitors, congenital heart disease, echocardiography, European Society of Cardiology

## Abstract

The International Cardiovascular Anatomy, Development, and Regeneration meeting was held from 18–20 September 2024, in Prague, Czech Republic, supported by the European Society of Cardiology’s Working Group on Development, Anatomy, and Pathology. Hosted at the Institute of Anatomy, First Faculty of Medicine, the event began with a hands-on workshop on normal and malformed human hearts, covering morphology, echocardiographic imaging, and rare congenital cases. The session allowed participants to examine and image both normal and malformed hearts. The main conference featured nine platform sessions on topics including pediatric cardiology, cardiac progenitors biology, congenital heart disease mechanisms, and cardiac regeneration. Highlights included two keynote lectures on cardiac genetics and development. In keeping with an established, decades-long tradition, the conference is a well-attended event, marking significant engagement in the latest cardiovascular research. The next appointment will be in Granada, Spain, 15–17 October 2025.

## 1. Introduction

The International Cardiovascular Anatomy, Developmental and Regeneration (ICDAR) meeting took place between 18 and 20 September 2024 in Prague, Czech Republic. This meeting is one in a long line of conferences that started in 1982 as the meeting of the European Society of Cardiology (ESC) basic science working group on Embryology and Teratology, aiming at understanding the morphogenetic basis of cardiac congenital diseases. With the start of the molecular era, the name changed to Working Group on Development, Anatomy and Pathology [1]. This current ICDAR meeting in Prague was endorsed by the Working Group Development, Anatomy and Pathology of the European Society of Cardiology and hosted at the Institute of Anatomy, First Faculty of Medicine, under the auspices of the Dean, Martin Vokurka. As is tradition, the meeting started with a “hands-on” workshop demonstrating normal and congenitally malformed human hearts. Normal anatomy and morphology of the human heart was summarized by David Sedmera (Prague, Czech Republic), and Viktor Tomek (Prague, Czech Republic) discussed the workflow of echocardiographic imaging of the pediatric patient. Subsequently, specimens of congenitally malformed hearts (including transposition of great arteries and different examples of Tetralogy of Fallot) were shown by Lucile Houyel (Paris, France) and Adrian Crucean (Birmingham, UK). Workshop participants were then allowed to examine specimens of normal and malformed hearts and perform echocardiography under the expert guidance of the course leaders.

The next two and a half days were devoted to cutting-edge oral presentations selected from the abstracts and organized into the following nine platform sessions: (1) pediatric cardiology and origins of congenital heart disease, (2) cardiac progenitors I, (3) cardiac regeneration, (4) cardiac valves, (5) mechanisms of congenital heart diseases, (6) cardiac progenitors II, (7) epigenetics, (8) cardiac conduction and arrhythmias, and (9) myocardial structure and function. There were also poster sessions on two evenings where over 50 selected posters were presented and rigorously discussed. The conference also included two international keynote speakers, Profs. Richard Harvey (Sydney, Australia), who presented unpublished data on a mouse *Nkx2-5* cis-regulatory mutant model of cardiac arrhythmogenic risk, and Maurice van den Hoff (Amsterdam, The Netherlands), who spoke about the development of the cardiac cushions, septa, and valves.

**Figure 1:**
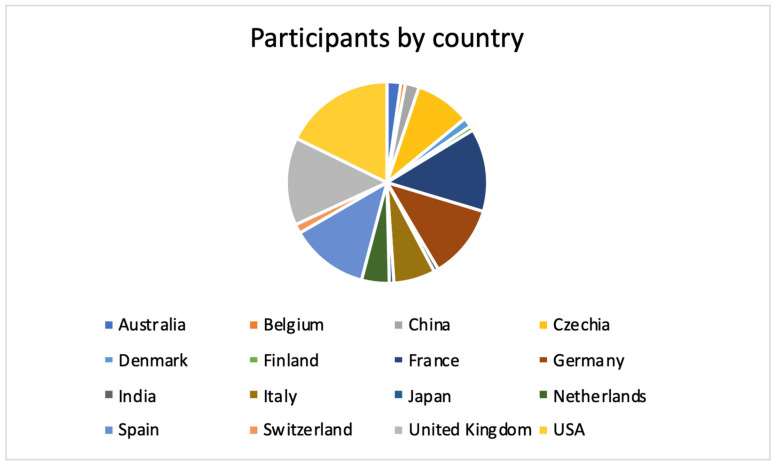
Breakdown of participants according to the country of their professional affiliation.

The meeting was extremely well attended, with 135 registered participants and six faculty members. The participants traveled to Prague from 16 different countries, of which 11 were from Europe and three from Asia; the single largest contingent arrived from the United States of America, and also Australia was represented (Figure 1).

The scientific committee, formed by the leadership of the ESC Working Group and the local organizer, evaluated a total of 87 submitted abstracts (Table 1). All the abstracts are available in the Abstract book, which is available online as Supplemental File.

## 2. Summary of the Platform Sessions

The form of the meeting was driven by the submitted abstracts. These were anonymized and judged by the Scientific Committee. The highest-scoring ones were grouped into platform sessions that covered the diversity of topics in the field. The highest number of abstracts was submitted for the cardiac progenitors session. The diversity of applied methodologies ranged from approaches using molecular and cellular biological techniques using cells and tissues (including organoids), as well as the whole organ and clinical studies. Many researchers presented single-cell sequencing data and spatial transcriptomics that brought a large quantity of spectacular data, sparking lively and intense discussions.

### 2.1. Pediatric Cardiology and Origins of Congenital Heart Diseasem (CHD) Joint Session

This first session represented a blend of two talks by pediatric cardiologists and two by basic scientists on the common theme. First, Viktor Tomek (Prague, Czech Republic) summarized the workflow of prenatal screening for developmental heart defects, and then Karel Koubsky (Prague, Czech Republic) followed up with an overview of treatment options for transposition of great arteries, including the challenges of the present day. In the first basic science talk, Stephanie Ibrahim (Marseille, France) speculated on the etiology of CHD through a combination of genetic (*Nkx2.5* haploinsufficiency) and environmental (maternal hyperglycemia) factors. Laura Bell (Oxford, UK) discussed transcriptomic and epigenomic changes in a model of iron deficiency in combination with *Notch1* mutations, suggesting novel options to ultimately reduce the occurrence of CHD.

### 2.2. Cardiac Progenitors I

The opening session of the second day of the conference started by presenting new data on the second heart field. The first talk by Miquel Sendra (Madrid, Spain) showed stunning live imaging of cells during the early stages of mouse cardiogenesis. Quantification of these recordings revealed shape changes that were complemented by single-cell RNA sequence data revealing differential expression of genes related to polarity and migration. Paul Palmquist-Gomes (Paris, France) postulated a role for *Vangl2* in modulating cell rearrangements in the second heart field, providing further insights into the role of the non-canonical Wnt planar cell polarity pathway in normal and abnormal heart morphogenesis. Rolf Bodmer (La Jolla, CA, USA) presented studies in Drosophila, suggesting an evolutionarily conserved heart-specific role for ribosomal proteins, which may be relevant in the pathogenesis of hypoplastic left heart syndrome. The session was concluded by the talk from Claudio Cortes (Oxford, UK) on cell dynamics in human interventricular septum morphogenesis.

### 2.3. Cardiac Regeneration

The presentations in this session highlighted the progress being made in understanding cardiac regeneration using different pre-clinical models. First, Gang (Rich) Li (Houston, TX, USA) demonstrated the role of YAP in inducing renewal of adult cardiomyocytes comparable to the regeneration competence of neonatal cardiomyocytes. Joaquim Nunes Vieira (London, UK) explored the role of lymphatic vessels and macrophages in the regenerative potential of the neonatal mouse heart following myocardial infarction. Sheila Caño-Carrillo (Jaen, Spain) concluded the session by showing evidence that the transcription factors *Mef2c* and *Nkx2.5* modulate *Gm14014* expression, playing a pivotal role in epicardial cell migration essential for heart regeneration in an ex vivo mouse ventricle explant cryoinjury model.

### 2.4. Cardiac Valves

This session showcased current areas of active research in the field of valve development. Rupal Gehlot (Bad Neuheim, Germany) presented an elegant study suggesting that Hand2 is a mechanosensitive transcription factor that mediates remodeling of the extracellular matrix in the embryonic ventricle and cardiac valves. Andrew Harvey (Charleston, SC, USA) talked about the role of transcription factor *Sox9* in epicardial attachment and invasion into the heart muscle, showing its importance for the establishment of the cardiac fibroblast cell population and valve formation and homeostasis. Ahlam Alquahtani (Newcastle, UK) focused on the role of primary cilia and showed that they are essential in the second heart field cell contribution to the aortic valve leaflets. Moreover, functional disruption of these cilia leads to bicuspid aortic valves. Finally, Brenda Giselle Flores-Garza (Madrid, Spain) identified in her study *Nherf2* as a *Notch* downstream gene with a potential role in cardiac valve development.

### 2.5. Mechanisms of CHD

This topic remains the foundation of these European cardiovascular development meetings, and the current platform session on this theme confirmed that it is still a highly relevant topic. Stefania Martucciello (Fisciano, Italy) described the interaction between Tbx1-Vegfr3 and showed that double heterozygote mice present with a peri-membranous intraventricular septal defect. Moreover, Tbx1-driven Vegfr3 inactivation also leads to outflow tract anomalies. Audrey Desgrange (Paris, France) presented a longitudinal analysis of heterotaxy with right isomerism using multi-modality imaging of Nodal mouse mutants. Interestingly she showed some plasticity of the ventricular position after the initial symmetry-breaking event of cardiac looping. Mingfu Wu (Houston, TX, USA) presented results on the expression of β1 integrins and showed that they regulate cellular behavior and cardiomyocyte organization during ventricular wall formation. The session was concluded by Tobias Bønnelykke (Marseille, France), who presented an intriguing mouse model with a bifid heart apex and proposed that ventricular septum formation depends on the folding and subsequent convergence of the right and left ventricular walls through a retinoic acid-dependent fusion mechanism.

### 2.6. Cardiac Progenitors II

Since the highest number of submitted abstracts identified with this topic, a second platform session was organized with a different perspective. Olga Lanzetta (Ischia, Italy) delivered a very informative presentation on the differentiation of the cardiopharyngeal mesoderm, reporting that TBX1 regulates chromatin accessibility and gene expression within an evolutionary conserved transcriptional module crucial for the development of the trunk and pharynx. José María Pérez-Pomares (Malaga, Spain) presented data on epicardial progenitor cell conversion into primitive epicardial cells and then to mesenchymal epicardial-derived cells. The main finding was that Wt1^High^/Itga4^High^ proepicardial cells represent bona fide progenitors of primitive epicardial cells and epicardially derived mesenchymal cells and display an unexpectedly high proliferative activity that is regulated by Wnt signaling. Elena Cano (Malaga, Spain) reported the discovery that coronary arteries originate from cells that have previously transitioned through a specific tip cell phenotype. Through the use of single-cell transcriptomics, they identified non-overlapping intramyocardial and subepicardial tip cell populations with differential gene expression profiles and regulatory pathways. Finally, Sabrina Kaminsky (Mannheim, Germany), who studied cardiomyocyte ploidy in different mouse heart chambers, showed that Slit signaling, functioning through GPC1, is required for cytokinesis and may contribute to chamber-specific differences in cardiomyocyte ploidy.

### 2.7. Epigenetics

This session brought together a variety of presentations discussing epigenetics from different angles. Virginia Roland (Bern, Switzerland) revealed the cell type signatures of cardiac cis-regulatory elements during development and demonstrated the critical functional contributions of cardiac enhancers of conserved transcription factors important for cardiac morphogenesis and for the interpretation of CHD-associated genomic variants. Nancy Stathopoulou (Oxford, UK) identified Chd7 targets during the early stages of cardiac commitment and differentiation through genome-wide profiling and transcriptomics on *Chd7* conditional mutants. Radha Kulkarni (Bad Neuheim, Germany) studied epicardial–myocardial cell interactions in the ventricle during cardiac morphogenesis and observed that epicardial cell attachment plays a role in establishing cardiomyocyte cell polarity and trabecular development. Moreover, interactome analysis of *wt1a^−/−^* larval zebrafish hearts revealed candidate genes responsible for establishing cell polarity, among which *jam2b*. Duncan Sparrow (Oxford, UK) then finished by presenting results showing that maternal valproic acid exposure perturbs neural crest cell migration in mice and presented preliminary data on single-cell epigenomic data to unveil the mechanism by which valproate exposure causes heart effects.

### 2.8. Cardiac Conduction and Arrhythmias

The developmental origins of some arrhythmias are well recognized, and current research strives to provide more mechanistic explanations for these pathologies. Jeff Steimle (Houston, TX, USA) suggested that reduced Pitx2 in the left atrium leads to increased left-sided Wnt signaling, resulting in left atrial remodeling and increased arrhythmia susceptibility with age. These findings could provide new insights for both atrial fibrillation pathogenesis and therapeutic strategies for prevention. Marco Tarasco (Bad Neuheim, Germany) showed that constitutive overexpression of the voltage-gated sodium channel Scn5lab in zebrafish atrial cardiomyocytes leads to arrhythmia and induces fibrosis, establishing a new zebrafish model of atrial cardiomyopathy that resembles many of the phenotypes observed in patients with atrial fibrillation. The final presentation in this session by Kelly Smith (Melbourne, Australia) identified the Tmem161b requirement for the maintenance of mammalian cardiac rhythm and its interactions with key regulators of intracellular Ca^2+^ handling.

### 2.9. Myocardial Structure and Function

The talks in the final platform session of this year’s meeting mostly focused on cardiomyopathies. Cristiana Dondi (San Diego, CA, USA) studied the nutrient sensor CRTC and sarcalumenin/thinman in *Drosophila*, revealing that they represent a new pathway in cardiac hypertrophy and may also play a more general role in muscle cell maintenance [2]. Millie Fullerton (Newcastle, UK) showed that conditional deletion of *Slc5a6* from cardiomyocytes results in the development of cardiomyopathy and electrocardiogram abnormalities in mice. The last presentation of the conference was delivered by Alba Pau-Navalón (Madrid, Spain) on the role of PRDM16 in MYBPC3-related hypertrophic cardiomyopathy and left ventricular non-compaction. Using CRISPR-based transgenic mouse models, she broadened our understanding of the pathogenesis of cardiomyopathies and presented the design of potential treatment strategies.

## 3. Poster Session and Awards

Many excellent posters, paralleling the topics of the oral presentations were presented. As always, both evening poster session were very well attended until the closure of the building. Intense scrutiny of unpublished data and lively provocative discussions were conducted with a wonderful collaborative spirit that often extended to local bars and restaurants. Much networking was undertaken, and many new collaborations were created.

The posters were judged by several teams of judges, who then convened to select the winner and runner-up of the poster prize. The prize for the best poster (Rychter award, honoring one of the local pioneers in cardiovascular development) went to Cristina Villa Del Campo (Madrid, Spain) for her poster entitled “Single cell transcriptomics of adult mononucleated cardiomyocytes reveals a single transcription factor that regulates cardiomyocyte polyploidization in mammalian hearts”. The poster prize for runner-up went to Louise Michel (Marseille, France) for the poster “Identification of an NKX2-5-dosage dependent progenitor cell population contributing to the Purkinje fiber network”.

There were also two awards for the best platform presentation, which were selected by the international scientific committee. The winner of the Pexieder award was Olga Lanzetta (Ischia, Italy), and the runner-up award went to Tobias Bønnelykke (Marseille, France).

There was one more prize awarded, which was selected by the entire audience of the meeting. This award, honoring our recently deceased colleague Robert P. Thompson, for the most intriguing research presented at the meeting was awarded to Millie Fullerton (Newcastle, UK) for showing how vitamin supplementation may prevent lethality in mice in which the mitochondrial multivitamin transporter is deleted.

These awards, as well as the costs of the Keynote lecturers and faculty travel, would not have been possible without the generous support of our sponsors: Additional Ventures, SciMedia, BrainVision, Journal of Cardiovascular Development and Disease, Animalab, Biotechne, Escimeda, Helago, LifeM, and Schoeler Instruments. Three travel grants were awarded by the ESC to junior members of the working group, based upon abstract grading by the scientific committee.

**Figure 2:**
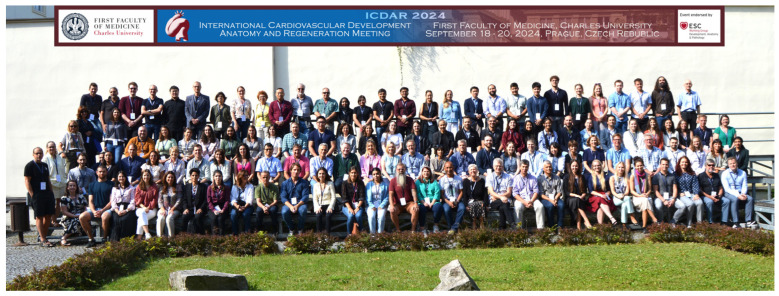
Group photo of the meeting participants taken on 19 September 2024.

The attendance is documented in the group picture taken at lunch time on Thursday (Figure 2).

## 4. Conclusions and Perspectives

Despite the interruptions of the COVID pandemic, it is obvious that the European cardiovascular development and regeneration community is alive and thriving. The quality of the meeting is recognized internationally and attracts participants from the rest of the world. The next ICDAR meeting is scheduled for 15–17 October in Granada, Spain, and will be organized by Diego Franco (Jaen, Spain).

## Figures and Tables

**Table 1:** Summary data on conference participation.

	ICDAR 2024
Total abstracts	87
Scientific sessions	19
Number of faculty	6
Total participants	135

## Data Availability

Abstracts from the meeting are available in the Supplemental File.

## Supplementary Materials

The following supporting information can be downloaded at: https://www.mdpi.com/article/10.3390/jcdd11120390/s1, Final version of the abstract book—Supplemental File.

## References

[1] Franco D., Ho S.Y., Kelly R.G. (2014). An Introduction to the ESC Working Group on Development, Anatomy and Pathology. J. Cardiovasc. Dev. Dis..

[2] Dondi C., Vogler G., Gupta A., Walls S.M., Kervadec A., Marchant J., Romero M.R., Diop S., Goode J., Thomas J.B. (2024). The nutrient sensor CRTC and Sarcalumenin/thinman represent an alternate pathway in cardiac hypertrophy. Cell Rep..

