# Supplementary material for: International Cardiovascular Development, Anatomy, and Regeneration (ICDAR) Community Meeting: Prague 2024
Source: J Cardiovasc Dev Dis. 2024 Dec 4;11(12):390. doi: 10.3390/jcdd11120390 (PMC11678715; doi:10.3390/jcdd11120390)
Supplement: Supplementary file 1 [file jcdd-11-00390-s001.zip › jcdd-3336872-supplementary.pdf]

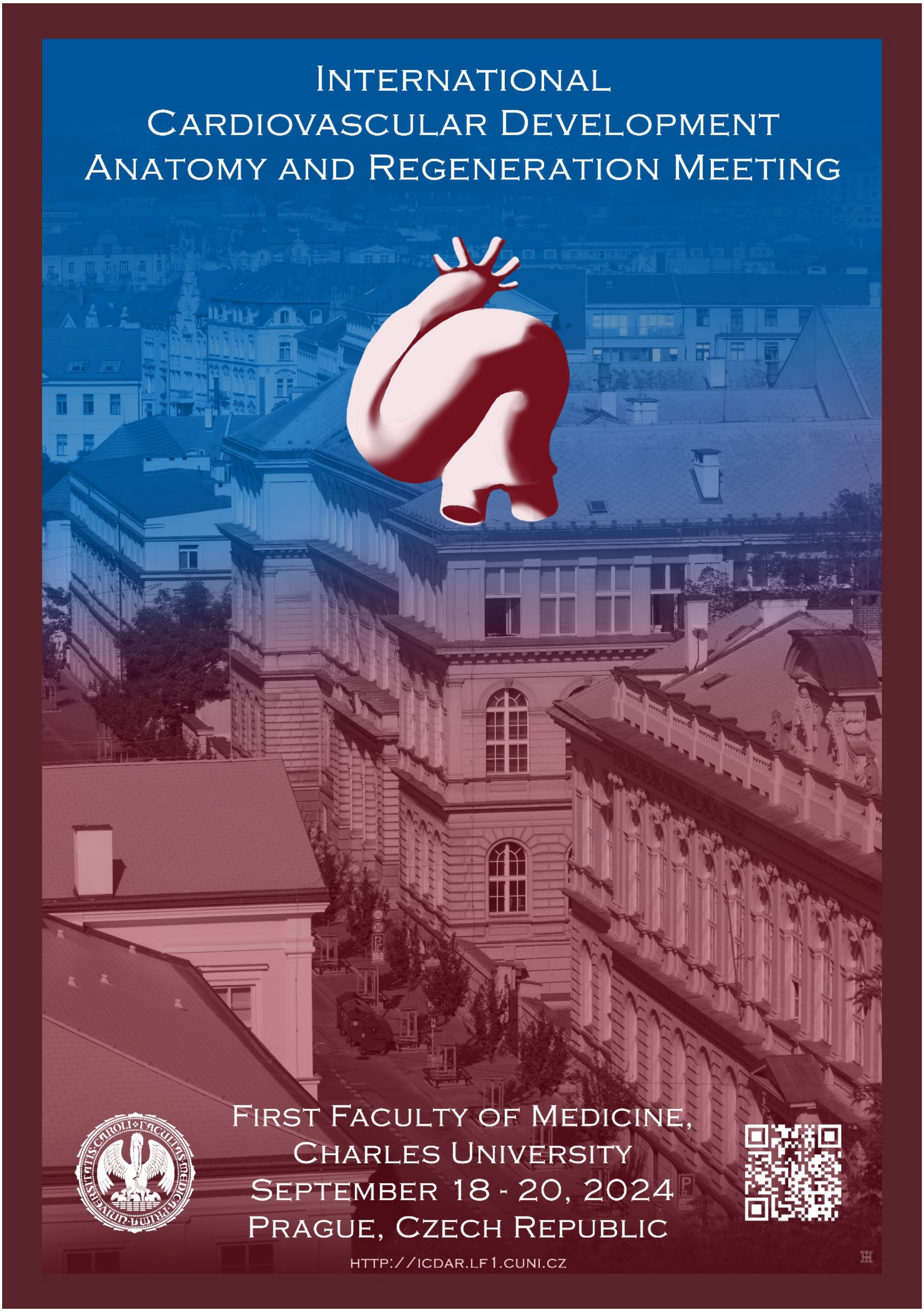

ABSTRACT BOOK

TABLE OF CONTENTS

WECLOME MESSAGE 1
ABOUT THE MEETING 2
CONGRESS VENUE AND PLAN 3-5
ABOUT PRAGUE 6-7
DINING OPTIONS 8
SCIENTIFIC PROGRAMME 9-11
KEYNOTE LECTURE 1 12
KEYNOTE LECTURE 2 13
ORAL ABSTRACTS 14-48
POSTER SESSION ABSTRACTS 49-108
INDEX TO ABSTRACTS 109-110
LIST OF PARTICIPANTS 111-116
NOTES 117-118

WELCOME MESSAGE

Prepare yourself for three days in the heart of Prague!

On Wednesday morning September 18, the meeting starts with a "hands-on" workshop on normal and congenitally malformed human hearts. Specimens will be presented by Drs. Lucile Houyel, Adrian Crucean, Viktor Tomek, and David Sedmera. There will also be the possibility to image a normal heart echocardiographically under expert guidance.

The next two and a half days, the meeting consist of cutting edge oral presentations selected from the abstracts, two evenings of posters with drinks and light snacks, two International Keynote speakers (Profs. Richard Harvey and Maurice van den Hoff), and lots of networking opportunities.

The meeting is directed towards fundamental and translational scientists in the field of cardiovascular development and regeneration and leans on the recently published ESC textbook of cardiovascular development (Eds José María Péres-Pomares and Robert Kelly).

All activities will be held at the Institute of Anatomy, U Nemocnice 3, Prague, Czech Republic.

The Networking dinner on Friday September 20th will be held at the historical University premises of Carolinum (Ovocny trh 560/5, Old Town of Prague).

For those interested in an out-of-town activity, a post-congress tour to Pruhonice chateu and gardens (UNESCO site) will be organized on Saturday 21st.

The local organizers:

David Sedmera (local host, abstracts administration)

Hana Kolesová (poster coordination and social events)

Alena Kvasilová (sponsors coordinator, webmaster)

FIRST FACULTY OF MEDICINE
Charles University

The meeting is organized under the auspices of

Prof. MUDr. Martin Vokurka, CSc., Dean of the First Faculty of Medicine, Charles University

International Scientific Committee:

Chair: **Assoc. Prof. Maurice van den Hoff**
Amsterdam, The Netherlands

Prof. Antonio Baldini
Naples, Italy

Dr. Marina Campione
Padua, Italy

Dr. Bill Chaudhry
Newcastle upon Tyne, United Kingdom

Prof. Dr. Anthony Firulli
Indianapolis, USA

Prof. Dr. Lars Allan Larsen
Kopenhagen, Denmark

Dr. Lucile Miquerol
Marseille, France

Dr. Emily Noel
Sheffield, United Kingdom

Dr. Adrian Ruiz-Villalba
Malaga, Spain

Assist. Prof. Anke Smits
Leiden, The Netherlands

Dr. Duncan Sparrow
Oxford, United Kingdom

Dr. Stephane Zaffran
Marseille, France

ABOUT THE MEETING

This meeting is based upon a community of scientists active in the field of cardiac development, anatomy, pathology and regeneration, who are organized in the working group Development, Anatomy and Pathology of the European Society of Cardiology (ESC). This year the meeting of this working group obtained endorsement of the ESC for its transparent abstract review process, educational value, outreach to clinical community. The ESC has also contributed several student travel bursaries to be awarded based upon abstracts submitted by junior working group members.

The history of the working group and its meetings was reviewed in JCDD by **Diego Franco** et al. (doi:10.3390/jcdd1010037), who will be organising next year's meeting in Granada (see the back cover of this booklet).

The first day of the meeting is organized in collaboration with the local paediatric cardiology community primarily from the Motol hospital and is accredited by the **Czech Medical Board** as a CME event. Those wishing to obtain credits must sign the attendance sheet at the entrance. Please claim credits only for the time actually spent in the meeting (45 minutes of activity = 1 credit, maximum 6 credits per day).

Event endorsed by

ESC
Working Group
Development, Anatomy
& Pathology

CONGRESS VENUE AND PLAN

Address:

[Charles University, First Faculty of Medicine, Institute of Anatomy]

U Nemocnice 3, CZ-128 00 Prague 2, Czech Republic

Conference venue: The institute is 450 m down the street (*Katerinska*, then blending to *U Nemocnice*) from the hotels. Below is the walking map from I. P. Pavlova (Metro line C, the red one; take the first exit to your left, and cross *Sokolska* with care):

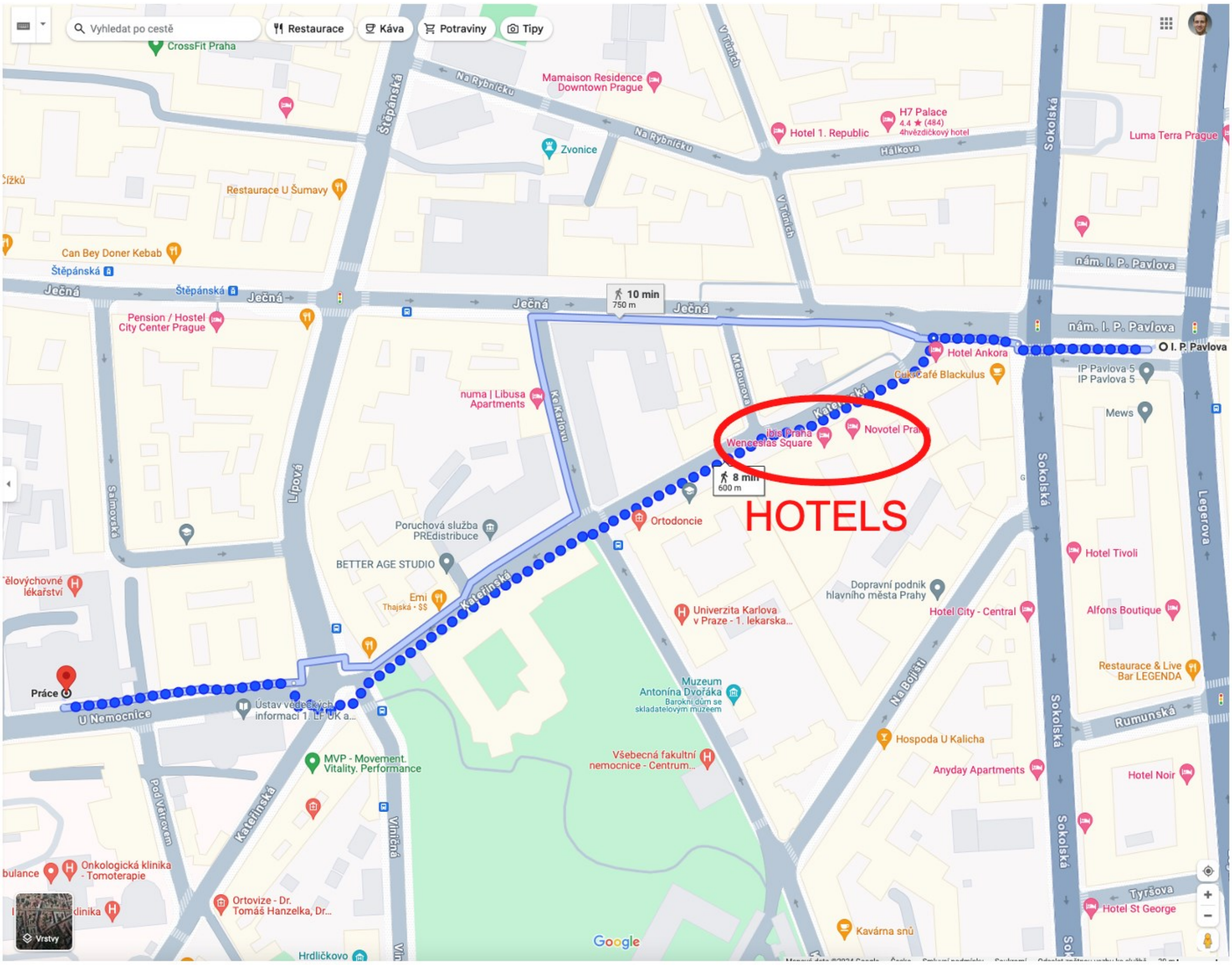

If you stay at another hotel, the Institute can also be reached from the other side – metro station *Karlovo Namesti* on the yellow line (B). Use the **exit** *Karlovo Namesti*, then **E2**.

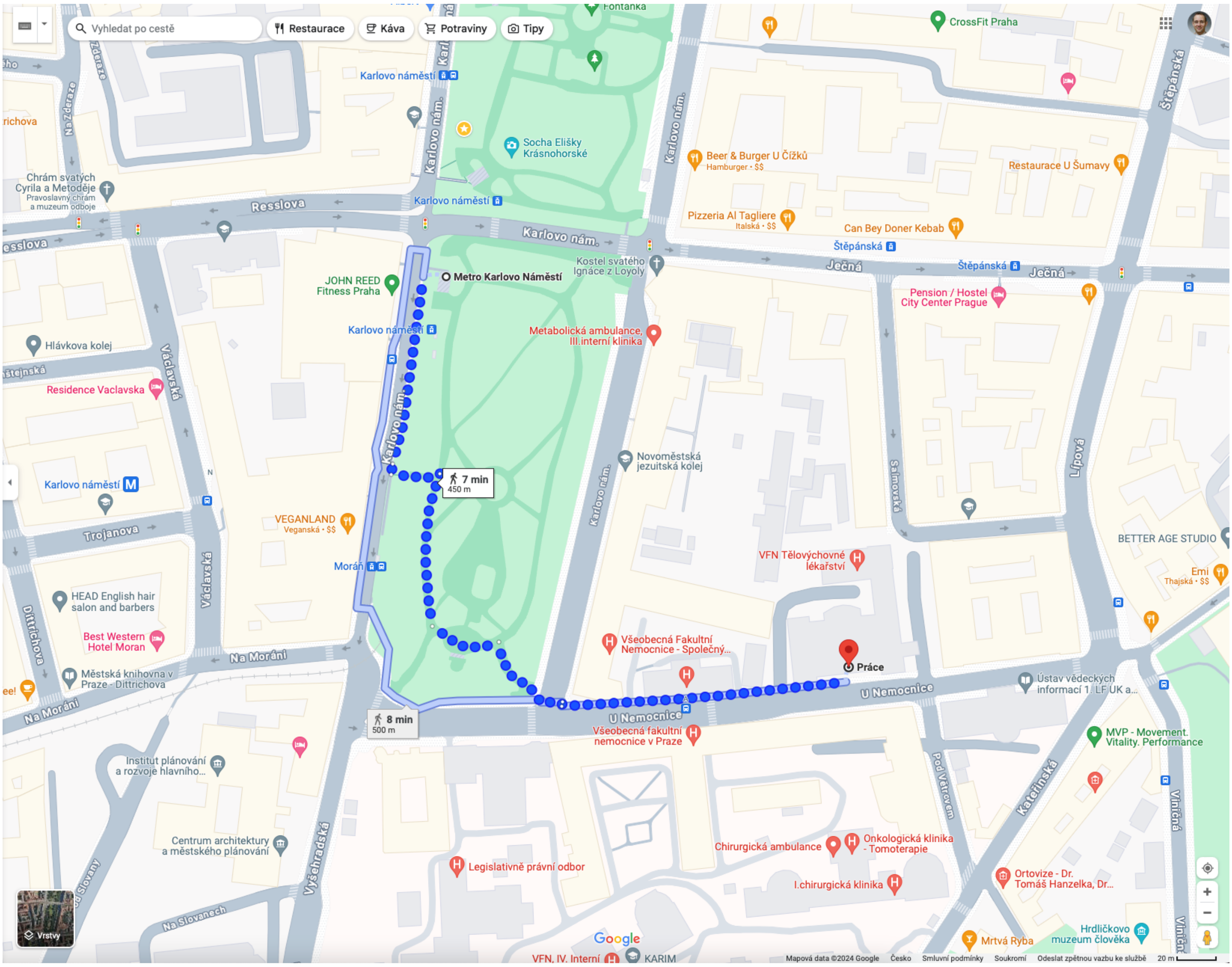

If you have any questions or need help, please email icdar@lf1.cuni.cz.

FLOOR PLANS OF THE INSTITUTE OF ANATOMY

MAP OF THE GROUND FLOOR OF THE DEPARTMENT OF ANATOMY

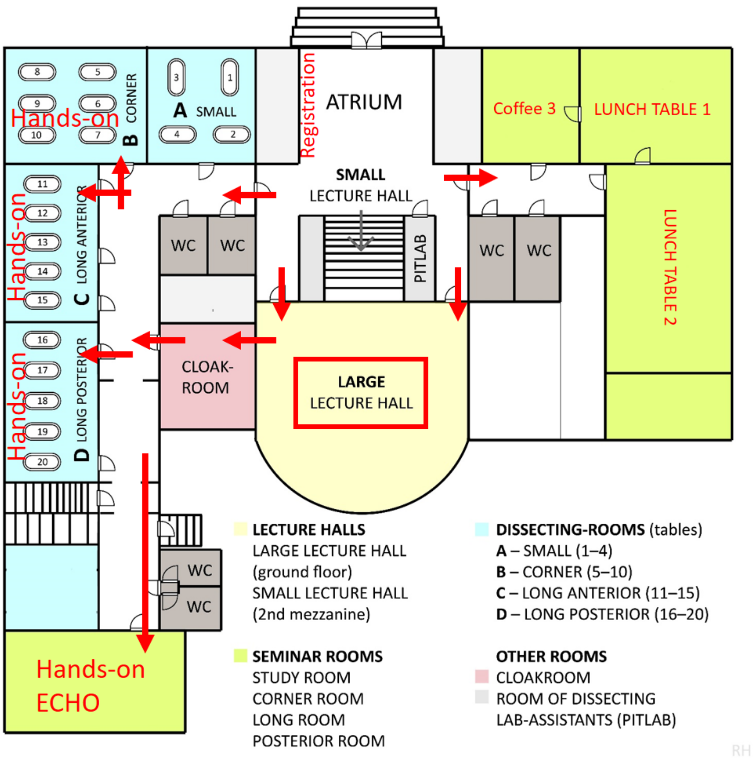

FIRST FLOOR – POSTER SESSION

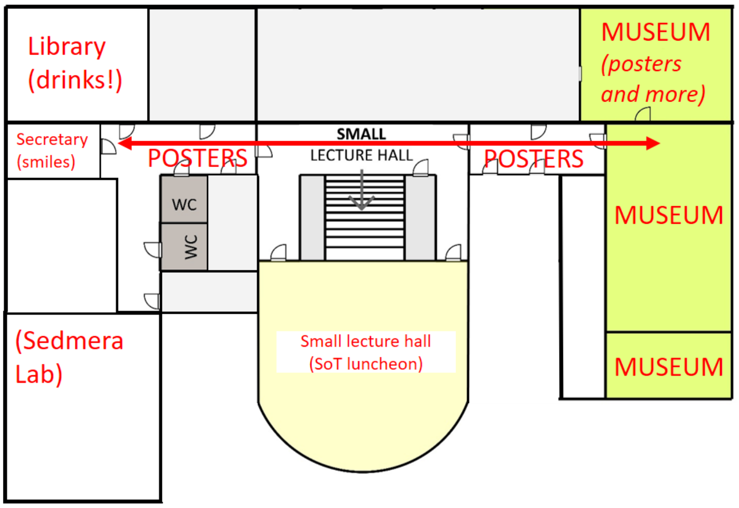

ABOUT PRAGUE

Prague is the capital and the largest city of the Czech Republic, home to 1.4 million people. It is located on the Vltava river and harbours a rich history Romanesque, Gothic, Renaissance and Baroque architectures which survived the violence and destruction over the centuries. **We recommend:** The Old Town, the Prague Castle, the Charles Bridge and Little Quarter.

There are also a few other sights close to the venue that are worth a visit (if you have a moment):

- Church of the St. Ignatius of Loyola (a wonderful example of baroque architecture and the former centre of the Jesuit order)
- Church of the St. Charles the Great (next to the Police museum)
- Church of St. Constantin and Metod (last stand of the paratroopers from Anthropoid who got Reinhard Heydrich in 1942)
- Dvorak's villa (a museum in the home of the great composer)
- The Dancing House (the famous curving office block)
- Vysehrad (the fortified medieval castle and exhibition)

How to get from the Airport to the hotels/venue: The airport is 17 km outside of the centre and official taxis or Uber are available, which cost between 30-40 euro (750-1000 CZK) and can take a lot of time. The cheapest and fastest option is public transportation. Buy a ticket for 90 minutes at 40 CZK (less than 2 euro) in bus 119 which leaves in front of terminal 2. Get off the bus at **Nadrazi Veleslavin** and take the Green A metro in the direction Skalka. Tickets are valid for a period of time and when transferring to another bus, metro or tram, they do not need to be re-stamped. Get off the metro at station Muzeum. In 10 minutes you walk to the hotels and in another 5 minutes you are at the conference venue.

Alternatively, you can change to the red (C) metro line and one station down the line (direction Haje) is **I. P. Pavlova** metro station. Then use the walking map on the previous pages.

If traveling directly to the venue, slightly faster alternative with fewer changes is to take the bus 100 to **Zlicin** (yellow metro line B, terminus), then take the metro train (direction Cerny Most) to **Karlovo namesti**. Then, walk to the institute (7 minutes).

Most international **trains** arrive at the Main Station (**Hlavni Nadrazi**), which is conveniently located on the red metro line (C), four minutes ride from **I. P. Pavlova** – take a train in the direction of Haje.

Limited parking is available in the courtyard of the Institute for those who come with their own transportation. Please contact the organisers in advance for detailed instructions.

Public transportation in general: Public transportation (metros, trams and buses) works very well! You can pay by cash or with your credit card either on the bus (receiving a paper ticket from the machine) or buy more tickets of whatever duration from an automated dispenser at the platform (cash or credit card). When you have bought your ticket, you MUST stamp to validate it when you begin your journey (boarding a bus, metro, or tram). They are valid for a period of time (30 min 30CZK, 90 min 40CZK, 72 hours 330CZK) and if transferring to another bus, metro or tram they do not need to be re-stamped.

The recommended hotels are at the red metro line (C) station I. P. Pavlova. It is right across the street (a major one, cross carefully and only on green light!) from the station taking the first exit to your left then veering into the Katerinska street, where you find both the Hotel Ibis Praha or Novotel Praha to your left.

We think that exploring the old historic part of Prague is best done on foot. We recommend a walk from the Namesti Republiky through Celetna to the Old Town Square, then across the Charles' bridge to the Little Quarter and up to Hradcany (Prague Castle). You can take a metro (green line from Hradcanska) back, if your feet are sore.

To beat the crowds of tourists why not head out before the meeting between 6-9 AM.

For jogging, you can try the park Folimanka, and for those who don't mind city running, the east bank of Vltava from Palackeho namesti south (as far as you dare). Just beware of the bikers and other users if you choose to run in the afternoon. Another alternative with views and without traffic is to circle the Vysehrad castle (below).

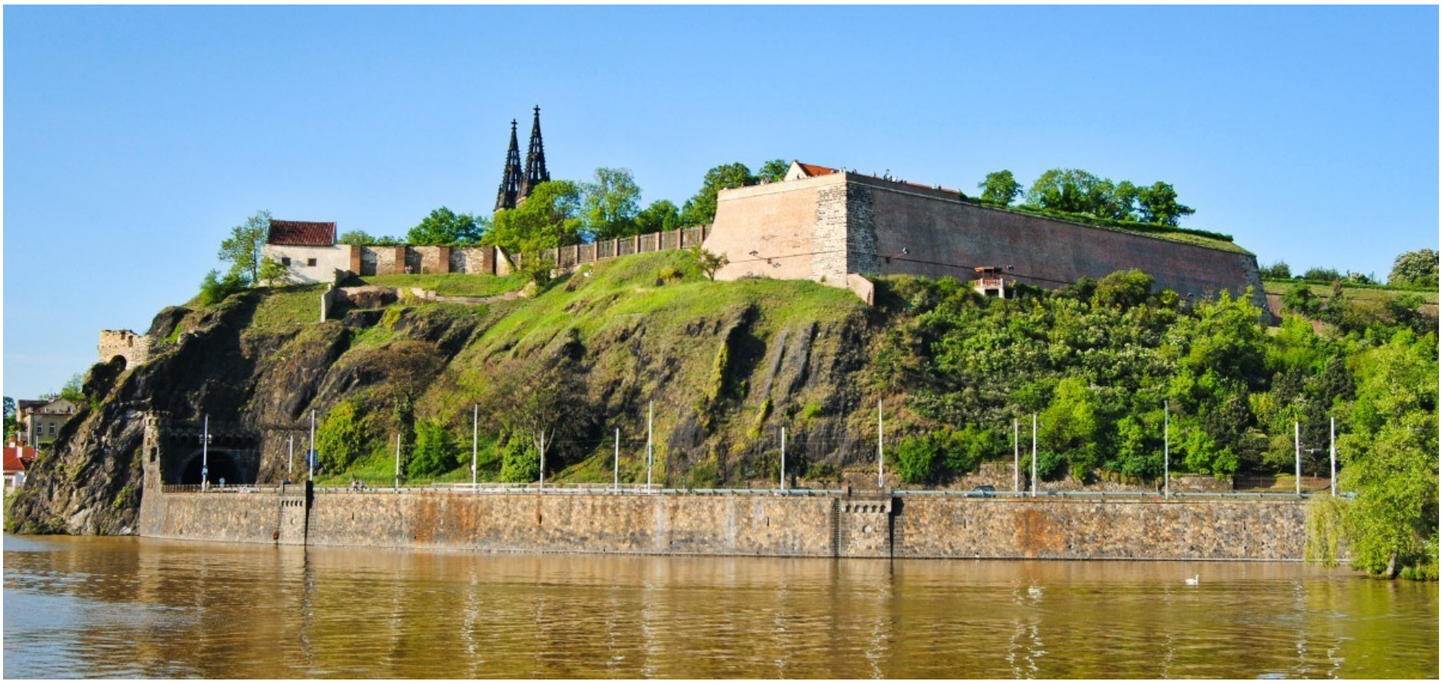

DINING OPTIONS

Below is a list of tested spots conveniently close to the venue. There are numerous other places in Prague to suit every taste with posted reviews, so go out and explore! An easy indication of a good value restaurant is that the primary menu is in Czech (you only need to ask for the English version, they have it, too).

Lunch options

Restaurants within the walking distance from the conference:

Bistro Fair Food Club – across the street in the yard – bistro with few lunch menus of Armenian cuisine. Seating also outside. https://www.fairfoodclub.cz/kontakt

Emi Thai restaurant – Thai restaurant good for lunch and to take away.

Pivovarsky dům Benedict – nice seating in the stylish brewery, good lunch and dinner option. Good selection of beers. https://www.pivo-dum.cz/en/home

Restaurace U Šumavy – typical Czech lunch menus. OK for lunch, but not for dinner. Good selection of beers. https://usumavy.cz/kontakt/

Doner Kebab – kebab place to take away, limited seating options.

Pizzeria Al Tagliere – original Italian cuisine, for lunch and dinner. https://altagliere.eatbu.com/?lang=en

Veganland – vegetarian and vegan restaurant (buffet-style). https://veganland.cz/en

Faust Restaurant – tasty Czech lunch menus. https://www.faustrestaurant.cz/contact/default

For those on tight budget, or those who want to be exactly sure what they eat, there is one regular (i.e., not just for tourists) supermarket at Karlovo Namesti: Albert.

Dinner options

Our tested restaurants downtown:

Pizzeria Kmotra – the first pizzeria in Prague. https://www.kmotra.cz/en/

Příčný řez – nice restaurant with seating also outside, better with reservation, also for lunch. https://www.pricnyrez.cz/en/

Restaurace U Voraře – stylish Jazz and Blues restaurant. https://www.restauraceuvorare.cz/

Manni´s Kitchen – Pakistani Halal restaurant and also take away. https://manniskitchen.cz/

Restaurace Tančící dům – stylish rooftop restaurant in the Dancing house, modern architecture, amazing view of Prague. Lunch and dinner, also bar. https://www.tancici-dum.cz/

Hostinec U Sadlu – medieval style restaurant in a gothic cellar, the other side of the city center. https://www.usadlu.cz

Pivovar U supa – right in the city center, live music, close to the seat of the Charles University. https://www.pivovarusupa.cz/en/homepage/

Jina Krajina – innovative Czech and international cuisine, also suitable for lunch. Good beers. https://reznicka.jinakrajina.cz/cs/poledni-menu/

Reservations for the dinner - just call the establishment, sometimes also through their website.

Google maps – restaurants work also nicely.

SCIENTIFIC PROGRAMME

Wednesday, September 18

9:00 – 12:30	Hands-on session on normal and congenitally malformed hearts<br>*Dr. Lucile Huyel, Adrian Crucean, Viktor Tomek, David Sedmera*
12:30 – 14:00	Lunch on your own
14:00 – 14:10	Opening Ceremony<br>**Platform Session I: Pediatric cardiology and Origins of Congenital Heart Disease joint session** ***(Chairs: Hana Kolesova & David Sedmera)***
14:10 – 14:30	O_01: Prenatal diagnosis of congenital heart defects and treatment options.<br>*Viktor Tomek*
14:30 – 14:50	O_02: Transposition of the great arteries: previously fatal congenital heart disease with excellent long-term prognosis in the current era.<br>*Karel Koubsky*
14:50 – 15:10	O_03: Maternal hyperglycemia and Nkx2.5 haploinsufficiency interact to affect heart development.<br>*Stephanie Ibrahim*
15:10 – 15:30	O_04: Do gene environmental interactions cause congenital heart disease?<br>*Laura Bell*
15:30 – 16:00	Coffee break, echocardiography hands-on demonstrations
16:00 – 17:00	**Keynote lecture I. by *Richard Harvey*** ***(Chairs: Sigolene Meilhac & David Sedmera)***<br>A mouse Nkx2-5 cis-regulatory mutant model of cardiac arrhythmogenic risk.
17:00 – 19:30	**Welcome Reception and Poster Session I (*odd numbers will present*)**

Thursday, September 19

	**Platform Session II: Cardiac progenitors I** ***(Chairs: Antonio Baldini & Nancy Stathopoulou)***
9:00 – 9:20	O_05: Transition of epithelial properties during second heart field morphogenesis.<br>*Miquel Sendra*
9:20 – 9:40	O_06: The Planar Cell Polarity gene Vangl2 controls cell rearrangements in the mouse second heart field for outflow tract elongation.<br>*Paul Palmquist-Gomes*
9:40 – 10:00	O_07: Ribosomal Protein genes, a novel class of CHD candidates, regulate heart development by interacting with cardiogenic, ECM, splicing and nucleolar stress factors.<br>*Rolf Bodmer*
10:00 – 10:20	O_08: Dissecting cell dynamics in human interventricular septum morphogenesis.<br>*Claudio Cortes*
10:20 – 10:40	Coffee break
	**Platform Session III: Cardiac regeneration** ***(Chairs: Adrian Ruiz-Villalba & Paul Palmquist-Gomes)***
10:40 11:00	O_09: YAP Induces a Neonatal Like Pro-Renewal Niche in the Adult Heart.<br>*Gang Rich Li*
11:00 – 11:20	O_10: Lymphatic-macrophage crosstalk during neonatal mouse heart regeneration and transition to fibrotic repair.<br>*Joaquim Miguel Nunes Vieira*
11:20 – 11:40	O_11: Mef2c and Nkx2.5 divergent transcriptional regulation of chick WT1_76127 and mouse Gm14014 lncRNAs and their implication in epicardial cell migration.<br>*Sheila Caño-Carrillo*

11:40 – 12:00	Congress Photo
12:00 – 13:15	Lunch (Scientists of Tomorrow to convene in the Small Auditorium upstairs)
13:15 – 14:00	Business meeting (Great Auditorium)
	**Platform Session IV: Cardiac valves** ***(Emily Noel & Chris Derrick)***
14:00 – 14:20	O_13: Hand2 is required cell-autonomously in endocardial cells for cardiac valve formation.<br>*Rupal Gehlot*
14:20 – 14:40	O_14: SOX9 regulates epicardial attachment and invasion important for establishment of cardiac fibroblast lineage and atrioventricular valve homeostasis.<br>*Andrew Harvey*
14:40 – 15:00	O_15: Remodelling of supernumerary leaflet primordia leads to bicuspid aortic valve (BAV) caused by loss of primary cilia.<br>*Ahlam Alqahtani*
15:00 – 15:20	O_16: Nherf2: a Notch downstream gene with a potential role in cardiac valve development.<br>*Brenda Giselle Flores-Garza*
15:20 – 16:00	Coffee break
16:00 – 17:00	**Keynote Lecture II by Maurice van den Hoff** ***(Chairs: Antonio Baldini & Andy Wessels)***<br>Cushions, septa and valves.
17:00 – 19:30	Poster Session II (even numbers will present) with drinks
19:30 –	Dinner with your friends (a list of nearby restaurants is provided)

Friday, September 20

	**Platform Session V: Mechanisms of Congenital Heart Disease** ***(Chairs: Duncan Sparrow & Federico Tessadori)***
9:00 – 9:20	O_17: Tbx1-Vegfr3 interaction is required in cardiac morphogenesis.<br>*Stefania Martucciello*
9:20 – 9:40	O_18: Plasticity of ventricle position after heart looping in heterotaxy.<br>*Audrey Desgrange*
9:40 – 10:00	O_19: β1 integrins regulate cellular behavior and cardiomyocyte organization during ventricular wall formation.<br>*Mingfu Wu*
10:00 – 10:20	O_20: Investigating the role of retinoic acid signaling during morphogenesis of the muscular interventricular septum.<br>*Tobias Bønnelykke*
10:20 10:40	Coffee break
	**Platform Session VI: Cardiac progenitors II** ***(Chairs: Jeroen Bakkers & Richard Tyser)***
10:40 – 11:00	O_21: Single cell Multiomics and computational inference of cardiopharyngeal differentiation: the role of Tbx1.<br>*Olga Lanzetta*
11:00 – 11:20	O_22: WT1/ITGA4 epicardial progenitor conversion into expanding epicardial-derived cells: non-canonical WNT signalling mediated early progenitor proliferation.<br>*Jose Maria Perez Pomares*
11:20 – 11:40	O_23: Intramyocardial sprouting tip cells specify coronary arterialization.<br>*Elena Cano*
11:40 – 12:00	O_24: The role of Slit signaling in chamber-specific cardiomyocyte polyploidization.<br>*Sabrina Kaminsky*
12:00 – 13:00	Lunch

Platform Session VII: Epigenetics ***(Chairs: Anke Smit & Amelia Aranega)***

13:00 – 13:20 O_25: Functional architecture of cardiac TF regulatory landscapes in control of mammalian heart development.
Virginia Roland

13:20 – 13:40 O_26: CHD7 modulates Second Heart Field gene expression via binding to novel distal cardiac enhancers.
Nancy Stathopoulou

13:40 – 14:00 O_27: Investigating epicardial-myocardial interactions in the ventricle during cardiac morphogenesis.
Radha Kulkarni

14:00 – 14:20 O_28: Maternal valproic acid exposure perturbs neural crest cell migration in mice.
Duncan Sparrow

14:20 – 15:00 Coffee break

Platform Session VIII: Cardiac conduction and arrhythmias ***(Chairs: Marina Campione & Gaetano D'Amato)***

15:00 – 15:20 O_29: Left-right differences in Wnt inhibition underlie a pro-fibrotic microenvironment and atrial fibrillation predisposition for Pitx2 deficiency.
Jeff Steimle

15:20 – 15:40 O_30: Constitutive overexpression of the voltage-gated sodium channel Scn5lab in atrial cardiomyocytes leads to arrhythmia and induces fibrosis.
Marco Tarasco

15:40 – 16:00 O_31: Tmem161b is required in for the maintenance of mammalian cardiac rhythm and interacts with key regulators of intracellular Ca2+ handling.
Kelly Smith

Platform Session IX: Myocardial structure and function ***(Chairs: José Luis de la Pompa & Joaquim Nunes Vieira)***

16:00 – 16:20 O_32: The nutrient sensor CRTC and Sarcalumenin/Thinman represent a new pathway in cardiac hypertrophy.
Cristiana Dondi

16:20 – 16:40 O_33: Investigating a Novel Genetic Cause of Cardiomyopathy and Therapeutic Interventions.
Millie Fullerton

16:40 – 17:00 O_34: Exploring the Role of PRDM16 in MYBPC3-Related Hypertrophic Cardiomyopathy and Left Ventricular Non-Compaction in Mouse Models
Alba Pau-Navalon

17:00 – 17:15 Awards and closing of the meeting

18:00 Meet in front of the Institute for a walk through the historic center to the Networking Dinner location

18:30 – 22:30 Farewell networking dinner @Carolinum

Saturday, September 21

Optional tour of the Pruhonice Chateau and gardens (separate registration and payment on site, leaving at 9 AM from the hotels; please pre-register online so we can optimize transportation arrangements).

The ultrasound machine for the hands-on course and Wednesday afternoon demonstration is kindly provided by GE Healthcare.

GE HealthCare

KEYNOTE LECTURE 1

Professor Richard P. Harvey AM PhD FAA FAHMS FRS

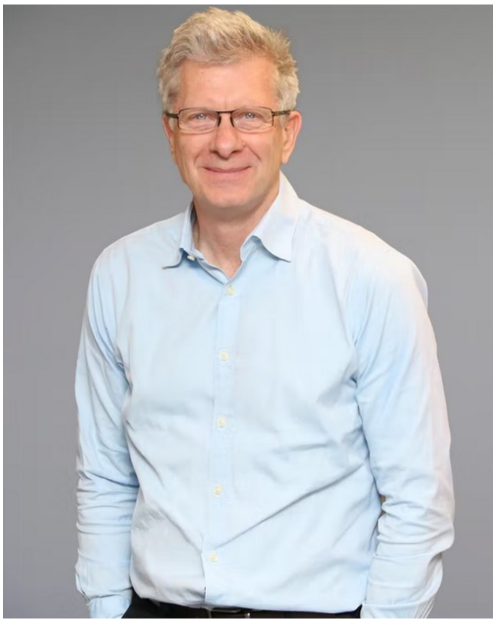

Professor Richard Harvey received his PhD in Molecular Biology in 1982 from the University of Adelaide, Australia. He undertook postdoctoral studies in molecular embryology at Harvard University with Doug Melton, before joining the Walter and Eliza Hall Institute of Medical Research in Melbourne, Australia in 1988, establishing an independent group. In 1998, he relocated to the Victor Chang Cardiac Research Institute in Sydney, taking up the inaugural Sir Peter Finley Chair at the University of New South Wales. He served as co-Deputy Director from 2003-2022. His research has focused on the genetic basis and systems biology of heart development and congenital heart disease pathophysiology, as well as adult cardiac stem and stromal cells, and the promise of heart regeneration in man.

A mouse Nkx2-5 cis-regulatory mutant model of cardiac arrhythmogenic risk

Nicholas Murray[1,2], Vikram Tallapragada[1,2], Osvaldo Contreras[1,2], Justin Phan[1], Eddy Kizana[3], Ann-Kristin Altekoester[1,4], Nicole Schonrock[1,5], Dave Humphreys[1], Gonzalo del Monte Nieto[1,6], Thomas Preiss[7], Rina Soetanto[7], Stefan Seemann[5,8], Lucile Miquerol[9], Jianxin Wu[1], Dirk Van Helden[10], Charles Cox[1,2], Vaibhao Janbandhu[1], Chris Thekkedam[1], Mirana Ramialison[1,11], Bharti Shewale[1], Robert Kelly[9], Adam Hill[1,2], Michael Feneley[1,2], Richard P Harvey[1,2].

[1]Victor Chang Cardiac Research Institute, Darlinghurst, Australia; [2]University of New South Wales, Kensington, Australia; [3]Westmead Institute for Medical Research, University of Sydney, Australia; [4]Faculty of Mathematics and Natural Sciences, University of Cologne, Germany; [5]Garvan Institute of Medical Research, Darlinghurst, Australia; [6]Australian Regenerative Medicine Institute, Monash University, Clayton, Australia; [7]John Curtin School of Medical Research, Australian National University, Canberra, Australia; [8]Department of Veterinary Sciences, University of Copenhagen, Denmark; [9]Marseille Developmental Biology Institute, Aix-Marseille University, Marseille, France; [10]School of Biomedical Sciences and Pharmacy, University of Newcastle, Australia; [11]Murdoch Children's Research Institute, Parkville, Australia

A collective of conserved cardiac transcription factors (TFs), including NKX2-5, orchestrates gene regulatory networks underpinning heart development. Despite recent advances, the regulatory logic guiding heart development, including roles for non-coding RNAs, remains poorly understood. We have characterised long non-coding RNA (lncRNA) genes expressed from the mouse and human *NKX2-5* loci. We discovered that alternative common genetic haplotypes disrupt the human NKX2-5 cis-regulatory and lncRNA landscape in complex ways, correlating with electrocardiogram (ECG) changes and increased risk of atrial fibrillation (AF) and atrioventricular (AV) conduction block in adults. These changes mirror those in patients with dominant heterozygous *NKX2-5* coding region mutations and in *Nkx2-5* heterozygous mice. To further explore these connections, we generated a transgenic mouse with a targeted *Nkx2-5* cis-regulatory deletion disrupting overlapping lncRNAs and a conserved genomic element, while preserving the coding region and known enhancers. Homozygous mice are phenotypically normal and have similar responses as WT mice to ischaemic and pressure overload stressors. However, they exhibit provokable AF and AV conduction block in the absence of other NKX2-5-related phenotypes, and show changes to immune cell signatures and an exacerbation of age-related cardiovascular decline. Molecular characterisation demonstrates a transcriptome and proteome crisis at the early phases of heart development, which is largely resolved. Our study suggests mechanisms whereby common or rare cis-regulatory genetic variants can impact congenital and adult disease risk.

KEYNOTE LECTURE 2

Dr. Maurice van den Hoff

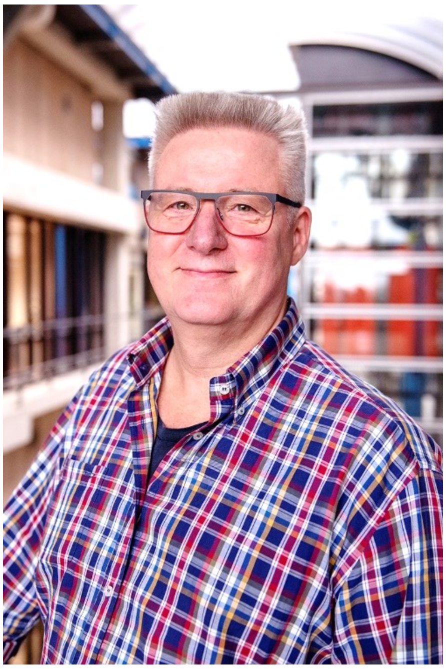

Maurice J B van den Hoff obtained his Master in Chemical Biology at the State University of Utrecht and 1989 and his PhD of the University of Amsterdam in 1994 on his thesis: Isolation and characterization of the rat Carbamoyl Phosphate Synthetase 1 gene. After his PhD he joined the department Anatomy and Embryology headed by Prof Lamers to start a training in cardiovascular development. He joined Prof Moorman and worked on the Molecular Cardiology Program of the Netherlands Heart Foundation, to train future Cardiologist in cardiac development and molecular and cellular biology. During this program he won several awards and became Associate Professor. Currently he works at the Department of Medical Biology, Amsterdam University Medical Centers, location Academisch Medisch Centrum, Universiteit van Amsterdam, which is headed by Prof Christoffels. His three main lines of research are human (cardiovascular) development, analysis of quantitative PCR data and establishing the role of Follistatin-like 1 in development and disease.
In 2017 he cofounded The Dutch Fetal Biobank. Embryonic and fetal tissues are voluntarily donated by women undergoing termination of pregnancy, having a premature labour or ectopic pregnancy. The tissue is collected within hours, completely anonymized and suitable for cutting edge molecular and image analyses.
Dr van den Hoff has been actively involved in the organization of both the annual Weinstein Cardiovascular Development and Regeneration meeting and the Working Group on Development, Anatomy and Development of the European Society of Cardiology annual meeting since 2010. Dr van den Hoff will retire from research in January 2025.

Cushions, septa and valves

In the Netherlands every day approximately 18 (3.8%) life born children present with a congenital abnormality, of which approximately three have a congenital cardiac abnormality. Moreover, more than 100 people die daily in The Netherlands due to cardiovascular disease. The main goal of research performed in my group was to understand the developmental mechanisms that are involved in normal and abnormal cardiac development and to understand the molecular response of the diseased heart.
This presentation will focus on the progress in our understanding of the development of the cardiac cushions and their derives, the septa and valves. Abnormal development of these structures accounts for approximately halve of the congenital cardiac abnormality.

Additional Ventures is a purpose-driven organization leveraging evidence-based research and deep subject matter expertise to make an outsized impact. https://www.additionalventures.org

O_01

Prenatal diagnosis of congenital heart defects and treatment options

Viktor Tomek, Jan Kovanda, Václav Chaloupecký, Jan Janoušek

Children´s Heart Centre, Second Faculty of Medicine, Charles University and Motol University Hospital, V Úvalu 84, 150 06, Prague 5, Czech Republic

Background: Congenital heart defects (CHD) are the most frequent developmental anomalies. Its occurrence is affected by several variables, mainly by prenatal choice of termination of pregnancy. We aimed to determine the pre- and postnatal incidence of major CHD in a setting of centralized health care system, the impact of cardiac screening on postnatal outcome and to discuss options for fetal treatment.

Methods: Total of 3,827 fetuses with antenatally diagnosed major CHD in the Czech Republic (population 10.7 million) between 1991 and 2021 were prospectively evaluated with known outcomes and associated comorbidities. Pre- and postnatal prevalence of CHD in unselected population was assessed by comparison with a retrospective analysis of all children born alive with major CHD in the same period (N=5,454), using national data registry.

Results and Conclusion: From 1991 to 2021, a total of 3,300,068 children were born alive, major CHD were diagnosed in 5454 children and detected in 3,827 fetuses of whom 1,646 (43.0%) were born, 2,069 (54.1%) were early terminated (TOP) and 112 (2.9%) died in utero. Prenatal detection rate increased from 6.2% to 82.8% ($P<0.0001$). TOP decreased from 70% in 1991 to 43% ($P<0.001$) in 2021. Postnatal prevalence of major CHD was declining from 0.21 to 0.14 ($p<0.0001$) over the study period. The total incidence (combining prenatal detection of unborn fetuses with postnatal prevalence) of major CHD remained at 0.23%.

Fetal cardiac intervention can alter the outcome for some critical CHD like aortic stenosis. Based on personal experience and literature review, the treatment should focus on only a few selected fetuses.

In this cross-sectional study we found that overall combined pre- and postnatal incidence of major CHD did not change significantly during the 31-year-long study period. Postnatal prevalence of major CHD decreased significantly due to early terminations of pregnancies and intrauterine deaths.

Funding: Supported by the project of the Ministry of Health of the Czech Republic for conceptual development of research organization 00064203 (University Hospital Motol, Prague).

O_02

Transposition of the great arteries: previously fatal congenital heart disease with excellent long-term prognosis in the current era

Karel Koubský, Václav Chaloupecký

Children's Heart Centre, Second Faculty of Medicine, Charles University and Motol University Hospital, V Úvalu 84, 150 06 Prague 5, Czech Republic

Background: Transposition of the great arteries is a critical congenital heart defect characterized by the origin of the aorta from the right ventricle and pulmonary artery from the left ventricle. Resulting parallel systemic and pulmonary circulations lead to death in > 90 % of patients within the first year of life if untreated. Several types of surgical treatment have been introduced over time. The current method of choice is the arterial switch operation (ASO), in which both great arteries are cut and transferred to their correct positions. The procedure is usually performed in the neonatal period and the most challenging part is the transfer of the coronary arteries.

Methods: This single-center nationwide retrospective study included consecutive children who underwent ASO between 1990 and 2016 (n=605). Long-term outcomes were obtained by cross-mapping individual data with the National Death Registry and the National Registry of Cardiovascular Interventions for adults. A control group was randomly retrieved at a 1:10 ratio from the National Birth and Death Registries. Special attention was paid to coronary artery abnormalities and their effect on survival.

Results and Conclusion: Early mortality was 3.3% and late mortality was 1.7% during a median follow-up of 10 (interquartile range, 5–16) years. The probability of overall survival at 20 years after ASO was 94.9% compared with 99.5% in the background population (hazard ratio [HR] 15.6; 95% CI, 8.9–27.5, P<0.001). Independent multivariable predictors of worse survival were an intramural coronary artery (HR, 5.2; 95% CI, 1.8–15.2, P=0.002) and period of ASO 1990 to 1999 (HR, 4.6; 95% CI, 1.5–13.6, P<0.001). Fourteen patients (2.3%) required 16 coronary artery reoperations.

Long-term survival after ASO is excellent. Coronary artery reinterventions are rare. An intramural coronary artery was an independent predictor associated with a higher risk for death, regardless of the surgical period.

Funding: This work was supported by the Ministry of Health, Czech Republic—conceptual development of research organization, Motol University Hospital, Prague, Czech Republic 00064203.

O_03

Maternal hyperglycemia and *Nkx2.5* haploinsufficiency interact to affect heart development

Stephanie Ibrahim*[1], Gwenaelle Collod[1], Nikita Ved[2], Clotilde Ferreira[1], Ashcroft Frances[2], Anne-Karin Kahlert[3], Bernard Thienpont[4], Duncan Sparrow[2], Sonia Stefanovic[1]**

[1] *Aix Marseille Univ, INSERM, Center for Cardiovascular and Nutrition Research, U1263, Marseille, France*

[2] *University of Oxford, Department of Physiology, Anatomy and Genetics, Oxford, United Kingdom*

[3] *Klinik für angeborene Herzfehler und Kinderkardiologie, Kiel, Germany*

[4] *Laboratory for Functional Epigenetics, Department of Human Genetics, KU Leuven, Leuven, Belgium*

Background: The maternal environment influences the occurrence of cardiac malformations by altering both cell fate and crucial signaling pathways involved in embryogenesis. Genetics contributes for a minority of these malformations but experimental evidence for gene-environment interactions is still limited. Maternal diabetes is associated with a fivefold increase in the prevalence of congenital heart disease (CHD), but the underlying molecular mechanisms are unclear. We hypothesized that genetic susceptibility to heart defects combined to maternal hyperglycemia is associated with changes in transcriptomic and epigenetic landscape causing CHD.

Methods: We used a novel tamoxifen-inducible transgenic mouse model (Kir6.2-V59M) of pregestational diabetes. Diabetic females were bred with *Nkx2.5*$^{GFP/+}$ males to generate embryos/fetuses predisposed to CHD and exposed *in utero* to maternal diabetes. We characterized the different cardiac phenotypes through High Resolution Episcopic Microscopy. We performed RNA-seq to better characterize the observed cardiac phenotype. Some of these findings have been confirmed at the protein level, showing increased fibrosis. Moreover, we used a single cell Multiome sequencing approach to profile the transcriptome and open chromatin landscape of E9.5 cardiac progenitor cells.

Results and Conclusion: We observed that fetuses exposed to maternal glycemic stress had outflow tract defects, septal defects, and thin myocardium. Higher incidence of hypoplastic left or single ventricle was observed in *Nkx2.5*$^{GFP/+}$ compared to *Nkx2.5*$^{+/+}$ E15.5 fetuses both exposed to maternal diabetes. Interestingly, our single cell data showed a downregulation of genes involved in specification of first heart field cardiomyocytes in *Nkx2.5* haplo-insufficient E9.5 hearts upon exposure to maternal diabetic milieu. Our data further revealed that these alterations in early gene expression is likely due to dysregulation of epigenetic remodelers. These findings highlight gene-environment interaction between *Nkx2.5* haploinsufficiency and maternal hyperglycemia. This interaction primarily affects the formation of the left ventricle by inducing changes in the epigenetic imprint in early cardiac progenitors.

Funding: Supported by FRM FDT202404018474 to SI, ANR19 ERA-CVD-0002 and ANR-20-CE13-0018 to SS.

O_04

Do gene environmental interactions cause congenital heart disease?

Laura E Bell[1], Victoria S Rashbrook[1], Selina Tsai[1], Paul Young[2], David Humphreys[2], Eleni Giannoulatou[2] and Duncan B Sparrow[1]

[1] *Department of Physiology, Anatomy and Genetics, University of Oxford, Oxford, UK*
[2] *Victor Chang Cardiac Research Institute, New South Wales, Australia*

Background: Congenital heart disease (CHD) is one of the most common types of birth defect affecting around 1 in 100 births in the UK. Genetic and environmental factors are both associated with CHD, but penetrance varies suggesting that most cases cannot be explained by genetic or environmental factors alone. Therefore, we hypothesis that some CHD cases are caused by a gene-environmental interaction (GxE). We aim to show that a ‘two-hit’ phenomenon is required for a defect to occur, whereby an embryo carrying a genetic predisposition to CHD is exposed to an environmental risk factor in utero.

Methods: To test this hypothesis, we mated iron deficient (ID) female mice to males which carry mutations in the *Notch1* gene which is associated with CHD. We used high-resolution episcopic microscopy (HREM) to analyse the heart and gross morphology of E15.5 embryos. To investigate the underlying transcriptomic and epigenomic changes driven by GxE, we performed multiomic ATAC and gene expression analysis on nuclei isolated from dissected heart regions of E9.5 embryos.

Results and conclusion: Looking at the gross morphology, we saw a significantly higher proportion of abnormalities including eye defects, subcutaneous oedema, blood in the lymphatic system and limb defects in *Notch1*$^{tm1.1Agt+/-}$ + ID embryos when compared to wildtype + ID and control *Notch1*$^{tm1.1Agt+/-}$ embryos. We also noticed that a large proportion of these *Notch1*$^{tm1.1Agt+/-}$ + ID embryos had both heart and limb defects, including syndactyly, oligodactyly, or a combination of both. This is similar to the phenotype of the human condition Adams Oliver Syndrome, some cases of which are caused by *NOTCH1* haploinsufficiency. Over 90% of *Notch1*$^{tm1.1Agt+/-}$ + ID embryos had a peri-membranous ventricular defect, with over half of these also having outflow tract alignment defects, which is highly significant when compared to wildtype + ID embryos. We also saw significant defects in aortic arch formation in *Notch1*$^{tm1.1Agt+/-}$ + ID when compared to wildtype + ID embryos, including a narrowing of the vessels and interrupted aortic arches.

We have seen a more severe phenotype with *Notch1*$^{tm1.1Agt+/-}$ + ID embryos than either wildtype + ID or *Notch1*$^{tm1.1Agt+/-}$ embryos. Our data suggests some human CHD may be caused by GxE, perhaps explaining the incomplete penetrance which is sometimes seen in affected families with a known genetic component. We are currently analysing our single nuclei multiome data, and we plan to validate this using gene/protein expression analysis of E9.5 embryos. By understanding how heart development is affected at a molecular level by environmental factors *in utero* can pave the way for public health campaigns to reduce the exposure to risk factors, ultimately reducing the number of CHD cases. This is especially important for those families who carry mutations in CHD associated genes.

Funding: Supported by Company of Biologists BSDS, Additional Ventures Single Ventricle Research Award, Additional Ventures Single Ventricle Expansion Award, BHF Senior Basic Science Research Fellowship, John Fell University Press Fund Award.

O_05

Transition of epithelial properties during second heart field morphogenesis

Miquel Sendra[1], Morena Raiola[1], Jorge Mañes-García[2], Jorge N. Dominguez[3], Miguel Torres[1] and Oscar Ocaña[1,3]

[1] *CNIC- Spanish Center for Cardiovascular Research, Madrid, Spain*

[2] *Centro de Biología Molecular, Madrid, Spain*

[3] *Universidad de Jaen, Jaen, Spain*

Early heart morphogenesis requires the coordination of distinct progenitor populations. The first heart field forms the primitive heart tube early on, while the second heart field (SHF) contributes to heart tube elongation later. The SHF is a polarized epithelium with specific basal properties, which enable cell migration and incorporation into the heart to supply progenitor cells. However, the mechanisms maintaining and modulating these SHF properties are poorly understood.

Using live imaging and a dense collection of mouse embryos with detailed cell segmentations, we observed changes in cell geometry and behavior along the anterior-posterior axis of the SHF between embryonic days E7.75 and E8.5 (from cardiac crescent to linear heart tube). Cell columnarity, defined as perpendicular elongation of the cell major axis within epithelial tissues, decreases towards the anterior side, near the heart tube outflow tract, indicating a transition from rigid to a more fluid epithelial state. Single-cell RNA sequencing revealed differential expression of genes related to polarity and migration including higher *Fgf8* expression in the anterior side.

Expression analysis and 3D quantification confirmed a countergradient of *Fgf8* (anterior) and *Meis1* (posterior). Functional experiments inhibiting the FGF signaling pathway expanded tissue columnarity anteriorly, while *Meis1/Meis2* double mutants exhibited disrupted heart development and decreased columnarity. FGF inhibition also expanded *Meis1* expression anteriorly. Moreover, E-cadherin levels decreased in *Meis* mutants, suggesting a role in maintaining epithelial columnarity. These findings suggest that *Fgf8* represses *Meis* in the anterior side of the SHF, restricting columnar epithelial properties to the posterior side and allowing a more mesenchymal-like behavior in anterior side cells prior to their incorporation into the heart tube. Characterizing this balance between epithelial stemness and differentiation is crucial to understand outflow tract defects and the dynamic properties of progenitor niches in epithelial tissues.

Funding: This research was supported by grant H2020-MSCA-ITN-2016-722427 from the EU Horizon 2020 program to M.T. M.S. was supported by a “la Caixa” Foundation PhD fellowship (LCF/BQ/DE18/11670014. O.H.O. is supported by the Ministerio de Ciencia e Innovación (grant RTI2018-097617-J-I00). J.N.D. received funding from grant 1380918 from the European Regional Development Fund Andalucía 2014-2020 Operating Program.

O_06

The Planar Cell Polarity gene Vangl2 controls cell rearrangements in the mouse second heart field for outflow tract elongation

Paul Palmquist-Gomes[1], Gaëlle Letort[2], José María Pérez-Pomares[3] and Sigolène M Meilhac[1]

[1] *Imagine - Institut Pasteur, Unit of Heart Morphogenesis, Paris, France*
[2] *Department of Developmental and Stem Cell Biology, Institut Pasteur, Université de Paris Cité, Paris, France*
[3] *IBIMA-Plataforma BIONAND and Department of Animal Biology, University of Malaga, Malaga, Spain*

Background: In the mouse, disruption of the Planar Cell Polarity (PCP) pathway is associated with congenital heart defects including double outlet right ventricle (DORV). This severe malformation disrupts the double blood circulation because both the aorta and pulmonary trunk are connected to the right ventricle. In the fly embryo, the PCP pathway coordinates cells in the plane of epithelia. This pathway is conserved in amniotes and inactivation of the core PCP component *Vangl2* disrupts the elongation of the outflow tract (OFT) in the embryonic heart tube, but it remains unclear by which mechanism the PCP pathway controls OFT elongation.

Methods: We used the CUBIC clearing method, whole mount immunofluorescence and lightsheet acquisitions to precisely quantify and characterise the heart phenotype of *Vangl2* constitutive and conditional mutants. We measured outflow tract (OFT) length in 3D and we microdissected the dorsal pericardial wall of E9.5 mouse embryos to characterize the spatial distribution of VANGL2 protein in heart progenitors. We applied a tissue cartography method and have developed new tools to quantify the cellular architecture of the second heart field in *Vangl2* mutants. We have analysed the expression levels of PCP genes in the second heart field and OFT from a published scRNAseq dataset (Gonzalez et al., 2022). In parallel, we microinjected DiI and specific inhibitors in the second heart field of chick embryos to assess whether specific cellular alterations in this tissue impact OFT elongation.

Results and Conclusion: We find that cardiac defects are uncoupled from neural tube defects. In the field of heart precursors, we observe that VANGL2 protein is not planar polarized and that the core PCP component *Celsr* is lacking. In contrast, VANGL2 accumulates in multicellular junctions. *Vangl2* mutant mice have a higher number of multicellular junctions in the second heart field, suggesting that *Vangl2* is required for rosette resolution. In chick embryos, pharmacological inhibition of rosette resolution is ongoing to assess the effect on heart progenitor ingression. Our work identifies a novel role of *Vangl2* in modulating cell rearrangements in the second heart field and provides novel insights into the role of the PCP pathway in heart morphogenesis relevant to congenital heart defects.

Funding: core funding from the Institut Pasteur, INSERM and Institut Imagine supports work in the Meilhac lab. PPG is a recipient of postdoctoral fellowships from Pasteur Roux-Cantarini and the Fondation Lefoulon-Delalande.

https://research.pasteur.fr/en/team/heart-morphogenesis/

O_07

Ribosomal Protein genes, a novel class of CHD candidates, regulate heart development by interacting with cardiogenic, ECM, splicing and nucleolar stress factors

Schroeder A[1], Nielsen T[1], Kervadec A[1], Theis J[2], Nelson TJ[2], Olson TM[2], Ocorr K[1], Vogler G[1], Colas A[1], Bodmer R[1]

[1] *Center for Genetic Disorders and Aging Research, Sanford Burnham Prebys, La Jolla, CA*

[2] *Div. Pediatric Cardiology, Dept. Pediatric and Adolescent Medicine, Mayo Clinic, Rochester, MN*

Background: Hypoplastic Left Heart Syndrome (HLHS) is a severe form of Congenital Heart Disease (CHD) characterized by an underdeveloped left ventricle. Its etiology is poorly understood but likely oligogenic. Sequencing efforts identified thousands of putative human disease variants, however, establishing genotype-phenotype relationships remains challenging.

Methods: To address this, we have performed high-throughput *in vivo* functional analyses of candidate genes using the fly heart, human iPSC-cardiomyocytes (hiPSC-CMs), with validation in zebrafish to interrogate their potential contributions in CHD/HLHS.

Results and Conclusion: Whole-genome sequencing of HLHS proband-parent trios with poor clinical outcome and GO enrichment analysis of prioritized genes revealed an over-representation of predicted-damaging variants in ribosomal protein (RP) genes. As expected, proliferation was reduced in RP variant-carrying iPSCs differentiated to cardiomyocytes (hiPSC-CMs) from probands compared to parents. Proliferation was similarly reduced upon RP gene knockdown in generic human iPSC-CMs or undifferentiated iPSCs. Interestingly, we identified genes/pathways whose expression were selectively altered in iPSC-CMs following *RPS15A* knockdown compared to undifferentiated iPSCs, highlighting a cardiac-specific transcriptomic response to RP disruption consistent with nucleolar stress. RP knockdown also reduced CM proliferation and contractility in zebrafish, and in *Drosophila* caused heart loss or severe cardiac defects. Temporal analysis of *RpS15a* or *RpL13* knockdown and in *Drosophila* revealed they are necessary to preprogram embryonic cardioblasts for later growth and remodeling; but after embryogenesis they are less essential, even though most cardiac growth and remodeling are yet to occur. Conversely, *RPS15A* knockdown-induced defects were significantly reversed (1) by *p53* co-knockdown in hiPSC-CMs and zebrafish, or (2) by YAP/*yorkie* overexpression or *myc* co-knockdown in flies. Probing for cardiac-specific RP functions, we found conserved, synergistic interactions between *RpL13/RPS15A* and core cardiogenic transcription factors *tinman/Nkx2.7* and *dorsocross/Tbx5a/TBX5* in *Drosophila,* zebrafish, and hiPSC-CMs. Finally, single-cell-RNAseq of *Drosophila* cardioblasts upon *RpL13* knockdown revealed dysregulation of genes involved in translation, splicing, extracellular matrix, and nucleosome organization, in addition to cardiogenesis, which was validated functionally by genetic interaction testing. Overall, our findings suggest a novel and conserved, heart-specific role for RP genes, likely in conjunction with cardiogenic genes, thus representing a new class of potential genetic effectors in CHD/HLHS.

Funding: Supported by NIH and a grant by the Wanek foundation at Mayo Clinic

O_08

Dissecting cell dynamics in human interventricular septum morphogenesis

Claudio Cortés, Matthew Stower, Shankar Srinivas, Paul R. Riley

Institute of Developmental & Regenerative Medicine, University of Oxford, Oxford, UK

Background: Cardiac septal defects are the most common presentation of congenital heart disease. Ventricular septal defects (VSDs) are one of the most prevalent in live births, yet our understanding of the etiology and progression of these defects is hampered by a lack of basic insight into the mechanisms underlying interventricular septum (IVS) formation.

Methods: We combined High-Resolution Episcopic Microscopy (HREM), lightsheet and confocal imaging to quantify the growth of the IVS in the developmental window spanning Carnegie Stage 14(CS14) to CS20 (5-8pcw, post conception weeks). We used laser-capture microdissections to collect tissue from 11pcw hearts. We also established a timelapse-compatible *ex-vivo* culture system of embryonic cardiac tissue.

Results and Conclusion: We describe rapid growth and increase in cell numbers in the growing human IVS. We then assessed proliferation and generated a model to track cell shape changes in the growing IVS, identifying a distinct population at the core of the IVS. We are also mapping cell contributions to the septum and ventricular free wall by retrospective lineage tracing, using somatic mutations as lineage tracers. Furthermore, using spatial transcriptomics, we uncover a specific left vs right IVS signature and identify new IVS-specific markers. To complement these fixed-tissue approaches, we imaged live cardiac tissue and applied a pixel-tracking pipeline to quantify growth dynamics in the IVS. Our results present the first systematic study of human IVS morphogenesis, furthering our understanding of human cardiac development and the origin of VSDs.

Funding: Supported by Wellcome Trust Human Developmental Biology Initiative (HDBI).

O_09

YAP Induces a Neonatal Like Pro-Renewal Niche in the Adult Heart

Rich Gang Li[1], Xiao Li[1], Yuka Morikawa[1], Francisco J. Grisanti-Canozo[2], Fansen Meng[1], Chang-Ru Tsai[2], Yi Zhao[1], Lin Liu[1], Jong Kim[1], Bing Xie[2], Elzbieta Klysik[2], Shijie Liu[3], Md Abul Hassan Samee[2], and James F. Martin[1,2]

[1] *The Texas Heart Institute, USA*

[2] *Department of Integrative Physiology, Baylor College of Medicine, USA*

[3] *Division of Molecular Cardiovascular Biology, Cincinnati Children's Hospital Medical Center, USA*

Background: After myocardial infarction (MI), adult mammalian hearts fail to regenerate, and the cardiac microenvironment is irreversibly disrupted. Inactivation of the Hippo signaling pathway in cardiomyocytes (CMs) induces heart renewal, and rebuilds the post-MI microenvironment.

Methods: We used single-cell RNA-sequencing combined with spatial transcriptomics to examine cellular relationships within the microenvironment of two murine cardiac renewal models: adult hearts expressing a constitutively active YAP (YAP5SA), and neonatal hearts subject to MI.

Results and Conclusion: We found in both models a conserved, renewal competent CM cell state with high YAP activity (CM2). CM2 colocalized with cardiac fibroblasts (CFs) expressing complement pathway component 3 (C3), and macrophages (MPs) expressing complement C3a receptor (C3ar1) to form a pro-renewal cellular triad. C3 and C3ar1 loss-of-function suppressed CM proliferation in both neonatal injured hearts and adult YAP5SA hearts, and indicated that C3a signaling between CFs and MPs was required to assemble the CM2, C3+ CF, and C3ar1+ MP cellular triad. Our results demonstrate that CM-YAP drives the coalescence of a pro-renewal niche, which requires complement pathway signaling, during *in vivo* cardiac renewal.

O_10

Lymphatic-macrophage crosstalk during neonatal mouse heart regeneration and transition to fibrotic repair

Joaquim Nunes Vieira[1], Konstantinos Klaourakis[2,3], Benjamin G. Chapman[3,3], Carla de Villiers[2,3], Mala Gunadasa-Rohling[2], Carolyn A. Carr[2], David G. Jackson[4], Daniela Pezzolla[5], Robin C. Choudhury[5], Paul R. Riley[2,3]

[1] *School of Cardiovascular and Metabolic Medicine & Sciences, King's College London, London, United Kingdom*
[2] *Department of Physiology, Anatomy and Genetics, University of Oxford, Oxford, United Kingdom*
[3] *Institute of Developmental & Regenerative Medicine, University of Oxford, United Kingdom*
[4] *MRC Human Immunology Unit, Weatherall Institute of Molecular Medicine, John Radcliffe Hospital, University of Oxford, United Kingdom*
[5] *Division of Cardiovascular Medicine, Radcliffe Department of Medicine, University of Oxford, Oxford, United Kingdom*

Background: In adult mice, myocardial infarction (MI) activates the cardiac lymphatics, which undergo lymphangiogenesis and function to drain interstitial fluid and traffic macrophages to mediastinal lymph nodes. This prevents oedema and reduces immune cell content improving cardiac function. Given the importance of the adult cardiac lymphatics in macrophage clearance post-MI, we investigated their role across the neonatal "regenerative window". At post-natal day 1 (P1), mice fully regenerate their heart following MI, in a pro-regenerative macrophage-dependent manner, whereas equivalent injury at P7 leads to scarring driven by pro-fibrotic macrophages. Thus, we hypothesised that lymphatics respond/function differently during this "window" to clear macrophage-specific subtypes.

Material and Methods: Lymphangiogenesis in wild-type, *Vegfr3*^{Lacz} and macrophage-specific *Lyve1* KO mice was characterised in intact/sham and MI neonatal hearts by adoptive transfer, immunostaining, confocal microscopy, and scRNA-sequencing.

Results & Discussion: Normal lymphatic expansion was evident in intact hearts until P16. The response to injury revealed limited lymphangiogenesis and minimal macrophage clearance from P1 versus P7 infarcted hearts. This is coincident with maturation of lymphatic endothelial cell (LEC) junctions across the neonatal period *via* transition from "zipper" (impermeable) to "button" (permeable)-type junctions. To gain molecular insight, we generated transcriptomic datasets from neonatal heart samples post-MI and observed altered signalling between LECs and macrophages. Finally, in mice lacking the lymphatic endothelial receptor-1 (*Lyve1)*, that exhibit impaired lymphatic transmigration of macrophages, MRI revealed a surprising impaired functional outcome in P1 mice post-MI. Furthermore, macrophage-deficiency of *Lyve1* during P1 injury revealed impaired heart regeneration (reduced neovascular response and function), suggesting a hitherto unappreciated role for LYVE1 in regulating the pro-regenerative function of tissue-resident macrophages (TRMs).

Conclusion: We reveal that cardiac lymphatics are developmentally compromised for clearance in early neonates, which enables retention of pro-regenerative TRMs. Moreover, LYVE1 macrophage plays an essential role in maintaining the regenerative phenotype via induction of revascularisation.

Funding sources: The British Heart Foundation and Wellcome Trust.

O_11

Mef2c and Nkx2.5 divergent transcriptional regulation of chick WT1_76127 and mouse Gm14014 lncRNAs and their implication in epicardial cell migration

Sheila Caño-Carrillo[1], Carlos García-Padilla[1], Amelia Aránega[1,2], Estefanía Lozano-Velasco[1,2], Diego Franco[1,2]*

[1] *Cardiovascular Development Group, Department of Experimental Biology, University of Jaen, Jaen, Spain*
[2] *Fundación Medina, Granada, Spain*

Background: Cardiac development is a complex developmental process. Soon after rightward looping, the embryonic heart becomes externally covered by the embryonic epicardium. A subset of these embryonic epicardial cells migrate and colonize the embryonic myocardium, contributing to distinct cell types. Our understanding of the molecular mechanisms that govern proepicardium and embryonic epicardium formation has greatly increased, including the discovery of non-coding RNAs. Our laboratory recently identified three distinct lncRNAs, adjacent to the Wt1, Bmp4 and Fgf8 chicken gene loci, with enhanced expression in the proepicardium that are distinctly regulated by Bmp, Fgf and thymosin ß4, providing support of their plausible implication in epicardial formation.

Methods: Expression of lncRNAs and their subcellular distribution were analyzed in different chicken and mouse tissues and cardiac cell lines. lncRNA transcriptional regulation was analyzed by using siRNAs and expression vectors of different transcription factors. Antisense oligonucleotides were used to inhibit Gm14014 expression and cardiac injury was induced ex vivo in mouse ventricle explants by cryoinjury. Furthermore, molecular and cellular techniques as well as viability and cell migration assays were conducted to investigate the biological function of Wt1_76127 and Gm14014.

Results and Conclusion: We demonstrated that Wt1_76127 in chicken and its evolutionarily conserved homologue Gm14014 in mice are widely distributed in different embryonic and adult tissues and distinctly regulated by cardiac enriched transcription factors, particularly Mef2c and Nkx2.5. Gm14014 is distinctly regulated in mouse ventricular cryoinjury ex vivo models, displaying a negative correlation with epicardial and epithelial to mesenchymal transition markers. Furthermore, silencing assays demonstrated that mouse Gm14014, but not chicken Wt1_76127, is essential for epicardial, but not endocardial or myocardial cell migration. Such process is governed by partnering with Myl9, promoting cytoskeletal remodeling. Our data evidence that Mef2c and Nkx2.5 modulate Gm14014 expression playing a pivotal role in epicardial cell migration essential for heart regeneration.

Funding: This work was supported by grants of the Ministerio de Innovación y Ciencia of the Spanish Government to DF (PID2022-138163OB-C32) and of the Consejería de Universidad, Investigación e Innovación of the Junta de Andalucía Regional Council to DF (ProyExcel_00409).

O_12

Loss-of-function CRISPR screen during cardiac differentiation of human stem cells identifies roles for ZNF711 and retinoic acid in balanced epicardial and cardiomyocyte lineage commitment

Verena Schwach[1], Rebecca R. Snabel[2], Carla Cofiño-Fabrés[1], Marijke Baltissen[2], Gert Jan C. Veenstra[2] and Robert Passier[1]

[1] *Department of Bioengineering Technologies, Applied Stem Cell Technologies Group, TechMed Centre, University of Twente, Enschede, The Netherlands Department of Molecular*

[2] *Developmental Biology, Radboud University, Faculty of Science, Radboud Institute for Molecular Life Sciences, Nijmegen, The Netherlands*

Background: CRISPR knockout screens enable quick gene knockout to uncover the roles of specific genes in cardiac differentiation and specification of human induced pluripotent stem cells.

Methods: Using a multiomic approach and gene-regulatory network inference, we focused on the regulators of epicardial, atrial and ventricular cardiomyocyte lineages and constructed a single-cell roadmap enabling direct comparisons of monolayer, cardiac embryoid body, and engineered heart tissue trajectories. In a next step, we performed a CRISPR knockout of LHX2, ZNF711 and ZNF503 in hPSCs and evaluated the effect on the differentiation towards atrial and ventricular cardiomyocytes, as well as epicardial cells.

Results and Conclusion: We constructed an atlas of cardiac differentiation under varying culture conditions, illustrating progenitor trajectories and cell states akin to those of the fetal heart. Through multiome and chromatin accessibility analyses, we identified potential regulators of both cardiomyocyte and non-cardiomyocyte lineages. Our study revealed that different culture conditions lead to distinct lineage commitment and differentiation trajectories, demonstrating the plasticity of cardiac progenitors and resulting in varying ratios of cardiomyocyte and non-myocyte lineages. Knockout of ZNF711 identified that ZNF711 is a regulatory switch ensuring cardiomyocyte commitment for a balanced differentiation towards epicardial and ventricular cardiomyocyte lineages during cardiac differentiation of hPSCs. In the absence of ZNF711, hPSCs failed to differentiate into ventricular cardiomyocytes, with progenitors instead predominantly differentiating into epicardial and other lineages. The presence of retinoic acid corrects this shift by compensating for the absence of ZNF711, and enhanced atrial cardiomyocyte differentiation. This highlights the interaction between ZNF711 and retinoic acid in cardiac lineage commitment.

Our findings feature the critical role of ZNF711 in cardiomyocyte differentiation and reveal that retinoic acid can mitigate the absence of ZNF711, underscoring a crucial interplay in the regulation of cardiac lineage commitment during cardiac differentiation of hPSCs.

Funding: This work was funded by a ZonMw TOP grant (projectnumber 91217061) and a ZonMw PSIDER Doorbraakprojecten grant (10250042110011).

https://appliedstemcelltechnologies.com/

O_13

Hand2 is required cell-autonomously in endocardial cells for cardiac valve formation

Rupal Gehlot[1*], Yanli Xu[1*], Marga Albu[1], Stefan Günther[2], Didier Y.R. Stainier[1]

[1] *Department of Developmental Genetics, Max Planck Institute for Heart and Lung Research, Bad Nauheim, 61231, Germany*

[2] *Bioinformatics and Deep Sequencing Platform, Max Planck Institute for Heart and Lung Research, Bad Nauheim 61231, Germany*

* Equal contributions

Background: Cardiogenesis requires the orchestration of several morphogenetic processes including valve formation. The bHLH transcription factor HAND2 has been implicated in cardiac valve formation in mouse; yet, little is known about its mechanism of action. To gain mechanistic understanding of the role of HAND2 during cardiac valve formation, we used the zebrafish model as it offers easy genome manipulation and accessibility for live imaging.

Methods: The early and severe cardiac defects in zebrafish *hand2* mutants has prevented lineage-specific and spatiotemporal study of its function. Here, we generated a floxed allele to investigate Hand2 function in endothelial/endocardial cells. In addition, in order to identify Hand2 effectors critical for cardiac valve formation, we analyzed the transcriptomes of *hand2* endothelial-cell specific knockout (KO) and *hand2* endothelial-cell specific overexpression (OE) (KO) embryonic hearts.

Results and Conclusion: High-resolution live imaging of a *hand2* reporter line revealed its high expression in endocardial cells, particularly in areas of high shear stress such as the atrioventricular (AV) canal. Consistently, loss of cardiac contractility/ blood flow leads to *hand2* downregulation, indicating that mechanical forces promote *hand2* expression. Using a *hand2* floxed allele, we found that knocking out *hand2* in *kdrl* expressing cells (i.e., endothelial cells) results in a complete loss of valve interstitial cells (VICs) and an increase in ventricular extracellular matrix (ECM). In contrast, *hand2* overexpression in endothelial cells led to an increased number of valve endocardial cells. To understand the mechanisms underlying these phenotypes, transcriptomic analysis of embryonic *hand2* endothelial-cell specific KO hearts was performed, revealing a number of disrupted pathways. Altogether, our data suggest that Hand2 is a mechanosensitive transcription factor that mediates ECM remodeling in the embryonic ventricle and cardiac valve formation. Current studies are focused on investigating the mechanism of action of Hand2 during VIC formation.

Funding: Max Planck Society

O_14

SOX9 regulates epicardial attachment and invasion important for establishment of cardiac fibroblast lineage and atrioventricular valve homeostasis

Andrew B. Harvey, Renélyn A. Wolters, Allison Trouten, Raymond N. Deepe, Jenna G. Drummond, Hannah G. Tarolli, Inara Devji, Jeremy L. Barth, Russell A. Norris, Andy Wessels

Department of Regenerative Medicine and Cell Biology, Medical University of South Carolina, Charleston SC, USA

Background: The epicardial lineage plays important and diverse roles in heart development contributing mesenchymal cell populations which support the growth and homeostasis of the myocardium, coronary vasculature, and atrioventricular (AV) valves. SOX9 is a transcription factor known to regulate epithelial-to-mesenchymal transformation, migration, proliferation, and extracellular matrix production, but much remains unknown about its role in the establishment and specification of the epicardial lineage.

Methods: We generated epicardial-specific *Sox9* knockout mice using the $Wt1^{cre}$ in combination with a $ROSA26^{mTmG}$ reporter construct to trace the epicardial lineage. We employed immunofluorescence analyses, bulk-, and single-cell RNA-sequencing to understand the morphological, cellular, and molecular changes associated with the loss of SOX9.

Results and Conclusion: Loss of SOX9 results in defective attachment of the epicardium to the underlying myocardium. Subsequently, there is a marked impairment in the invasion of epicardial derived cells (EPDCs) into both the myocardium and the AV valves. Single-cell sequencing reveals dysregulation of transcriptional regulators known to influence epicardial heterogeneity, activation, and differentiation of the epicardial-derived fibroblast lineage. An upregulation of smooth muscle cell markers in the epicardial cluster supports a model that SOX9 may be important for repression of smooth muscle cell identity before EpiMT. Data from this model also show a correlation between EPDC invasion and the development of the coronary vascular plexus within the ventricular wall. Postnatally, these mice develop disorganized and enlarged AV valves secondary to a drastic reduction of EPDCs in the parietal leaflets during development, highlighting the importance of the epicardial cells in AV valve development. Overall, our data supports an important role for SOX9 in the regulation of epicardial attachment and invasion, the establishment of the fibroblast lineage, and the epicardial contribution to the AV valves essential for matrix homeostasis.

Funding: AHA 23PRE1014420 (A.B.H.), NHLBI R01-HL122906 & R01-HL162913 (A.W.)

O_15

Remodelling of supernumerary leaflet primordia leads to bicuspid aortic valve (BAV) caused by loss of primary cilia.

Ahlam Alqahtani, Lorraine Eley, Jake Newton, Kimberley Macdonald, Leonor Lopez, Chloe Connolly, Cindy Rodrigues-Cleto, Javier Arias, Mashael Alaradi, Bill Chaudhry and Deborah J. Henderson.

Bioscience Institute, Newcastle University
Centre for Life, Central Parkway
Newcastle upon Tyne, NE1 3BZ, UK

Background: Bicuspid aortic valve (BAV), where two valve leaflets are found instead of the usual three, affects 1-2% of the general population and is associated with significant morbidity and mortality. Despite its frequency, the majority of cases remain unexplained. This is, at least in part, because there are two types of valve leaflet primordia: endocardial cushions and intercalated valve swellings (ICVS). Moreover, multiple progenitors contribute to the formation of these primordia. Genomic studies in mouse and human have suggested a correlation between BAV and malfunctional primary cilia. However, the precise requirement for cilia during early embryonic valvulogenesis remains obscure.

Methods: We disrupted primary cilia by deleting the ciliary gene Ift88 in the main progenitor cells forming the aortic valve using specific Cre drivers: Wnt1-Cre for neural crest cells, Isl1-Cre for second heart field cells (SHF); Tie2-Cre for endocardial-derived cells and Tnnt2-Cre for direct-differentiating SHF in the ICVS.

Results and Conclusion: The loss of Ift88, and thus primary cilia, from neural crest cells and endocardium are not required for normal aortic valve formation. However, primary cilia are essential in SHF cells for aortic valve leaflet formation, with half of $Ift88^{f/f}$;Isl1-Cre mutants presenting with BAV. As the valve leaflets are forming, 50% of the $Ift88^{f/f}$;Isl1-Cre mutants have two small leaflets in the position of the usual posterior leaflet, meaning that at this stage the aortic valve is quadricuspid. This quadricuspid valve then remodels to BAV by E15.5. Mechanistic studies demonstrate premature differentiation of SHF cells as the ICVS form, leading to the formation of a broadened ICVS that forms two posterior leaflets. This abnormality in the formation of the ICVS is associated with disruption of Notch-Jag1 signalling pathway. These data support a mechanistic link between quadricuspid and bicuspid arterial valve leaflets and show that primary cilia, via the Notch-Jag1 signalling pathway, regulate differentiation of SHF cells in the aortic valve.

Funding: Supported by British Heart Foundation (BHF).

O_16

Nherf2: a Notch downstream gene with a potential role in cardiac valve development

Brenda Giselle Flores-Garza[1,2], Luis Luna-Zurita[1,2], and José Luis de la Pompa[1,2]

[1] *Intercellular Signaling in Cardiovascular Development and Disease Laboratory, Centro Nacional de Investigaciones Cardiovasculares Carlos III (CNIC)*
[2] *Ciber CV*

Background: The sodium-hydrogen exchanger regulatory factor 2 (Nherf2) is a PDZ-containing regulatory protein expressed in the embryonic endocardium and in the adult kidney and small intestine. Genome wide association studies have linked the gene *Nherf2* (*Slc9a3r2*) to bicuspid aortic valve (BAV) formation and hypertension. Moreover, it has been shown that Nherf2 is crucial for endothelial homeostasis, regulating endothelial proliferation via the cyclin-dependent kinase inhibitor p27. In addition, we have shown that *Nherf2* directly responds to Notch signaling modulation in vivo and in vitro. Given these evidences, we sought to explore the role of *Nherf2* during cardiac development and its link to Notch signaling.

Methods: We generated a mouse model using CRISPR-Cas9 genetic edition to inactivate *Nherf2* and validated its effects by itself and in a Notch-sensitized background. Analysis of *Nherf2* expression was carried out through in situ hybridization and qPCR. Morphology analysis of *Nherf2* mutants was performed by H&E histology.

Results and Conclusion: *Nherf2* mutant mice are viable and do not exhibit gross differences with their littermates in morphology, behavior, or fertility. Heterozygous and homozygous *Nherf2* mice did not show obvious defects in heart morphogenesis at E16.5. *Nherf2* mice showed a decrease in ventricular septal defect (VSD) penetrance in a Notch-sensitized genetic background (*Notch1KO/+*). In situ hybridization of *Nherf2* heterozygous mice showed an increase in Bmp signaling, similarly to Notch signaling-deficient mice, suggesting that *Nherf2* is a downstream effector of Notch. This is the first study that explores the role of *Nherf2* as a downstream gene of Notch signaling and its role during cardiac development. Further experiments should be performed in order to understand the molecular mechanisms and implications of this novel gene.

Funding: FPI fellowship PRE2020-092102 from the Spanish Ministry of Science, Universities and Innovation (MCIU), grants MICIU/AEI/10.13039/501100011033: PID2019-104776RB-I00, PID2022-104776RB-100 and CB16/11/00399.

O_17

Tbx1-Vegfr3 interaction is required in cardiac morphogenesis

Stefania Martucciello[1], Sara Cioffi[2], Marchesa Bilio[2], Mariangela Cavallaro[1], Antonio Baldini[2,3], Elizabeth Illingworth[1]

[1] *Department of Chemistry and Biology, University of Salerno, Fisciano, Italy*

[2] *Institute of Genetics and Biophysics "ABT", Naples, Italy*

[3] *Department of Molecular Medicine and Medical Biotechnologies, University of Naples Federico II, Naples, Italy*

Background: *Tbx1* is the major gene involved in 22q11.2 deletion syndrome (22q11.2DS), the most common known genetic cause of congenital heart disease (CHD).

Rare variants of the *VEGFR3* gene cause cardiac outflow tract (OFT) abnormalities, including Tetralogy of Fallot, the most common cardiac defect found in 22q11.2DS patients. We have shown that in mice, TBX1 regulates *Vegfr3* in endothelial cells and the two genes interact strongly in brain vascularization and during cardiac lymphangiogenesis. We hypothesize that a similar genetic interaction is required for cardiac OFT development. Here, we sought to identify if and where such an interaction might occur.

Methods: We first tested for genetic interaction by intercrossing *Tbx1* and *Vegfr3* heterozygous mice and analyzing the hearts of *Tbx1+/-;Vegfr3*+/- embryos at E18.5. Next we used immunostaining and RNAscope to search for *Tbx1-Vegfr3* co-expression in the developing heart (wild type). Finally, we tried to pin-point the region where VEGFR3 function is critical for OFT development. To gain insights into this, we inactivated *Vegfr3* conditionally using two Cre drivers, $Tbx1^{Cre}$ and Mef2C-AHF-Cre, which have partially overlapping activation domains in cardiopharyngeal mesoderm of mid-gestation embryos. We analyzed the cardiac phenotype of conditional *Vegfr3* homozygous embryos at E18.5.

Results and Conclusion: We found that most *Tbx1+/-;Vegfr3*+/- embryos had a peri-membranous intraventricular septal defect, suggesting the existence of a genetic interaction. Moreover, *Tbx1*-driven homozygous conditional inactivation of *Vegfr3* caused intracardiac anomalies at 100% penetrance, including morphogenesis defects of the OFT, while Mef2C-AHF-Cre-induced homozygous *Vegfr3* mutants had normal hearts. Finally, we found that in the pharyngeal mesoderm (E8.5-E9.5) *Tbx1-Vegfr3* co-expression was limited to the aortic sac. Together, these data support the hypothesis of a genetic interaction between *Tbx1* and *Vegfr3* in OFT development and show the importance of VEGFR3 for cardiac morphogenesis.

Funding sources: Leducq Foundation; The Italian Ministry of University and Research (MUIR)

O_18

Plasticity of ventricle position after heart looping in heterotaxy

Audrey Desgrange[1,2], Emeline Perthame[1,2,3], Carmen Marchiol[1,4], Daphné Madec[1,2,5], Mohamed El Beheiry[6], Jean-Baptiste Masson[1,3,7], Olivier Raisky[1,5], Ségolène Bernheim[1,2,5], Lucile Houyel[1,2,5], Sigolène M. Meilhac[1,2]

[1] *Université Paris Cité ; Paris, France*

[2] *Imagine - Institut Pasteur Unit of Heart Morphogenesis, INSERM UMR1163, Paris, France*

[3] *Institut Pasteur, Bioinformatics and Biostatistics Hub, Paris, France*

[4] *INSERM U1016 and CNRS UMR8104 , Institut Cochin ; Paris, France*

[5] *Unité Médico-Chirurgicale de Cardiologie Congénitale et Pédiatrique, M3C-Necker, Hôpital Universitaire Necker-Enfant-Malades, APHP; Paris, 75015, France*

[6] *Sorbonne Université, Institut Curie, PSL Research University, CNRS UMR 168 Physico-Chimie Curie, F-75005, Paris, France*

[7] *Institut Pasteur, CNRS UMR 3571, Decision and Bayesian Computation, Paris, France*

Background: The heart functions in two parallel but asymmetric circuits, in which the right and left ventricles drive the pulmonary and systemic circulations, respectively. In the heterotaxy syndrome, abnormal left-right embryo patterning leads to a spectrum of severe congenital heart defects, including ventricle malposition. A postulate anchored in the clinical nomenclature, assumes that the looping direction of the embryonic heart tube determines ventricle position at birth. However, this has not been demonstrated experimentally.

Methods: Here, we performed a unique longitudinal analysis of heterotaxy with right isomerism, using multi-modality imaging of Nodal mouse mutants.

Results and Conclusion: Based on direct correlations and advanced statistics, we dissected the contribution of heart looping variations to specific structural heart malformations. We uncovered unexpected plasticity of ventricle position after heart looping, in 30% of revertant samples. Hearts of revertants are enriched in ventricular anomalies, a phenotypic association also observed in human patients with heterotaxy and right isomerism. Genetic tracing and topological associations do not support molecular reprogramming of ventricles but rather point to a novel step of heart remodelling after heart looping. Our work reveals distinct asymmetric events shaping the heart, beyond initial symmetry breaking in the node.

Fundings: Institut Pasteur, INSERM, ‘‘Investissements d'avenir’’ program (ANR-10-IAHU-01, ANR-10-LABX-73-01 REVIVE), Agence Nationale de la Recherche (ANR-21-CE14-0062-01), Additional Ventures, AXA Research Fund, Fondation Lefoulon Delalande.

O_19

β1 integrins regulate cellular behavior and cardiomyocyte organization during ventricular wall formation

Lianjie Miao[1], Yangyang Lu[1], Anika Nusrat[1], Luqi Zhao[1], Yongqi Xiao[1], Hongyang Guo[1], Yu Liu[1], Robert, J Schwartz[2], Ashok Kumar[1], C. Michael DiPersio[3], and Mingfu Wu[1*]

[1] *Pharmacological and Pharmaceutical Sciences, College of Pharmacy, University of Houston, Houston, TX 77204-5039*

[2] *Department of Biology and Biochemistry, University of Houston Sequencing and Gene Editing Core, University of Houston, Houston, TX.*

[3] *Department of Surgery, Albany Medical College, Albany, NY 12208*

Background: The mechanisms regulating the cellular behavior and cardiomyocyte organization during ventricular wall morphogenesis are poorly understood. Cardiomyocytes are surrounded by extracellular matrix (ECM) and interact with ECM via integrins. This study aims to determine whether and how β1 integrins regulate cardiomyocyte behavior and organization during ventricular wall morphogenesis in the mouse.

Methods and Results: We applied mRNA deep sequencing and immunostaining to determine the expression repertoires of α/β integrins and their ligands in the embryonic heart. Integrin β1 subunit (β1) and some of its ECM ligands are asymmetrically distributed and enriched in the luminal side of cardiomyocytes, and fibronectin surrounds cardiomyocytes, creating a network for them. *Itgb1*, which encodes the β1, was deleted via *Nkx2.5*$^{Cre/+}$ to generate myocardial-specific *Itgb1* knockout (B1KO) mice. B1KO hearts display an absence of a trabecular zone but a thicker compact zone. The levels of hyaluronic acid and versican, essential for trabecular initiation, were not significantly different between control and B1KO. Instead, fibronectin, a ligand of β1, was absent in the myocardium of B1KO hearts. Furthermore, B1KO cardiomyocytes display a random cellular orientation and fail to undergo perpendicular cell division, be organized properly, and establish the proper tissue architecture to form trabeculae. Mosaic clonal lineage tracing showed that *Itgb1* regulates cardiomyocyte transmural migration and proliferation autonomously.

Conclusion: β1 is asymmetrically localized in the cardiomyocytes, and some of its ECM ligands are enriched along the luminal side of the myocardium, and fibronectin surrounds cardiomyocytes. β1 integrins are required for cardiomyocytes to attach to the ECM network. This engagement provides structural support for cardiomyocytes to maintain shape, undergo perpendicular division, and establish cellular organization. Deletion of *Itgb1* leads to loss of β1 and fibronectin and prevents cardiomyocytes from engaging the ECM network, resulting in failure to establish tissue architecture to form trabeculae.

Funding: This work was supported by National Heart, Lung, and Blood Institute grant 2R01HL121700-06A1 to M.W. American Heart Association 20TPA35490051 grant to M.W.

American Heart Association 19POST34410093 postdoc fellowship to L.M. National Eye Institute P30EY007551 grant to A.R.B.

O_20

Investigating the role of retinoic acid signaling during morphogenesis of the muscular interventricular septum

Tobias H. Bønnelykke[1], Marie Couderc[1], Claudio Cortes[1], Celia Rousset[1], Rachel Sturny[1], Stéphane Zaffran[2], Robert G. Kelly[1]

[1] *Aix-Marseille Université , CNRS UMR 7288, IBDM, Marseille, France*

[2] *Aix-Marseille Université, INSERM, MMG U1251, 13005 Marseille, France*

Background: The mammalian heart is divided into four chambers by septa that arose during vertebrate evolution to isolate the systemic and pulmonary circulation. Cardiac septa are hotspots for congenital heart defects (CHD), and ventricular and atrial septal defects account for 35% and 15% of CHD, respectively. Despite this, how these septa are formed is poorly understood. Cardiac septa arise at the interface between cells derived from the first (FHF) and second (SHF) heart fields. Previous work in the team has shown that retinoic acid (RA) signaling is required to activate the FHF and venous pole progenitor cell regulator TBX5 in the posterior SHF for subsequent atrial septation.

Methods: In order to further investigate the role of RA signaling at the heart field interface we expressed a conditional dominant negative RA receptor (*RARa403*) in the SHF. A range of Cre lines were used together with pharmacological experiments to define the spatiotemporal requirements for RA signal reception in ventricular septum morphogenesis.

Results and Conclusion: Hearts in which RA signal reception is downregulated in the SHF using *Mef2c-AHF-Cre* are characterized by a common arterial trunk and a failure of muscular interventricular septum morphogenesis resulting in a deep interventricular cleft and a bifid ventricular phenotype that emerges by E12.5. The septal phenotype resembles the bifid hearts of marine mammals of the order Sirenia, implicating RA signaling in the evolution of mammalian ventricular morphology. We identified a late role for RA signal reception during septum formation in cardiomyocytes in the septal core derived from the heart field interface, distinct from requirements for outflow tract development. Moreover, bifid hearts have impaired cardiomyocyte maturation and increased extracellular matrix gene expression. Our results support a model by which ventricular septal morphogenesis involves infolding of the compact myocardial wall during chamber ballooning associated with convergence of the right and left ventricular walls through an RA-dependent fusion mechanism.

O_21

Single cell Multiomics and computational inference of cardiopharyngeal differentiation: the role of Tbx1

Olga Lanzetta[1-2-3], Marchesa Bilio[4], Rosa Ferrentino[4], Katharina Jechow[5], Ilaria Aurigemma[6], Sören Lukassen[5], Johannes Liebig[5], Elizabeth Illingworth[6], Christian Conrad[5], Claudia Angelini[3], Antonio Baldini[7]

[1] *Unit of Clinical Immunology and Translational Medicine, IRCCS Ospedale Policlinico San Martino, Genoa, Italy*

[2] *Department of Experimental Medicine (DIMES), University of Genoa, Genoa, Italy*

[3] *Istituto per le Applicazioni del Calcolo, CNR Naples, Italy*

[4] *Institute of Genetics and Biophysics, CNR Naples, Italy*

[5] *Charité–Universitätsmedizin Berlin, corporate member of Freie Universität Berlin, Humboldt-Universität zu Berlin, and Berlin Institute of Health; Center for Digital Health, Berlin, Germany*

[6] *Dept. of Chemistry and Biology, University of Salerno, Italy*

[7] *Dept. of Molecular Medicine and Medical Biotechnology, Univ. Federico II, Naples, Italy*

Background: TBX1 is essential for the development of the pharyngeal apparatus and its gene, Tbx1 serves as a marker for cardiopharyngeal mesoderm (CPM), a multipotent population that provides progenitors for the second heart field and branchiomeric muscles. However, in mammals, the molecular mechanisms underlying the diversification of CPM, and the role of TBX1 therein, remain unclear.

Methods: We used a cell culture model of CPM differentiation of wild-type and $Tbx1^{-/-}$ mouse ES cells, and collected multiomic data (simultaneous single-cell RNA-seq and ATAC-seq) at two stages of differentiation. Selected results were validated using WT and $Tbx1^{-/-}$ mouse embryos. We integrated scRNAseq and scATACseq data using Seurat and constructed gene cluster-specific co-expression modules using the hdWGCNA package. Top hub genes from these modules were used as input to measure the chromatin response to genotype. RNA velocity-based analysis, using the Dynamo package, was used to infer the directionality of cell state transitions. Additionally, we used Monocle3 to infer cellular trajectories.

Results and Conclusion: We found that TBX1 regulates chromatin accessibility and gene expression within an evolutionary conserved transcriptional module crucial for the development of the trunk and pharynx. This module includes genes encoding Tea Shirt (Tshz), Sine Oculis (Six), Eye absent (Eya), and Ebf/Collier factors. Analysis of regulatory regions identified using a machine-learning approach, suggests that TBX1 regulates this module via SIX factors. In addition, we found that while its loss does not cause major trajectory shifts, TBX1 is required to prevent a drift of early progenitors towards inappropriate transcription profiles. Specifically, a subset of mesodermal cells missing TBX1 activates transcription of some epithelial/ectodermal and extra cellular matrix genes. We conclude that loss of TBX1 has broad chromatin consequences and results in incoherent cell fate trajectories within the CPM.

Funding: EU Horizon 2020 No 824110; Telethon Foundation GMR22T1012, and Italy PRIN 2022XFE7M2.

O_22

WT1/ITGA4 EPICARDIAL PROGENITOR CONVERSION INTO EXPANDING EPICARDIAL-DERIVED CELLS: NON-CANONICAL WNT SIGNALLING MEDIATES EARLY PROGENITOR PROLIFERATION

Marín-Sedeño E.[1,2], Cobos-Figueroa J[1,2], Sánchez-Mata A[1,2], Cano, E.[1,2], Díaz-Martínez L. [3], Lescroart F.[4], Ruiz-Villalba A.[1,2]*, Pérez-Pomares J.M.[1,2]*

[1] *Departamento de Biología Animal, Facultad de Ciencias, Universidad de Málaga, Málaga, Spain*

[2] *Instituto Malagueño de Biomedicina (IBIMA)-Plataforma BIONAND, Junta de Andalucía, Universidad de Málaga, Málaga, Spain*

[3] *Centro de Supercomputación y Bioinnovación (SCBI), Universidad de Málaga, Málaga, Spain*

[4] *Aix-Marseille Université, INSERM, MMG U1251, 13005 Marseille, France*

***Correspondence:** Prof. José M. Pérez-Pomares. E-mail: jmperezp@uma.es; Dr. Adrián Ruiz-Villalba. E-mail: adruiz@uma.es

The epicardium is the outermost tissue layer of the heart, and its correct formation from the proepicardium (PE), i.e. the transient extracardiac structure that contains epicardial progenitors, is crucial to proper cardiac morphogenesis. However, our knowledge on the molecular and cellular mechanisms regulating epicardial progenitor cell conversion into primitive epicardial cells and then to mesenchymal epicardial-derived cells (EPDCs) is very limited. By combining single cell transcriptomic analysis, *in vivo* mouse models for genetic/lineage cell tracing, and *in vitro* assays, we have analysed PE cell diversity and characterized true epicardial progenitors. Our study reveals that Wt1High/Itga4High PE cells represent bona fide progenitors of primitive epicardial cells and epicardially-derived mesenchymal cells (EPDCs). Moreover, these cells display an unexpected high proliferative activity that is regulated by the Wnt5a/Ror2/c-Jun axis, revealing a key role for Wnt signaling in the expansion of epicardial-EPDCs. This study provides detailed information into the formation of early EPDCs and lays the foundation for future research on EPDC differentiation.

Keywords: Proepicardium; epicardium; proliferation; cell lineage tracing; single-cell RNA-seq; Wnt signaling pathway.

Funding: National Research Agency (EAI) – Spanish Ministry of Science and Innovation. PID2021-122626-OB-I00

www.decalab.es

O_23

Intramyocardial sprouting tip cells specify coronary arterialization

Dr. Elena Cano[1-5], Jennifer Schwarzkopf[1,2,3], Dr. Masatoshi Kanda[6,7], Dr. Eric L. Lindberg[6,8], Irene Hollfinger[1], Cristina Pogontke[4,5], Caroline Braeuning[9], Dr. Cornelius Fischer[9], Prof. Norbert Hübner[2,3,6], Prof. Holger Gerhardt[1,2,3,10]

[1] *Integrative Vascular Biology Lab, Max-Delbrück Center for Molecular Medicine in the Helmholtz Association (MDC), Berlin, Germany*

[2] *DZHK (German Center for Cardiovascular Research), Berlin, Germany*

[3] *Charité-Universitätsmedizin, Berlin, Germany*

[4] *Department of Animal Biology, University of Málaga, Spain*

[5] *Cardiovascular Development and Disease, Biomedical Research Institute of Malaga and Nanomedicine Platform (IBIMA - BIONAND Platform), Málaga, Spain*

[6] *Cardiovascular and Metabolic Sciences, Max-Delbrück Center for Molecular Medicine in the Helmholtz Association (MDC), Berlin, Germany*

[7] *Department of Rheumatology and Clinical Immunology, Sapporo Medical University, Sapporo, Japan*

[8] *Department of Medicine, Ludwig-Maximiliams-University Munich, Germany*

[9] *BIH/MDC Genomics Technology Platform, Berlin, Germany*

[10] *Berlin Institute of Health (BIH), Berlin, Germany*

The elaborate patterning of coronary arteries critically supports the high metabolic activity of the beating heart. How coronary endothelial cells coordinate hierarchical vascular remodeling and achieve arteriovenous specification remains largely unknown. Single-cell transcriptomics and histological validation were used to delineate heterogeneous transcriptional states of the developing and mature coronary endothelium with a focus on sprouting endothelium and arterial cell specification. Genetic lineage tracing and high-resolution three-dimensional imaging were employed to characterize origin and mechanisms of coronary angiogenic sprouting, as well as to fate-map a selective endothelial lineages. Integration of single-cell transcriptomic data from ischemic adult mouse hearts and human embryonic data served to assess the conservation of transcriptional states across development, disease and species.

We discovered that coronary arteries originate from cells that have previously transitioned through a specific tip cell phenotype. We identified non-overlapping intramyocardial and subepicardial tip cell populations with differential gene expression profiles and regulatory pathways. Esm1-lineage tracing confirmed that intramyocardial tip cells selectively contribute to coronary arteries and endocardial tunnels, but not veins. Notably, pre-arterial cells are detected from development stages to adulthood, increasingly in response to ischemic injury, and in human embryos.

Altogether, our findings suggest that tip-cell-to-artery specification is a conserved mechanism, which drives arterialization of the intramyocardial plexus and endocardial tunnels throughout life and is reactivated upon ischemic injury. Understanding the molecular and cellular cues that pattern coronary arteries is crucial to develop innovative therapeutic strategies that restore functional perfusion within the ischemic heart.

O_24

The role of Slit signaling in chamber-specific cardiomyocyte polyploidization

Sabrina Kaminsky[1], Eva Zickgraf[1], Lorna Wessels[1], Didier Y.R. Stainier[2] and Chi-Chung Wu[1]

[1] *Heidelberg University, Medical Faculty Mannheim, European Center for Angioscience, Mannheim, Germany*
[2] *Max Planck Institute for Heart and Lung Research, Department of Developmental Genetics, Bad Nauheim, Germany*

Background: Polyploidy, the presence of more than two complete sets of chromosomes per cell, is commonly found in mammalian cardiomyocytes (CMs). Mouse CMs become polyploid during the first week after birth due to defective karyokinesis and/or cytokinesis, coinciding with their cell cycle exit and the loss of regenerative potential. While CM polyploidy has been identified as a limiting factor for cardiac regeneration, the regulation of this process, particularly the involvement of extracellular signals, remains largely unexplored.

Methods: We assessed the ploidy of CMs isolated from mouse ventricles and atria at different postnatal stages. To identify differentially expressed genes, we performed transcriptomic analysis. Genes related to Slit signaling (eg., *Slit2*, *Slit3*, *Gpc1*) were enriched in the atria. Analysis of a published dataset revealed that *Slit2*, *3* are higher expressed in mononucleated compared to binucleated CMs at P7. We conducted knockdown (KD) experiments using siRNAs targeting *Slit2*, *3*, and their receptors *Robo1*, *2*, and/or *4*, and *Gpc1* in primary postnatal rat CMs. Rescue experiments were performed using recombinant SLIT2 protein.

Results and Conclusion: Our results showed that CM ploidy varies extensively in different heart chambers across postnatal stages. Consistent with previous reports, most ventricular CMs rapidly became binucleated from postnatal day 4 (P4) to P9, while >70% atrial CMs remained mononuclear diploid until adulthood. KD of *Slit2, 3* increased primary postnatal CM polyploidization that could be rescued by recombinant SLIT2. KD of *Gpc1*, but not of the Robo family, resulted in a binucleation phenotype comparable with *Slit2, 3* KD. Mechanistically, *Slit2* and *3* loss-of-function led to the downregulation of *Cep55*, a centrosomal protein required for cytokinesis, whose KD significantly increased binucleation in primary CMs. Altogether, our results suggest that Slit signaling, functioning through GPC1, is required for CM cytokinesis and may underlie, at least in part, the chamber-specific differences in CM ploidy.

Funding: Supported by Health + Life Science Alliance Heidelberg Mannheim, CRC1366 and the Emmy Noether Programm

Lab website: ccwulab.com

O_25

Functional architecture of cardiac TF regulatory landscapes in control of mammalian heart development

Virginia Roland[1], Matteo Zoia[1], Johannes Tüchler[2], Ekapaksi Wisnumurti[1], Andrea Esposito[3], Mattia Conte[3], Raquel Rouco Garcia[4], Julie Gamart[1,5], Virginie Tissières[1,5], Prateek Arora[6], Vincent Rapp[1], Ines Marques[6], Gretel Nusspaumer[7], Guillaume Andrey[4], Javier Lopez-Rios[7], Nadia Mercader[6], Mario Nicodemi[3], Iros Barozzi[2] and Marco Osterwalder[1,5]

[1] *Department for BioMedical Research (DBMR), University of Bern, Bern, Switzerland*
[2] *Center for Cancer Research, Medical University of Vienna, Vienna, Austria*
[3] *Department of Physics, University of Naples Federico II and National Institute of Nuclear Physics, Naples, Italy*
[4] *Department of Genetic Medicine and Development and iGE3, Faculty of Medicine, University of Geneva, Geneva, Switzerland*
[5] *Department of Cardiology, Bern University Hospital, Bern, Switzerland*
[6] *Institute of Anatomy, University of Bern, Bern, Switzerland*
[7] *Centro Andaluz de Biología del Desarrollo (CABD), CSIC-Universidad Pablo de Olavide Junta de Andalucía, Seville, Spain*

Background: Congenital heart disease (CHD) is the most common birth defect in humans and while the majority of associated variants map to non-coding genomic regions, the functional *cis*-regulatory architecture underlying development of the four-chambered mammalian heart remains insufficiently characterized. The HAND2 and GATA4 transcription factors (TFs) are CHD-associated key regulators in second heart field progenitors and their inactivation leads to severe cardiac abnormalities, including outflow tract, ventricular and valve defects.

Methods: Here, we used a combination of genome editing, single cell multiome profiling, region capture Hi-C and site-directed enhancer-reporter transgenesis in mouse embryonic hearts to functionally characterize *Hand2* and *Gata4* enhancer landscapes at the cell type level.

Results and Conclusion: Genomic deletion of previously identified heart enhancers in *Gata4* and *Hand2* loci revealed a significant degree of transcriptional resilience, suggesting that redundant enhancer contributions are common in cardiac TF regulation. Concordantly, a subset of these enhancers was required for embryonic viability and entailed cardiac defects in a dosage sensitized background. To facilitate prediction of novel heart enhancers across cardiac cell types, we performed single-cell Multiome profiling during cardiac chamber expansion, which enabled genome-wide correlation of enhancer signatures with target gene activity and the discovery of cell state transitions. In combination with chromatin capture, our resource allowed accurate identification of a novel set of far-upstream *Hand2* heart enhancers located in a chromatin domain with cardiac-specific topology and cumulatively required for endocardial *Hand2* expression, ventricular trabeculation and patterning of endocardial cushions. Our results reveal the cell type signatures of cardiac *cis*-regulatory elements during development and demonstrate critical functional contributions of conserved TF heart enhancers, important for cardiac morphogenesis and interpretation of CHD-associated genomic variants.

Funding source: Swiss National Science Foundation (SNSF, #PCEFP3_186993)

O_26

CHD7 modulates Second Heart Field gene expression via binding to novel distal cardiac enhancers.

Nancy Stathopoulou[1, 2], Neil Slaven[3], Axel Visel[3], Len A. Pennacchio[3], Deyou Zheng[4], Peter Scambler[2]

[1] *Institute of Developmental and Regenerative Medicine, University of Oxford, Oxford , UK*
[2] *Great Ormond Street Institute of Child Health, University College London, London, UK*
[3] *Functional Genomics Department, Lawrence Berkeley National Laboratory, Berkeley, USA*
[4] *Albert Einstein College of Medicine, New York, USA*

Background: CHARGE syndrome is a complex birth defect associated with deletion or mutations of the chromatin remodeller chromodomain helicase DNA-binding 7 (CHD7). Mouse models of *Chd7* haploinsufficiency have severe cardiovascular defects, and conditional inactivation of *Chd7* in the cardiopharyngeal mesoderm (CPM) recapitulates the cardiac phenotype of CHARGE patients.

Material and methods: We generated cKO of *Chd7* in CPM and studied cardiac progenitors using transcriptomic and epigenomic analyses, *in vivo* expression and bioinformatics analysis. Novel possible enhancers are tested using the CRISPR/Cas9-mediated site-specific transgenic mouse assay enSERT.

Results and Conclusion: We investigated the role of CHD7 in the CPM and discovered genes and pathways affected in E9.5 mutants. We found the balance of anterior and posterior second heart field (a/pSHF) progenitors disturbed, with expression of pSHF markers increased and aSHF and early cardiomyocyte markers reduced in mutants. Genome-wide profiling of CHD7 binding in cardiac progenitors, combined with transcriptomics from cKO embryos identified direct CHD7 targets during the early stages of cardiac commitment and differentiation. Our analysis shows that CHD7 binds distal elements, with predicted enhancer activity, regulating FHF and SHF gene networks. Moreover, CHD7 shares a subset of its target sites and physically interacts with ISL1.

We are now characterizing selected CHD7-bound putative enhancer elements, located >100kb from key cardiac TFs such as *Isl1* and *Hand1*, using the enSERT assay. Preliminary data shows that 90% of the elements we tested have enhancer activity. We are now characterizing the function of selected candidates during the early stages of cardiac development.

Funding sources: NS is currently supported by an IDRM Transition Fellowship (by the Department of Paediatrics, University of Oxford). Work at University College London was supported by the British Heart Foundation and the Leducq Foundation.

https://www.idrm.ox.ac.uk/research/cardiology/cardiology-research-groups/stathopoulou-group

O_27

Investigating epicardial-myocardial interactions in the ventricle during cardiac morphogenesis

Radha Kulkarni, Thomas Juan, Stefan Günther, Didier Stainier

Max Planck Institute for Heart and Lung Research, Bad Nauheim, Germany

Background: The epicardium adheres to the myocardium during early cardiac development and covers the entire ventricular surface over time. Upon attachment, epicardial cells not only serve as a multipotent progenitor source for non-myocardial cell fates, but also signal the myocardium via paracrine molecules to facilitate cardiomyocyte proliferation, growth, and maturation. Nevertheless, a detailed *in vivo* analysis of cell-cell interactions between the epicardium and myocardium and the underlying pathways is lacking.

Methods: We performed single-cell RNA sequencing on WT hearts to understand the transcriptomic landscape during epicardial attachment. We used zebrafish *wt1a*$^{-/-}$ larval hearts, whose ventricle is devoid of epicardial coverage, to investigate the effects on myocardial development through live confocal microscopy. We also performed bulk RNA sequencing on *wt1a*$^{-/-}$ and WT whole hearts to uncover genes important for epicardial-myocardial interactions. To study genes of interest, we generated loss-of-function tools using CRISPR-Cas9 mediated genome editing.

Results and Conclusion: Upon imaging *wt1a*$^{-/-}$ larval hearts, we observed that epicardial attachment plays a role in establishing cardiomyocyte cell polarity and trabecular development. Interactome analysis revealed candidate genes responsible for establishing cell polarity such as *jam2b (junctional adhesion molecule 2b)*, which is specifically expressed in the attaching epicardial cells. Jam2b interacts with Jam3b to mediate cell junction assembly, and *jam3b* is highly enriched in ventricular cardiomyocytes. We hypothesize that Jam family proteins, in association with other junctional molecules, maintain cardiomyocyte polarity and facilitate ventricular morphogenesis.

Funding: Max Planck Society

O_28

Maternal valproic acid exposure perturbs neural crest cell migration in mice

Duncan B. Sparrow[1], Victoria S. Rashbrook[1], Laura E. Bell[1], Jacinta I. Kalisch-Smith[1], Selina Tsai[1], Paul Young[2], David Humphries[2], Eleni Giannoulatou[2]

[1] *Department of Physiology, Anatomy and Genetics, University of Oxford, UK*
[2] *Victor Chang Cardiac Research Institute, Sydney, Australia*

Background: Congenital heart disease (CHD) is the most common human birth defect. It affects 1.7% of live births worldwide, and is the leading non-communicable cause of infant mortality. 30% of CHD cases can be attributed solely to genetic causes, but the causes of the other 70% are less clear. Some of these cases arise from *in utero* exposure to environmental teratogens in early pregnancy. One such teratogen is the anti-epileptic drug valproic acid (VPA), which is known to cause specific heart and craniofacial defects. However, there are conflicting hypotheses of the molecular mechanism by which VPA perturbs embryonic development.

Methods: Pregnant C57BL/6J mice were injected with 500 mg/kg VPA on E7.5 or E8.5. Embryos were collected at E15.5 for morphological analysis by High-Resolution Episcopic Microscopy and 3D modelling. Single-cell transcriptomics and epigenomics were performed on E8.5 embryos, and results validated by RNAscope and immunohistochemistry.

Results and Conclusion: Mouse embryos exposed to VPA at E7.5 (but not E8.5) have highly penetrant heart and neural tube defects. Single-cell transcriptomic analysis detected significantly reduced numbers of migrating neural crest cells in E7.5 VPA-exposed embryos at E8.5. This was not due to reduced proliferation or induction of apoptosis in pre-migratory neural crest cells. Gene and protein expression analysis suggested that VPA exposure induces excess canonical Wnt signalling and a metabolic shift in pre-migratory neural crest cells, causing delayed delamination of these cells from the neural tube. We are currently analysing single-cell epigenomic data to determine how VPA exposure causes these effects. Our results may lead to the development of preventive approaches or the identification of novel therapeutic targets to reduce the incidence of CHD in humans.

Funding: Supported by British Heart Foundation Senior Basic Science Research Fellowship FS/SBSRF/22/31022 and Additional Ventures Single Ventricle Research Fund 2021.

Hyperlink: https://www.dpag.ox.ac.uk/research/sparrow-group

O_29

Left-right differences in Wnt inhibition underlie a pro-fibrotic microenvironment and atrial fibrillation predisposition for *Pitx2* deficiency

Jeffrey D. Steimle [1,†], Zachary A. Kadow [1,†], Matthew C. Hill [1], Xiao Li [2], Ge Tao [1], and James F. Martin [1,3,4]

[1] *Department of Integrative Physiology, Baylor College of Medicine, Houston, TX, USA*
[2] *McGill Gene Editing Laboratory, Texas Heart Institute, Houston, TX, USA*
[3] *Cardiomyocyte Renewal Laboratory, Texas Heart Institute, Houston, TX, USA*
[4] *Center for Organ Repair and Renewal, Baylor College of Medicine, Houston, TX, USA*

†. Authors contributed equally to this work.

Atrial fibrillation (AF) is the most common sustained cardiac arrhythmia, primarily occurring with advanced age, and is associated with an increased risk of stroke and heart failure. Common variation in the non-coding region of *PITX2*, the left-sided determining transcription factor, is the strongest genetic signature of AF risk and further enhances age-associated risk. As risk alleles are associated with decreased expression of *PITX2* in the left atria (LA), we utilized our well-characterized *Pitx2* null mouse allele to study the effect of advanced age at the single-cell level. We performed single nuclear RNA-sequencing (snRNA-seq) on the LA of 24-month-old heterozygous and littermate controls and compared with our previously reported 6-month-old datasets. We identified a gene signature of decreased Wnt-signaling inhibition from *Pitx2*-deficient cardiomyocytes and increased Wnt-sociated activation of fibroblasts. Transgenic Wnt reporter mice demonstrate that *Pitx2* deficiency increases LA Wnt-signaling to right atrial levels. Histological analysis of aged *Pitx2*-deficient mice had increased LA fibroblast proliferation and interstitial fibrosis not previously seen in young animals. Furthermore, aged, *Pitx2*-deficient mice were susceptible to atrial arrhythmogenesis when challenged by transesophageal pacing. Lastly, we demonstrate that pharmacological Wnt inhibition is sufficient to reduce fibroblast activation and proliferation in the *Pitx2*-deficient LA. Collectively, these data indicate that reduced LA *Pitx2* leads to increased left-sided Wnt signaling, resulting in LA remodeling and increased arrhythmia susceptibility with age. Furthermore, in functionally validating the *Pitx2*-Wnt-fibrosis signaling axis in the aging LA, our data provides new insights for both AF pathogenesis and therapeutic strategies.

O_30

Constitutive overexpression of the voltage-gated sodium channel Scn5lab in atrial cardiomyocytes leads to arrhythmia and induces fibrosis

Marco Tarasco[1], Pia R. Lundegaard[2], Didier Y. R. Stainier[1]

[1] *Max Planck Institute for Heart and Lung Research, Bad Nauheim, Germany*

[2] *Department of Biomedical Sciences, Faculty of Health and Medical Sciences, University of Copenhagen, Denmark*

Background: Cardiac arrhythmias are associated with increased risk of stroke, heart failure, and sudden death. Atrial fibrillation (AF) is normally caused by abnormal electrical impulses within atrial cardiomyocytes that lead to a fast and irregular heart rhythm. Despite its clinical importance, the mechanisms underlying AF onset and progression remain poorly understood.

Methods: We have generated a zebrafish line that continuously overexpresses the voltage-gated sodium channel gene *scn5lab* in atrial cardiomyocytes. By taking advantage of confocal microscopy, echocardiography, µCT, electrocardiogram analysis, and histological techniques, we have assessed morphological and functional changes in the transgenic hearts.

Results and Conclusion: We found that continuous overexpression of *scn5lab* causes an increased heart rate from 72 hours post fertilization up to 21 days post fertilization (dpf). During embryonic and larval development, the transgenic animals display impaired cardiac function, characterized by reduced fractional shortening, disrupted calcium handling, and increased retrograde blood flow in the atrioventricular canal (AVC). Furthermore, the atrium is enlarged, which is likely a consequence of extracellular matrix (ECM) remodeling, as suggested by increased ECM thickness. During juvenile/adult stages, echocardiography revealed a further decline in heart function, characterized by reduced ejection fraction, and increased retrograde blood flow in the AVC. Additionally, electrocardiography revealed the absence or shortening of P-waves. Histological staining revealed increased collagen deposition within the atrial tissue by 2 months of age. Collagen deposition within the myocardium was further confirmed at the subcellular level by electron microscopy. Bulk RNA sequencing at 14 dpf revealed the dysregulation of ECM related genes.

In summary, we have established a zebrafish model of atrial cardiomyopathy that resembles many of the phenotypes observed in AF patients, allowing us to further investigate the cellular and molecular mechanisms involved in this disease. We are particularly interested in understanding how increasing the heart rate can lead to fibrosis.

O_31

Tmem161b is required in for the maintenance of mammalian cardiac rhythm and interacts with key regulators of intracellular Ca^{2+} handling

Jessica F Briffa, Nicole Dominado, Benjamin L Parker & Kelly A Smith

Department of Anatomy and Physiology, The University of Melbourne, Parkville, VIC 3010, Australia

Background: Mutation of t*mem161b* in zebrafish causes skipped beats and bradycardia. Electrophysiological analyses suggest this is associated with increased intracellular cytosolic Ca^{2+} however very little else is known about its function. In this study, we sought to uncover 1) Whether Tmem161b is functionally conserved in mammals, and 2) how Tmem161b functions at a molecular level.

Methods: Global knockout of *Tmem161b* in mice results in perinatal lethality, preventing us from assessing embryonic heart function. We therefore generated a conditional Tmem161b KO (cKO) mouse, creating a Tmem161b floxed allele and crossing it with the tamoxifen-inducible ubiquitous Cre line, Ubc-Cre^{ERT2}. We deleted Tmem161b at 3 months of age and performed conscious ECGs, behavioural recordings, and body composition analyses. In parallel, pulldown experiments were performed to investigate what Tmem161b protein physically interacts with. Transgenic zebrafish expressing Tmem161b-Citrine fusion proteins were used: adult zebrafish hearts were dissected, and pulldowns performed. Tmem161b-Citrine binding partners were identified using LC-MS/MS.

Results and Conclusion: cKO Tmem161b mice rapidly lost body weight (predominantly fat mass), preventing analyses later than 5 weeks post-tamoxifen. At 1-week post-tamoxifen, cardiac electrical activity was unchanged. By 5-weeks, the heart rate of cKO Tmem161b mice was reduced, the QTcH duration increased, and heart rate variability increased. We next examined Tmem161b interacting proteins via pulldowns. 54 Tmem161b-Citrine binding partners were identified, and this included the Ca^{2+} SR channels, Ryr2 and Serca2. This interaction suggests Tmem161b may directly modulate intracellular Ca^{2+} handling via these Ca^{2+} channels. To further validate this, we are examining total and phosphorylated Serca2, Ryr2, and PLB protein levels, although these experiments are ongoing.

Overall, this study demonstrates that Tmem161b interacts with key molecules involved in intracellular Ca^{2+} handling and that without Tmem161b, a progressive degeneration in cardiac function occurs, consistent with observations in zebrafish.

Funding: This work is supported by funding from the Australian National Health & Medical Research Council (NHMRC), award #2004460.

https://biomedicalsciences.unimelb.edu.au/sbs-research-groups/anatomy-and-physiology-research/stem-cell-and-developmental-biology/Smith-Lab-Cardiac-Genetics

O_32

The nutrient sensor CRTC and Sarcalumenin/Thinman represent a new pathway in cardiac hypertrophy

Cristiana Dondi[1], Georg Vogler[1], Alex Colas[1], Rolf Bodmer[1], Marc Montminy[2], Karen Ocorr[1]

[1] *Development, Aging and Regeneration Program Center for Genetic Disorders and Aging Research Sanford Burnham Prebys Medical Discovery Institute 10901 North Torrey Pines Road, La Jolla, CA 92037*

[2] *Clayton Foundation Laboratories for Peptide Biology Salk Institute for Biological Studies 10010 North Torrey Pines Road, La Jolla, CA, 92037*

Introduction: Metabolic disorders such as obesity, insulin resistance and diabetes increase the risk of heart disease, especially cardiac hypertrophy. At the cellular level, the phosphatase calcineurin (CaN) has been shown to mediate cardiomyocyte hypertrophy via activation of NFAT. In the liver, CaN activates the nutrient sensor CRTC, however cardiac roles for CRTC have not been identified.

Methods: The gene networks regulating cardiac structure and function are highly conserved between flies, fish, and humans. We used high-throughput optical assays to quantify cardiomyocyte function in *Drosophila, zebrafish,* and induced Cardiomyocytes models (hIPSC-CMs) in response to CRTC knockdown (KD) to investigate a role for CRTC in cardiac hypertrophy. We used immunohistochemistry to show cardiac structural changes in both flies and fish. RNAseq of isolated fly hearts identified a paralog of *sarcalumenin/thinman (tnmn)* as a novel downstream target of CRTC.

Results: *CRTC* is conserved in both *Drosophila* and zebrafish. In the fly, null mutants exhibit reduced fat stores, severe cardiac restriction, myofibrillar disorganization, cardiac fibrosis, and tachycardia, all hallmarks of heart disease. Cardiac-specific knockdown (KD) of *CRTC* mimicked the cardiac restriction and other heart defects of *CRTC* null mutants. Notably, cardiac-overexpression (OE) of *CRTC* caused hypertrophy with upregulation of specific hypertrophy markers, including myosin heavy chain. CaN OE also induced hypertrophy that was partially blocked in *CRTC* mutants, suggesting that CRTC mediates the effects of CaN. CRTC3 is also expressed in zebrafish hearts and morpholino KD caused cardiac restriction, as in flies. RNAseq of both *CRTC* KD and OE hearts revealed contra-regulated genes involved in glucose, fatty acid, and amino acid metabolism suggesting that CRTC acts as a metabolic switch in the heart. We also identified the fly ortholog of *Sarcalumenin (Srl) (sarcalumenin/thinman),* a Ca^{2+}-binding protein of the sarcoplasmic reticulum, as a likely downstream target. Cardiac KD and OE of this gene recapitulated the restrictive and hypertrophic phenotypes of *CRTC* KD and OE. CRTC 1, 2 & 3 are also expressed in human iPSC-derived cardiomyocytes (hiPSC-CMs) and KD of CRTC 2&3 increased action potential duration (APD), supporting a fundamental role of CRTC in heart function. As in flies, *CRTC* KD in hiPSC-CMs reduced *Srl* expression and increased APD. Our data from three model systems suggest that *CRTC* signaling plays a conserved and cardiac-autonomous role in maintaining heart structure and function, in part by regulating *Srl* expression which in turn modulates cytosolic Ca^{2+} levels. Thus, CaN - CRTC - SRL signaling likely represents a new and conserved pathway mediating cardiac hypertrophy and may also play more general roles in muscle cell maintenance**.**

Funding: NIH R01 HL132241

Hyperlink: https://www.cell.com/cell-reports/fulltext/S2211-1247(24)00878-7

O_33

Investigating a Novel Genetic Cause of Cardiomyopathy and Therapeutic Interventions

Millie O Fullerton, Simon D Bamforth, Helen M Phillips

Bioscience Institute, Faculty of Medical Sciences, Newcastle University

Background: A homozygous pathogenic variant, p.Arg253Trp *SLC5A6*, has been identified in a family whereby two siblings presented with dilated cardiomyopathy. *SLC5A6* encodes the Sodium-dependent Multivitamin Transporter, a transmembrane protein that is crucial for facilitating the active transport of three vitamins: biotin, pantothenic acid and lipoic acid; all of which are critical, organic enzyme cofactors required for energy metabolism in the mitochondria. Energy metabolism is a fundamental process in cardiac maintenance and, as the most metabolically demanding organ in the body, the heart requires a high ATP turnover to achieve efficient contractile function of the myocardium.

Methods: *Slc5a6* cardiac-specific knockout (*Slc5a6*^{cKO}) mouse models have been employed to investigate *Slc5a6 in vivo* and immunofluorescent, proteomics and electron microscopy techniques have been performed to investigate its role in mitochondrial function in the heart. Vitamin supplementation was administered to *Slc5a6*^{cKO} mice and electrocardiograph and magnetic resonance imaging was used to assess the effect of treatment on cardiac conduction and function.

Results and conclusion: Conditional deletion of *Slc5a6* in cardiomyocytes results in the development of cardiomyopathy and abnormal electrocardiogram parameters were detected from 6 weeks, followed by sudden death at 20 weeks old. *Slc5a6*^{cKO} hearts were significantly fibrosed and pathways associated with mitochondrial energy metabolism, particularly fatty acid-beta oxidation, were significantly downregulated. EM also showed abnormal mitochondrial morphology in the hearts of *Slc5a6*^{cKO} mice. It has been shown that vitamin supplementation to *Slc5a6*^{cKO} mice prolonged life span and improved both cardiac conduction and function. Mitochondrial dysfunction was restored in vitamin supplemented *Slc5a6*^{cKO} mice therefore, this project not only aims to establish the role of *Slc5a6* in the heart, but to investigate whether vitamin supplementation can prevent mitochondrial defects and delay progression to cardiomyopathy.

Funding: Research was supported by the Barbour Foundation and the British Heart Foundation.

O_34

Exploring the Role of PRDM16 in MYBPC3-Related Hypertrophic Cardiomyopathy and Left Ventricular Non-Compaction in Mouse Models

Alba Pau-Navalón[1,2], Alejandro Salguero-Jiménez[1,2], Marcos Siguero-Alvarez[1,2], María Sabater Molina[2,3], Bin Zhou[4], Jorge Alegre-Cebollada[5], Donal MacGrogan[1,2], Juan R. Gimeno-Blanes[2,3] & José Luis de la Pompa[1,2]

[1] *Intercellular Signaling in Cardiovascular Development & Disease Laboratory, Centro Nacional de Investigaciones Cardiovasculares Carlos III (CNIC), 28029 Madrid, Spain*

[2] *CIBER CV, 28029 Madrid, Spain*

[3] *H.U. Virgen de la Arrixaca, Murcia and Universidad de Murcia, Spain*

[4] *State Key Laboratory of Cell Biology, Shangai Institute of Biochemistry and Cell Biology, University of Chinese Academy of Sciences, Shangai, China*

[5] *Molecular Mechanics of the Cardiovascular System Laboratory, Centro Nacional de Investigaciones Cardiovasculares Carlos III (CNIC), 28029 Madrid, Spain*

Background: Familial cardiomyopathies are genetically complex diseases, with pedigrees that may contain individuals with features of different cardiomyopathies. Hypertrophic cardiomyopathy (HCM) and Left ventricular Non-Compaction (LVNC) exemplify this complexity, often co-occurring likely due to shared genetic factors. Mutations in *MYBPC3*, encoding Myosin binding protein C, are found in families with mixed LVNC and HCM phenotypes.

Methods: Using Crispr-Cas9 technology, we generated mouse models carrying three different *MYBPC3* variants identified in pedigrees containing individuals with HCM and LVNC. *Prdm16* conditional loss- and gain-of-function model were generated by Crispr-Cas9 gene editing and gene targeting in mESC. Transcriptomic profile was analyzed by RNA-seq. Lineage tracing analysis and PRDM16 expression were studied by immunofluorescence.

Results and Conclusion: We found that homozygous mutant mice harboring the *Mybpc3* nonsense variants developed a transient fetal hypertrabeculation phenotype that evolves in postnatal hypertrophy. In order to identify the molecular mechanisms driving this phenotypic conversion, we have transcriptionally characterized mutant hearts at postnatal day 1 (P1) and P7. Gene expression profiling shows a dynamic change in *Prdm16* transcription, a transcription factor involved in establishing compact myocardium identity, that is upregulated at P1 and downregulated at P7. Lineage analysis using the $Hey2^{CreERT2}$ driver reveals an expansion of the compact myocardium towards the trabecular myocardium at E16.5, E18.5 and P1, in parallel with PRDM16 expansion towards the trabeculae and increased proliferation of trabecular cardiomyocytes. We have made similar observations in mice harboring a missense *Mybpc3* variant, suggesting common disease mechanisms underlying loss- and missense Mybpc3 mutations. We believe that establishing the role of PRDM16 in hypertrabeculation, LVNC and HCM will contribute to understanding disease etiology and the design of potential therapeutic treatments.

Funding: FPI fellowship PRE2022-102314 (Spanish Ministry of Science, Universities and Innovation, MCIU) to APN. Grants: PID2022-104776RB-100 and CB16/11/00399 to JLP.

POSTER SESSION ABSTRACTS

Poster Session 1 – Mechanisms of Congenital Heart Disease

Journal of
Cardiovascular Development and Disease
an Open Access Journal by MDPI

Impact Factor: 2.4 (2023);
Journal Rank: JCR - Q2 (*Cardiac and Cardiovascular Systems*)

https://www.mdpi.com/journal/jcdd

Aims
The Journal of Cardiovascular Development and Disease is an international, peer-reviewed, open access journal, which publishes reviews, research papers and communications on cardiovascular development as well as congenital and acquired heart disease. Scientists are encouraged to publish their experimental, theoretical, and descriptive studies and observations in as much detail as possible. There is no restriction on the maximum length of the papers.

Scope
- Gene regulation
- Morphology
- Cardiovascular anatomy
- Cardiac physiology
- Vascular biology
- Gene patterns
- Imaging techniques
- Cardiac regeneration
- Case studies
- Congenital heart disease
- Conduction system and arrhythmias
- Animal models for cardiovascular diseases
- Biophysics
- Genetics

P_01

Epicardial Slit-Robo signalling regulates normal heart development

Tiago P. Dias[1], Carolina Roque Silva[1], Jacinta Kalisch-Smith[2], Duncan Sparrow[2], Andia Redpath[2], Mathilda T. M. Mommersteeg[2], Joaquim Nunes Vieira[1]

[1] *School of Cardiovascular and Metabolic Medicine & Sciences, King's College London, London, United Kingdom*
[2] *Institute of Developmental & Regenerative Medicine - Department of Physiology, Anatomy & Genetics, University of Oxford, Oxford, United Kingdom*

Background: The epicardium is essential for proper development of the heart. From mid-gestation onwards, epicardial cells undergo regionalised waves of epithelial-to-mesenchymal transition (EMT) in response to paracrine signals (e.g. TGFβ), originating epicardium-derived cells (EPDCs). Importantly, EPDCs have been shown to support the development of a thick compact myocardial layer, coronary vessel growth, formation of the atrioventricular (AV) annulus fibrosus and parietal leaflets of AV valves through the secretion of growth factors/chemokines and/or cell differentiation into mural cells (e.g. coronary smooth muscle) and fibroblasts. Nonetheless, the molecular mechanisms guiding epicardial EMT and EPDC function remain poorly characterised. The Slit-Robo pathway, recognised by its role in axon guidance, has been linked to epicardial EMT-associated processes (e.g. AV valve development), with Slit ligands and Robo receptors showing complementary expression patterns in the developing epicardium and underlying tissue. Thus, we hypothesised that epicardial Slit-Robo signalling plays a role regulating EMT and setting up the subepicardial niche during normal heart development.

Methods: *Slit2* or *Slit3* were deleted in the epicardium by crossing the $Wt1^{CreERT2/+}$ driver with $Slit2^{flox}$ or $Slit3^{flox}$ mice and tamoxifen-pulsing at E9.5 and E10.5. Embryonic hearts were harvest and characterized using high-resolution episcopic microscopy (HREM), immunostaining confocal microscopy, and scRNA-seq.

Results and Discussion: Analysis of scRNA-seq data from mouse specimens covering different developmental stages confirmed expression of members of the Slit-Robo family in the forming heart, and the early epicardial expression of *Slit2* and *Slit3* at E10.5. At E13.5, both *Slit2* and *Slit3* were highly detected in epicardial and EPDC clusters, with *Slit2* also detected in a cluster of proliferative EPDCs. At E17.5, *Slit3* expression was still detected in the epicardial cluster, whereas *Slit2* transcripts were present in EPDC-derived mesenchyme. Ongoing characterisation of epicardial-*Slit2* KO revealed abnormal growth and patterning of the cardiac vasculature, lymphatics, and nerves in the subepicardium, whereas epicardial-*Slit3* mutants exhibited thinner ventricular myocardium, with trabeculation extending into the epicardial layer, and reduced expression of WT1, the master regulator of epicardial EMT.

Conclusion: Our preliminary data indicates a requirement for epicardial *Slit2* and *Slit3* in heart development. Future studies will aim to gain mechanistic insight into how Slit-Robo regulates epicardial EMT.

Funding sources: The British Heart Foundation and the Biotechnology and Biological Sciences Research Council.

P_02

Androgen receptor-adrenomedullin axis controls cardiac pacing via proepicardium development

Max Duong Phu[1], Sabrina Matysik[2], Martina Burzcyk[2], Ashraf Al Madhoun[3], Ilona S Skerjanc[4], Martin Burkhalter[1], Melanie Philipp[1]

[1] *Department of Experimental and Clinical Pharmacology and Pharmacogenomics, Section of Pharmacogenomics, Eberhard-Karls-University Tübingen, Tübingen, Germany*

[2] *Institute of Biochemistry and Molecular Biology, Ulm University, Ulm, Germany*

[3] *Genetics and Bioinformatics, Dasman Diabetes Institute, Dasman 15462, Kuwait*

[4] *Department of Biochemistry, Microbiology, and Immunology, University of Ottawa, Ottawa, Ontario, Canada*

Background: Congenital heart disease (CHD) is the most common type of congenital disorder. In patients with CHD arrhythmogenic complications such as sinus node dysfunction, atrioventricular block or complete heart block occur more often than in the normal population. Our knowledge regarding the development of such CHD-linked arrhythmia remains scarce. Hence, additional studies are needed to understand the complex network of molecular and cellular causes underlying CHDs better.

Material & methods: We used zebrafish embryos to conduct a small chemical compound for the identification of novel key factors in vertebrate heart development. Findings were corroborated using different loss- and gain-of-function methods including knockdown, CRISPR-mediated gene editing as well as pharmacological intervention.

Results and Conclusion: We identified the androgen receptor (AR) as a protein modulating early heart development. In the absence of AR function, we observed functional heart defects such as bradyarrhythmia, AV-block and inflow tract edema. Morphological analyses revealed changes in the conduction system, when androgen receptor function was impaired. This was accompanied by a complete loss of proepicardial markers. Overexpression of Bmp2b partially rescued proepicardial gene expression as well as cardiac function. We believe that this impact on the proepicardium is mediated by adrenomedullin, which we found upregulated in embryos devoid of normal androgen receptor function. Consistent with this, loss of adrenomedullin improved cardiac function and morphology.

We hence propose an androgen receptor-adrenomedullin-axis controlling cardiac pacing during vertebrate development.

P_03

Mechanistic Insight Into the Impact of Maternal Obesity on Offspring Cardiovascular Development and Disease

Ashleigh McMullan[1*], James Zwierzynski[2*] Nina Jain[1], Laura Haneline[1], Weinian Shou[1], Kok Lim Kua[1,3], Swetansu Hota[1], Matthew Durbin[1]

[1] *Department of Pediatrics, Herman B Wells Center for Pediatric Research, Indiana University School of Medicine, Indianapolis, IN, 46202, USA*

[2] *Department of Biology, Stanford University, Stanford, CA 94305, USA*

[3] *Center for Diabetes and Metabolic Disease Research, Indiana University School of Medicine, Indianapolis, IN 46202, USA*

*Contributed equally

Background: Obesity is a major public health concern, with rates rising globally, impacting a substantial proportion of women of reproductive age. Maternal obesity is increasingly recognized as a significant risk factor for the development of CHD in the fetus. Despite the established association between maternal obesity and increased CHD risk in offspring, the mechanism remains unknown.

Methods: Therefore, we employed a diet-induced maternal obesity murine model and performed analysis on developing embryo hearts to elucidate the mechanism of CHD pathology due to maternal obesity. We performed a complementary analysis of the transcriptome, using both single-nuclei (snRNAseq) and bulk RNA sequencing (RNA-seq), as well as both global and phospho-enriched proteome analysis using tandem-mass tag mass spectroscopy (TMT-MS) and histologic analysis.

Results and Conclusion: The disrupted cardiac transcriptome and proteome demonstrated substantial overlap in cardiac disease-related signaling pathways. Analysis revealed mechanistic insight, including altered oxidative phosphorylation and reactive oxygen species formation, reduced antioxidant capacity, with downregulated *Sod1*, *Gp4x*, and *Hif1a* signaling, resulting in oxidative stress, cell death, and altered rho kinase and actin cytoskeleton signaling. Histologic analysis confirmed that maternal obesity increased murine embryo cardiac defects. These data offer insight into the mechanisms by which maternal obesity disrupts embryonic cardiac development.

Funding: This study was supported by a National Institutes of Health (Bethesda, MD) 5K08HL148508 Award (MDD), P01HL345599 (MDD and WS) Award, 5K08 HD109636 (KLK), March of Dimes Basil O’Connor Award (KLK) and the Riley Children’s Foundation.

P_04

Paxillin is crucial for thymus and parathyroids development by regulating the architecture of the third pharyngeal pouch endoderm

Iacolare O.[1], Bilio M.[1], Altomonte A.[1], Turner C.[2], Baldini A.[3], Alfano D.[1]

[1] *CNR-Institute of Genetics and Biophysics Adriano Buzzati-Traverso, Via Pietro Castellino, Naples, Italy*
[2] *Department of Cell and Developmental Biology, State University of New York Upstate Medical University, Syracuse, NY, USA*
[3] *Department of Molecular Medicine and Medical Biotechnology, University of Naples Federico II, Naples, Italy*

Background: The Paxillin protein is a key component of focal adhesions and it is encoded by *Pxn,* which is a target of *Tbx1* gene, encoding for a crucial transcription factor required in pharyngeal apparatus development, which is haploinsufficient in DiGeorge syndrome, a textbook-example of a pharyngeal apparatus disorder.

Methods: In this study, using loss-of-function genetics in mice, we investigated whether the Pxn gene has a role in the morphogenesis of the pharyngeal apparatus and whether it interacts with the Tbx1 gene, which encodes a key transcription factor required in pharyngeal development.

Results and Conclusion: Conditional deletion of Pxn in the Tbx1 expression domain did not cause cardiac defects. Instead, the germline Pxn mutation led to cardiac anomalies and to morphogenetic defects of the third pharyngeal pouch (3PP). We found that in Pxn deleted embryos, the 3PP was hypoplastic, lacked the expression of Gcm2, a gene that marks the parathyroid domain, but expressed FoxN1, a gene marking the thymic domain. Consistently, the parathyroids did not develop, and the thymus was hypoplastic and/or malpositioned. The reduced dosage of Tbx1 had a pejorative effect on cardio-pharyngeal defects of Pxn-/- embryos, suggesting an interaction between Pxn and Tbx1. Thus, a novel function of Pxn in

the control of parathyroid and thymic development has been discovered, probably through a function in the pouch endoderm, and intriguingly we found a strong genetic interaction between Tbx1 and Pxn.

Funding: Ministero dell'Istruzione, dell'Università e della Ricerca (MIUR) (2022JAEY4L) to Daniela Alfano. Ministero dell'Istruzione, dell'Università e della Ricerca (MIUR) (P2022ZXAJ9) to Daniela Alfano. Fondazione Telethon (FT) (GMR22T1012) to Antonio Baldini.

P_05

Cardiac outflow tract defects in mice lacking MRPL40 in neural crest cells

Caoyu Ji, Zhongzhou Yang

Nanjing University Medical School, Jiangsu Key Lab of Molecular Medicine, Nanjing, China

Background: Cardiac neural crest cells (NCCs) are pluripotent migratory cells involved in the formation of cardiac outflow tracts (OFT) and pharyngeal arch arteries. Disruption of cardiac NCCs results in a large proportion of congenital heart diseases, including 22q11.2 deletion syndrome (22q11.2DS). 22q11.2DS causes haplo-insufficiency of nearly 50 genes and multiple system disorders including congenital heart defects. *Mrpl40* (mitochondrial ribosomal protein large unit 40) is one of the genes deleted in the 22q11.2 DS but its physiological function is still elusive.

Methods: To study the role of MRPL40 in cardiac NCCs during OFT development, we use the *Wnt1-Cre* delete its encoding gene *Mrpl40* specifically in NCCs.

Results and Conclusion: Mutant mice display cardiovascular and craniofacial defects. The cardiac phenotypes including double outlet of right ventricle (DORV) and defective OFT elongation. Reduced levels of PlexinA2 signals were observed among different cell populations. Such changes may affect the recruitment of NCCs into the OFT mesenchyme, causing OFT septation delay and misposition. Collectively, this work elucidates the key role of MRPL40 in NCCs-derived OFT development and provides new insights in understanding OFT septation and rotation. Meanwhile, this study helps to decipher congenital heart defects in 22q11.2DS.

P_06

Jagged1 conditional deletion and patient-based single variant mouse models of Alagille syndrome – morphology and physiology.

Hana Kolesová[1], Kristyna Neffeová[1], Eva Zábrodská[1], Veronika Olejníčková[1], Anna Maria Frontino[2], Jan Mašek[2]

[1] *Institute of Anatomy, First Faculty of Medicine, Charles University, Prague, Czech Republic*
[2] *Faculty of Science, Charles University, Prague, Czech Republic*

Jagged 1 (Jag1) is known to play an important role not only in cardiac development, causing Alagille syndrome. Even partial deletion of Jag1 is causing severe congenital heart defects. In this study we are analyzing the effects of loss of Jag1 function on embryonic and postnatal hearts in models of conditional deletion of Jag1 and a single nucleotide variant in Jag1, which was selected based on data from patients with Alagille syndrome.

We analyzed prenatal as well as postnatal hearts of Jag1 floxed, Islet1-cre mouse line and two lines with single nucleotide variant in Jag1. Morphology of hearts was studied on histological sections. Physiological functions were assessed using ultrasound in vivo imaging (VEVO) Heart activation pattern was analyzed using optical mapping.

We found that Jag1, Islet1-cre mouse line exhibits severe heart defects during embryonic development, with phenotype ranging from mild abnormalities to Double Outlet Right Ventricle (DORV) with ventricular septal defect (VSD) and valve defects. Surviving postnatal mice present with milder defects, especially valve malformations. Activation of the heart was severely affected in embryonic as well as in adult hearts (however with milder morphological phenotype). Adult hearts ultrasonographical analysis showed dyssynchronous contraction of ventricles and other physiological abnormalities. Patients-based mouse model with single nucleotide variant in postnatal stages exhibits mild defects in morphology (valve defects) and physiological abnormalities.

Our results confirm that Jag1 is an important player in heart development and its disruption even in single nucleotide variant is causing congenital heart defects. The humanized mouse models of Alagille syndrome help to understand etiology and pathogenesis of related congenital heart disease.

Supported by Charles University Cooperatio 207029 Cardiovascular Science.

https://anat.lf1.cuni.cz/english/pracovnici/kolesova.php

P_07

Alteration of the lactate dehydrogenase enzymes expression profile and metabolic response during embryonic development in Tbx1 haploinsufficient mouse

Erika D'agostino[1*], Sara Veneziano[1*], Marchesa Bilio[1], Giulia Sgueglia [2], Carmela Dell'Aversana [2-3], Debora Paris[4], Antonio Baldini[5], Gabriella Lania[1]

[1] *Institute of Genetics and Biophysics, National Research Council (CNR), Naples 80131, Italy*

[2] *Department of Precision Medicine, University of Campania 'Luigi Vanvitelli', Naples, Italy*

[3] *Institute of Experimental Endocrinology and Oncology 'Gaetano Salvatore' (IEOS)-National Research Council (CNR), Naples, Italy*

[4] *Institute of Biomolecular Chemistry, National Research Council, 80078, Pozzuoli (Naples), Italy*

[5] *Department of Molecular Medicine and Medical Biotechnology, University of Naples "Federico II", 80131 Naples, Italy*

**Equal contribution*

Introduction: Tbx1 is a transcription factor known for its crucial role in cardiovascular development. We have previously demonstrated that *Tbx1* haploinsufficiency deregulates certain developmental pathways. Additionally, transcriptome profiling of *Tbx1*$^{+/-}$ embryos revealed significant alterations in the expression of genes involved in cellular respiration (1,2). Given the essential role of cellular respiration in generating energy for cellular processes, we sought to investigate how TBX1 might influence these metabolic pathways.

Methods: We utilized a combination of qRT-PCR, immunofluorescence staining, flow cytometry, and the Agilent Seahorse platform to examine the correlation between *Tbx1* dosage changes and cellular respiration.

Results: Our analysis showed that more than 10% of the differentially expressed genes in *Tbx1*$^{+/-}$ embryos, compared to wild-type (WT) controls, are involved in cellular respiration. To validate these findings, we further examined the expression of key glycolytic and mitochondrial genes in *Tbx1*$^{+/-}$ mouse embryos and found a significant overexpression of *Ldhb*, which encodes lactate dehydrogenase B (LDHB). Notably, LDHB expression was also increased in the pharyngeal mesoderm and outflow tract (OFT) of *Tbx1*$^{+/-}$ embryos at embryonic day 9.5 (E9.5).

Given that LDHB catalyzes the conversion of lactate to pyruvate, we hypothesized that the upregulation of *Ldhb* could lead to an excess of pyruvate, potentially resulting in increased ATP production and oxygen consumption. Interestingly, we observed a reduction in reactive oxygen species (ROS) concentration in *Tbx1*$^{+/-}$ embryos. To further investigate the link between TBX1 and mitochondrial activity, we knocked down Tbx1 expression in differentiating C2C12 cells and assessed both *Ldhb* expression and cellular respiration. Our results demonstrated a significant downregulation of key myogenic genes (*Mef2C* and *MyoD*) alongside the overexpression of *Ldhb*, accompanied by increased basal oxygen consumption and ATP production. This metabolic response is known to be a hallmark of the differentiation process (3).

Our preliminary findings suggest that Tbx1 modulates mitochondrial activity, potentially through its effects on lactate dehydrogenase and cellular respiration. Further studies are required to elucidate these connections in greater detail with respect to developmental processes.

1) Lania, G., A. Bresciani, M. Bisbocci, A. Francone, V. Colonna, S. Altamura, and A. Baldini. 2016. Vitamin B12 ameliorates the phenotype of a mouse model of DiGeorge syndrome. *Hum. Mol. Genet.* 25:4369–4375. doi:10.1093/hmg/ddw267.
2) Lania, G., M. Franzese, N. Adachi, M. Bilio, G. Flore, A. Russo, E. D'Agostino, C. Angelini, R.G. Kelly, and A. Baldini. 2022. A phenotypic rescue approach identifies lineage regionalization defects in a mouse model of DiGeorge syndrome. *Dis Model Mech*. 15:dmm049415. doi:10.1242/dmm.049415.
3) Khacho M. and R.S. Slack. 2017 Mitochondrial activity in the regulation of stem cell self-renewal and differentiation. *Curr. Opin. Cell Biol.*,49, pp. 1-8. doi:10.1016/j.ceb.2017.11.003.

Funding: Research projects of significant national interest PRIN 2022 financed under the PNRR Ministry.

P_08

Exploring the Contribution of Structural Variants to Exome-Negative Congenital Heart Disease Cases

R. Masmaliyeva[1,2], C. Vey[1], G. Dombrowsky[1], A. Rump[1], E. Audain[1] & M-P. Hitz[1,2*]

[1] *Institute for Medical Genetics, Klinikum Oldenburg, Oldenburg, Germany*
[2] *Carl von Ossietzky University of Oldenburg, Oldenburg, Germany*

Background: Congenital Heart Disease (CHD) is a major cause of foetal and infant mortality, with an incidence of 7-9 per 1000 live births. Structural variations (SVs) in the genome, often undetectable by exome sequencing (ES), are less studied. We analyzed genome sequencing (GS) data from 615 ES-negative CHD cases to identify novel SVs. Our findings highlight the critical role of SVs in CHD, emphasizing the value of GS in uncovering genetic variants missed by ES, and providing new insights into the genetic basis of CHD.

Methods: Samples were collected through the "Competence Network for Congenital Heart Defects" and sequenced using the NovaSeq6000 (Illumina). FASTQ data was processed via the Nextflow-based pipeline sarek. Structural variants (SVs) were primarily detected with Manta v1.3.0 and confirmed with Delly v2.0, with consensus defined by an 80% reciprocal overlap. Low-quality SVs and those overlapping (>50%) centromeric/telomeric regions were filtered out. The remaining variants were annotated using VEP v112 and AnnotSV v3.4.2 to create an analysis-ready SV dataset.

Results: After QC and filtering, 786,809 unique SVs were identified, averaging 9,148 per genome across deletions (DEL), duplications (DUP), insertions (INS), inversions (INV), and translocations (BND), consistent with previous studies (gnomAD-SV, PMID:32461652; 1K-WGS, PMID:36055201). Deletions and duplications mostly ranged from 50-5000 bp. The majority of deletions (~85%) and duplications (~93%) had a cohort-specific allele frequency (MAF) below 0.01. A total of 2,676 pathogenic deletions (2,576 singletons) and 1,785 pathogenic duplications (1,702 singletons) were identified.Functional annotation showed that over 80% of deletions and 55% of duplications affect non-coding regions. Deletions impacted 5,138 protein-coding genes, while duplications affected 6,837, with 214 and 300 of these being CHD-associated, respectively. Translocations and inversions rarely affected known CHD genes, with only one translocation involving ZFPM2 in four cases and inversions in 16 cases affecting 16 CHD genes.

Visual inspection confirmed five deletions in CHD probands affecting key autosomal dominant CHD genes (2x GATA6, NOTCH1, EHMT1, A2ML1). Deletions in GATA6 were found in two patients with double outlet right ventricle and pulmonic stenosis. A patient with aortic coarctation had a deletion in NOTCH1 (exons 3-34), another with pulmonic stenosis had a deletion in EHMT1 (exon 4), and a patient with aortic coarctation and a bicuspid aortic valve had a deletion in A2ML1 (exons 13-28). Overall, ten unexplained deletions were identified: four in autosomal recessive and six in autosomal dominant genes.

Conclusion: Our findings suggest and reinforce the importance of SVs in the etiology of CHD. A larger cohort size and further refinement of the SV discovery pipeline is required for a more accurate evaluation. Despite the consensus approach adopted in this study (involving different SV callers), we have observed a significant proportion of SVs with unclear breakpoints.

Funding: This work was partially funded by Illumina, Inc.

P_09

Physiological and Morphological Consequences of Jagged1 Deletion in Mouse Model of Tetralogy of Fallot

Kristýna Neffeová[1,2], Eva Zábrodská[1], Veronika Olejníčková[1,2], David Sedmera[1], Hana Kolesová[1]

[1] *Institute of Anatomy, First Faculty of Medicine, Charles University, Prague, Czech Republic*
[2] *Institute of Physiology, The Czech Academy of Sciences, Prague, Czech Republic*

Background: The Notch signaling pathway plays a crucial role in embryonic development and adult homeostasis. Mutations in the human Jagged1 (Jag1) gene, which encodes a ligand for the Notch receptor, are responsible for Alagille syndrome. Symptoms of this inherited disease may include various forms of Tetralogy of Fallot.

Methods: Here, we generated Jag1$^{flox/flox}$ Islet1$^{Cre/+}$ mice with conditional Jag1 deletion in the cardiac outflow tract to investigate the impact of Jag1 mutations on cardiac morphology and physiology. Mice with conditional deletion exhibited severe cardiac malformations typical for Tetralogy of Fallot. Islet1 is also express in sinoatrial and atrioventricular nodes, therefore we used the optical mapping to visualize changes in patterning of the cardiac conduction system.

Results and Conclusion: The analysis of E14.5, E16.5 embryos and adult mice showed changes in the activation pattern. In controls, we showed maturated activation from apex to base with two separate activation centres. Mutant embryonic hearts revealed activation only from the left ventricle, indicating a perturbed function of the right bundle branch. In mutant adult mice, activation occurred at additional activation centres, distinguishing them from controls where excitation is conducted from a single site at the apex. Vevo ultrasound imaging physiological analysis was performed only on adult heterozygotes, because of the postnatal mortality of the homozygotes. Most of the monitored hemodynamical parameters did not show significant differences. However, spackle-based strain analysis revealed vulnerable areas of contractile defect that generate mechanical dyssynchrony pronounced mostly at anterior wall. In our study, we demonstrated morphological and electrophysiological alterations resulting from conditional deletion of Jag1. Embryonic mice exhibited malformations and irregular activation patterns. Severe malformations were less prevalent in adult mice, primarily due to the survival of heterozygotes and an increased mortality rate among mice displaying severe congenital defects. Nevertheless, surviving animals exhibited abnormal electrophysiological changes along with physiological alterations resulting in dyssynchronous myocardial contractions observed during strain analysis.

P_10

Defining the role of Calcium homeostasis modulator 2 (CALHM2) in cardiac metabolism and development

Thomas Nico, Dona Josh, Andrew Robson, Lisa Heather, Nicola Smart

Deparment of Physiology, Anatomy and Genetics, Sherrington Building, Sherrington Road, University of Oxford, Oxford, OX1 3PT, UK

Background: The metabolic switch, from glycolysis to fatty acid oxidation, associated with cardiac development is essential for myocyte differentiation and adult cardiac structure and function. Our work has identified CALHM2 (Calcium homeostasis modulator protein 2) as a gene associated with congenital heart disease (CHD) that potentially results from a defect in metabolic maturation of the heart. Given the links between CALHM2 in mitochondria, and mitochondrial metabolic function in cardiac development, our work has focused on characterising CALHM2 in embryonic mouse hearts throughout development and into early adulthood.

Methods: Utilizing a Bio-ID approach to tag interacting partners of CALHM2 and identify them using mass-spec. This will allow for a complete picture of CALHM2s role in mitochondrial metabolism and how it contributes to substrate selection in the developing heart. Going forward this work will be complemented by studies using a *Calhm2*$^{-/-}$ mouse to determine the molecular function of CALHM2 in the developing heart, and how loss of function leads to alterations to metabolic processes in the mitochondria and ultimately CHD.

Results and Conclusion: Our work has already identified the mitochondrial trifunctional protein (mTFP), a heterodimeric enzyme consisting of ECHA and ECHB subunits, as a potential interacting partner of CALHM2. This complex localises to the inner mitochondrial membrane where it facilitates fatty acid β-oxidation. Removing CALHM2 by siRNA knockdown results in reduced localisation of ECHA to the inner mitochondrial membrane. Rather ECHA accumulates in the area surrounding mitochondria, seemingly due to a defect in import machinery. These data suggest that CALHM2 may be responsible for the import of fatty acid oxidation machinery into the mitochondria. My work is focused on confirming this interaction in cells and heart tissue to determine the role of CALHM2 in fatty acid oxidation throughout development.

Funding: This work is supported by a British Heart Foundation Project Grant PG/22/10892 - CALHM2, a congenital heart disease gene regulating cardiac development, metabolism and function.

P_11

3D negative-space segmentation of extracellular matrix in the early heart tube uncovers roles for lateralised matrix organisation in heart morphogenesis

Juliana Sánchez-Posada and Emily S. Noël

School of Biosciences and Bateson Centre, University of Sheffield, Western Bank, Sheffield, S10 2TN, UK

Background: The extracellular matrix (ECM) is a complex array of water, proteins and sugars which mediates the chemical and biomechanical signals that shape developing tissues. The myocardial and endocardial layers of the embryonic heart tube are separated by ECM, and we previously identified that prior to heart morphogenesis this ECM is regionally expanded, characterised by an atrial-specific left-sided expansion of matrix volume in the heart tube. However, visualising and linking dynamics of ECM organisation with ongoing tissue morphogenesis is challenging since sample processing can impact matrix hydration or tissue structure.

Methods: We developed morphoHeart, a morphometric image analysis software that facilitates 3D segmentation of cardiac tissues from live lightsheet images of the developing zebrafish heart. To visualise label-free ECM-space, morphoHeart performs negative space segmentation of ECM volume, allowing us to link 3D ECM dynamics with cardiac morphogenesis during heart development.

Results and Conclusion: We find that early ECM regionalisation is maintained as the heart develops but becomes repositioned during atrial morphogenesis, and is linked to the orientation of atrial ballooning. To understand if defects in heart tube lateralisation result in abnormal ECM-regionalisation and hence disrupted morphogenesis, we investigated hearts in which *spaw* (zebrafish Nodal homolog which regulates embryonic left-right asymmetry) is disrupted. ECM asymmetry in the heart tube of *spaw* mutants is established but mispositioned, and irrespective of looping direction translates into regionalised ECM expansion in the atrial outer curvature at later stages. Surprisingly we also found that *spaw* has chamber-specific effects on longer-term ECM remodelling and timely chamber growth during morphogenesis. Analysis of combinatorial ECM and *spaw* mutants revealed distinct spatiotemporal roles in the early heart for ECM-regionalisation and lateralisation in ongoing morphogenesis. We propose a model whereby an ECM asymmetry is established in the heart tube and oriented by laterality cues, which together help shape cardiac morphology.

Funding: This work was supported by a BBSRC Standard Grant BB/W004305/1 and a British Heart Foundation Fellowship award FS/16/37/32347.

Links: morphoHeart preprint

P_12

Left-right asymmetry in a specific cardiac progenitor population stratifies cardiac phenotypes in a mouse model of motile ciliopathy

Amaia Ochandorena-Saa*[1], Emeline Perthame[1,2], Alexander Chamolly[3], Zoé Oulerich[1], Thierry Blisnick[4], Philippe Bastin[4] and Sigolène Meilhac[1]

**presenting author*

[1] *Université Paris Cité, Imagine - Institut Pasteur Unit of Heart Morphogenesis, INSERM UMR1163, Paris, France*

[2] *Bioinformatics and Biostatistics Hub, Institut Pasteur, Paris, France*

[3] *Developmental and Stem Cell Biology Department, CNRS UMR3738, Institut Pasteur, Paris, France*

[4] *Trypanosome Cell Biology Unit, Institut Pasteur, INSERM U1201, Paris, France*

Background: Left-right asymmetry is essential for the mammalian heart function, as a double left-right pump. Asymmetry emerges in the embryonic node, in which motile cilia create a leftward fluid flow. This induces the left-sided expression of the secreted factor NODAL, which is essential to pattern heart precursors and shape the looped heart tube. Laterality defects are often described as random, by lack of understanding of the underlying mechanisms. We showed previously that inactivation of *Nodal* causes heterotaxy with right isomerism and complex congenital heart defects. However, *Nodal* mutants never display complete reversal of asymmetry, termed situs inversus totalis. We now disrupted left-right asymmetry upstream of *Nodal*.

Methods: We used *Ccdc40* mutants as a model to impair node motile cilia. We analysed the phenotype at embryonic and perinatal stages. We measured asymmetry at different levels (node flow, gene expression, heart shape and visceral organs), based on a range of 3D imaging approaches and quantitative image analyses. We carried out direct correlation of *Nodal* expression with node flow or heart shape. We performed RNA-sequencing of paired left-right heart fields, to extract the transcriptomic signature of the different phenotypic categories and validated the results by sensitive RNA in situ hybridisation.

Results and Conclusion: Inactivation of *Ccdc40* abrogates node flow. The mutant phenotype is different to *Nodal* mutants, showing partial penetrance of situs inversus totalis and heterotaxy with left isomerism at birth. Earlier in development, *Ccdc40* mutants also segregate into three groups based on looping defects and *Nodal* patterning defects. Our left-right transcriptomic screen of heart progenitors uncovers asymmetry in a specific population as the best predictor of the phenotype. Our work provides novel insights into the mechanisms of asymmetric heart morphogenesis and the origin of complex congenital heart defects.

Funding: This work was supported by the Institut Pasteur, Institut Imagine, Inserm, FRM, ANR ‘‘Investissements d’avenir’’ program and the Philanthropy Department of Mutuelles AXA.

P_13

Integrin α8 regulates epicardium development in zebrafish

Ganesh Wagh[#], Swarnav Bhakta[#], Chinmoy Patra*

# Contributed equally; * Presenter

Department of Developmental Biology, Agharkar Research Institute, Pune

Integrins, a membrane-bound heterodimeric cell surface receptor, comprises two glycoprotein subunits, α and β. Among them, ITGA8, a member of the integrin α family, forms a functional unit with the β1 subunit. It plays a crucial in development and disease. Knockout of *Itga8* in mice results in kidney morphogenesis defects and postnatal lethality. Zebrafish studies revealed *itga8* mutations causing craniofacial defects. However, spatiotemporal expression of ITGA8 in cardiogenesis and its role in cardiac morphogenesis has yet remained unexplored. Our present RNA *in situ* hybridization and reporter gene expression analysis using the Tg(*itga8*:EGFP) transgenic line identified that *itga8* is expressed in developing hearts from 60 hpf onwards. Detailed analysis found, *itga8* is exclusively expressed in the proepicardial tissue and epicardial cells of the heart. Loss-of-function analysis on *itga8*$^{-/-}$ embryos identified decreased epicardial cell migration in *itga8*$^{-/-}$ compared to their wild-type siblings. Taken together, gene expression and loss-of-function data showed that Itga8 is required for epicardium development in zebrafish. The findings of this study illuminate the significance of Itga8 in cardiac processes, advancing our understanding of integrins in vertebrate cardiovascular development.

P_14

Identification of a *NOTCH1*-specific methylation signature in non-syndromic CHD patients

G. Dombrowsky[1,2]#, L. van der Laan[3,4]#, A. Silva[4], J. Breckpot[5,6], E. Audain[1,2], A. Wilsdon[7], M.A. Levy[8], N. Vos[3], S. Klaassen[9,10], F. Berger[10], S. Dittrich[11], B. Stiller[12], H. Abdul-Khaliq[13], I. Dähnert[14], F. Bu'Lock[15], T. Pickardt[16], U. Bauer[16], H-H. Kramer[1], A. Uebing[1], S. Loughna[7], D.J. Brooks[7], P. Henneman[3], B. Sadikovic[4] ‡, M-P. Hitz[1,2] ‡, AV. Postma[3,17]‡

# and ‡ These authors jointly coordinated this work

[1] Department of Congenital Heart Disease and Pediatric Cardiology, University Hospital of Schleswig-Holstein, Kiel, Germany [2] Institute for Medical Genetics, Klinikum Oldenburg, Oldenburg, Germany [3] Department of Human Genetics, Amsterdam Reproduction and Development Research Institute, Amsterdam University Medical Center, University of Amsterdam, Meibergdreef 9, 1105 AZ Amsterdam, The Netherlands [4] Department of Pathology and Laboratory Medicine, Western University, London, ON N6A 3K7, Canada [5] Center for Human Genetics, University Hospitals Leuven, Leuven, Belgium [6] Department of Human Genetics, University of Leuven, KU Leuven, Leuven, Belgium [7] School of Life Sciences, University of Nottingham, Nottingham, United Kingdom. [8] Verspeeten Clinical Genome Centre, London Health Sciences Centre, London, ON N6A 5W9, Canada. [9] Experimental and Clinical Research Center (ECRC), Charité - Universitätsmedizin Berlin and Max Delbrück Center, Berlin, Germany [10] Deutsches Herzzentrum der Charité, Dept. of Congenital Heart Disease-Pediatric Cardiology, Berlin, Germany [11] Department of Pediatric Cardiology, University Hospital Erlangen, Friedrich-Alexander-University Erlangen-Nürnberg, Erlangen, Germany [12] Department of Congenital Heart Disease and Pediatric Cardiology, University Heart Center Freiburg-Bad Krozingen, Medical Center-University of Freiburg, Faculty of Medicine, Freiburg, Germany [13] Department of Pediatrics and Pediatric Intensive Care Medicine, University Hospital of Saarland, Homburg, Germany [14] Department of Pediatric Cardiology, Heart Center Leipzig at University of Leipzig, Leipzig, Germany [15] Congenital and Paediatric Cardiology, East Midlands Congenital Heart Centre and University of Leicester, Glenfield Hospital, Leicester, United Kingdom [16] German Competence Network for Congenital Heart Defects, National Register for Congenital Heart Defects, Berlin, Germany [17] Department of Medical Biology, Amsterdam University Medical Centers AUMC, Amsterdam, the Netherlands

Background: *NOTCH1*-haploinsufficiency is a major monogenetic cause of congenital heart defects (CHDs). Here, we present a *NOTCH1*-specific DNA-methylation, which can aid variant classification, thus improving counselling of patients.

Methods: In an exome-based CHD-cohort, we identified *NOTCH1*-variants in 34 cases, further extended by additional samples with similar *NOTCH1*-variants. Samples were subjected to DNA methylation profiling using the Illumina Infinium MethylationEPIC array. Raw data was Quality Control (QC) processed and analyzed using the EpiSign™ Discovery Software in R. DNA methylation profiles of controls (EpiSign Knowledge Database) were age and sex matched. A linear model (limma) was applied to obtain differential methylated positions between patients and controls. An unsupervised model was applied to evaluate the robustness of the classifying probes and a support vector machine was used for the final DNAm signature.

Results and Conclusion: All included samples passed QC-criteria. A specific DNA methylation signature was established using patients harbouring pathogenic *NOTCH1*-variants and control samples. Analysis of included VUSes demonstrated overlap with the *NOTCH1*-signature in 3 cases. **Moreover, the signature showed robust specificity concerning >50 other episignatures related to neurodevelopmental disorders.** Our results represent the first non-syndromic signature and illustrates how DNA-methylation analysis can improve *NOTCH1* variant classification.

Funding: Funding for this study is provided in part by the Government of Canada through Genome Canada and the Ontario Genomics Institute (OGI-188). This work was partly funded by PROCEED project ERA PerMED joint Translational Call Initiative (DLR Funding reference 699 number: 01KU1919).

P_15

Heart defects in CrispR-Cas9 loss-of-function zebrafish for *tbx1* and *cyp26b1*

Maria Altomonte*[1,4], Pilar Casares Alaez*[1,4], Lianri Van Schalkwyk*[2,4], Clida Coutinho*[2,4], Dilan Durmus[3], Hamza Quraishi[3], and Catherine Roberts[4]

*These authors contributed equally to the work

[1] *MSc Human Molecular Genetics, Imperial College, London.*

[2] *MSc Genomic Medicine, St George's University of London*

[3] *BSc Biomedical Science St George's University of London*

[4] *Institute of Medical, Biomedical and Allied Health Education, St George's University of London, Cranmer Terrace, London, SW17 0RE, UK*

We have previously presented murine data suggesting that *Cyp26b1* (and other *Cyp26* genes) may be a target for *Tbx1* during cardiovascular development, with significant arterial pole and pharyngeal arch artery abnormalities found in *Cyp26b1*$^{-/-}$ embryos. The data suggests a genetic interaction between these genes, but this has been challenging to prove in the mouse.

During the pandemic, access to the *Cyp26b1* LOF mouse line was lost. Instead we have generated F0 CrispR-Cas9 LOF models for both genes in the zebrafish embryo. Crispants for *tbx1* recapitulate pharyngeal and cardiac defects seen in *vgo*$^{tm208/\ tm208}$ (*tbx1* LOF mutations) at high efficiency (80%). LOF mutants for *cyp26b1* have previously reported pharyngeal arch and skeletal defects. These were observed in at least 70% of injected embryos plus previously unreported cardiac phenotypes. Moreover, injection of RNPs against both genes increased the frequency of a phenotype to above 90%. The incidence of cardiac oedema observed at 3dpf more than doubles, suggesting a possible genetic interaction between *tbx1* and *cyp26b1*.

We used cardiac markers (*gata4/5*, *nkx2.5*, *has2*, *tbx5.1* and *vmhc*) to establish dysregulated expression in *cyp26b1* LOF injected zebrafish embryos compared to un-injected controls at different time points. Overall, results suggested a loss of second heart field/ventricular markers compared to others. Analysis of markers in double-crispants supports genetic interaction of these genes during cardiac development, but remains to be fully confirmed.

A similar experimental approach used known and novel retinoic-acid metabolising agents (RAMBAs) which act as pan-CYP26 chemical inhibitors. Analysis comparing phenotypes and the efficacy of different RAMBAs is ongoing, but has yielded similar results to CrispR-Cas9 deletion; following treatment at 6hpf, cardiac defects at 24h are observed, with second heart field/ventricular progenitors most affected. The novel RAMBAs produce similar effects to known agents, but these cardiac phenotypes appear to be less severe.

P_16

MCM6 controls cilium and heart development by facilitating canonical WNT signaling

Steffen-Alexander Sailer[1], Marco Groth[2], Karol Safranski[2], Martin D. Burkhalter[1], Melanie Philipp[1]

[1] *Department of Experimental and Clinical Pharmacology and Pharmacogenomics, Section of Pharmacogenomics, Eberhard-Karls-University Tübingen, Tübingen, Germany*
[2] *Leibniz Institute on Aging, Fritz Lipmann Institute (FLI), Jena, Germany*

Background: Cilia are small antenna-like structures extending from the surface of nearly all post-mitotic cells, which play important roles particularly during embryonic development, including cardiac development. The best understood mechanism how cilia guide heart development is left-right asymmetry determination and hence oriented organ development. When cilia are defective a condition called heterotaxy may develop which is often associated with congenital heart defects. We have recently shown that proteins facilitating DNA replication possess non-canonical functions in quiescent cells. Here, we expand our portfolio of replication proteins and present MCM6 as a novel factor controlling cilium as well as heart development.

Methods: We used a combination of cell culture and zebrafish experiments to investigate the precise function of MCM6 during cilium and heart formation.

Results and Conclusion: We discovered that MCM6 is essential for cilium elongation in human fibroblasts. RNAseq revealed downregulation of WNT2. Using different assays, we demonstrated that MCM6 knockdown-induced cilium shortening is due to loss of canonical WNT signaling. In zebrafish, cilia and laterality defects induced by Mcm6 loss-of-function could be rescued by Wnt2 reconstitution within the organ of laterality. As WNT2 has previously been linked to sinoatrial node development and normal cardiac function in rodents, we analyzed the hearts of Mcm6-depleted embryos more closely. Both, loss of Mcm6 or Wnt2 resulted in bradyarrhythmia and disruption of normal cardiac patterning.

Taken together we uncovered a novel noncanonical function of MCM6 and show that MCM6 regulates ciliogenesis and subsequent heart development through WNT2-mediated canonical WNT signaling.

P_17

Investigating the role of alternative transcripts in Adams-Oliver Syndrome

Corinna M. Snashall, Emma L. Armitage, Emily S. Noel

School of Biosciences, University of Sheffield, Firth Court, Western Bank, Sheffield, S10 2TN

Background: Adams-Oliver Syndrome (AOS) is a rare congenital disorder notable for variable penetrance and multisystemic phenotypes. Congenital heart defects occur in ~20% of patients. Despite identification of 6 genes that cause AOS when mutated (DOCK6, EOGT, NOTCH1, RBPJ, ARHGAP31 and DLL4), currently only 50% of patients receive a genetic diagnosis through exon sequencing.

Efforts to recapitulate the cardiac phenotype of AOS in zebrafish by targeting causative genes identified an alternative, intron-retaining transcript of dock6 expressed specifically in the heart which may contribute to development of cardiac defects. This project aimed to investigate additional alternative transcripts expressed by AOS genes and their expression patterns.

Methods: Genome browsers were interrogated to identify alternative transcripts of AOS genes, particularly those containing retained intronic sequence. As alternative transcript annotation is limited in zebrafish, intron retention was predicted using structural and sequence conservation of human and zebrafish genomes.

Primers were designed to PCR-amplify intronic sequences from 26hpf zebrafish cDNA. These sequences were cloned into pCRII-TOPO vectors and used as templates to transcribe DIG-labelled mRNA probes, which were used for whole-mount in-situ hybridization in zebrafish embryos.

Results and Conclusion: 25 intronic sequences from 5/6 AOS genes were amplified, indicating alternative transcripts of AOS genes are expressed at 26hpf. In-situ hybridization analysis showed variable expression of intronic RNA across genes and targets. Alternative transcripts of dll4 showed expression in the heart and suggested expression of an unannotated antisense transcript. Alternative transcripts of other AOS genes showed localized expression in the brain, tail and vasculature.

In conclusion, despite poor annotation, multiple alternative transcripts are expressed by AOS genes in zebrafish, with many showing tissue-specific expression. Their roles are unknown but provide opportunities for investigation. Additionally, with many alternative transcripts annotated for human AOS genes, these findings may support expanded sequencing of introns in AOS patients, potentially increasing diagnoses.

Funding: University of Sheffield PGR Scholarship

https://emilynoelresearch.weebly.com

Poster Session 2 – Cardiac Progenitors

Global Developer, Manufacturer, and Supplier of High-Quality Reagents, Analytical Instruments, and Precision Diagnostics.

https://www.bio-techne.com

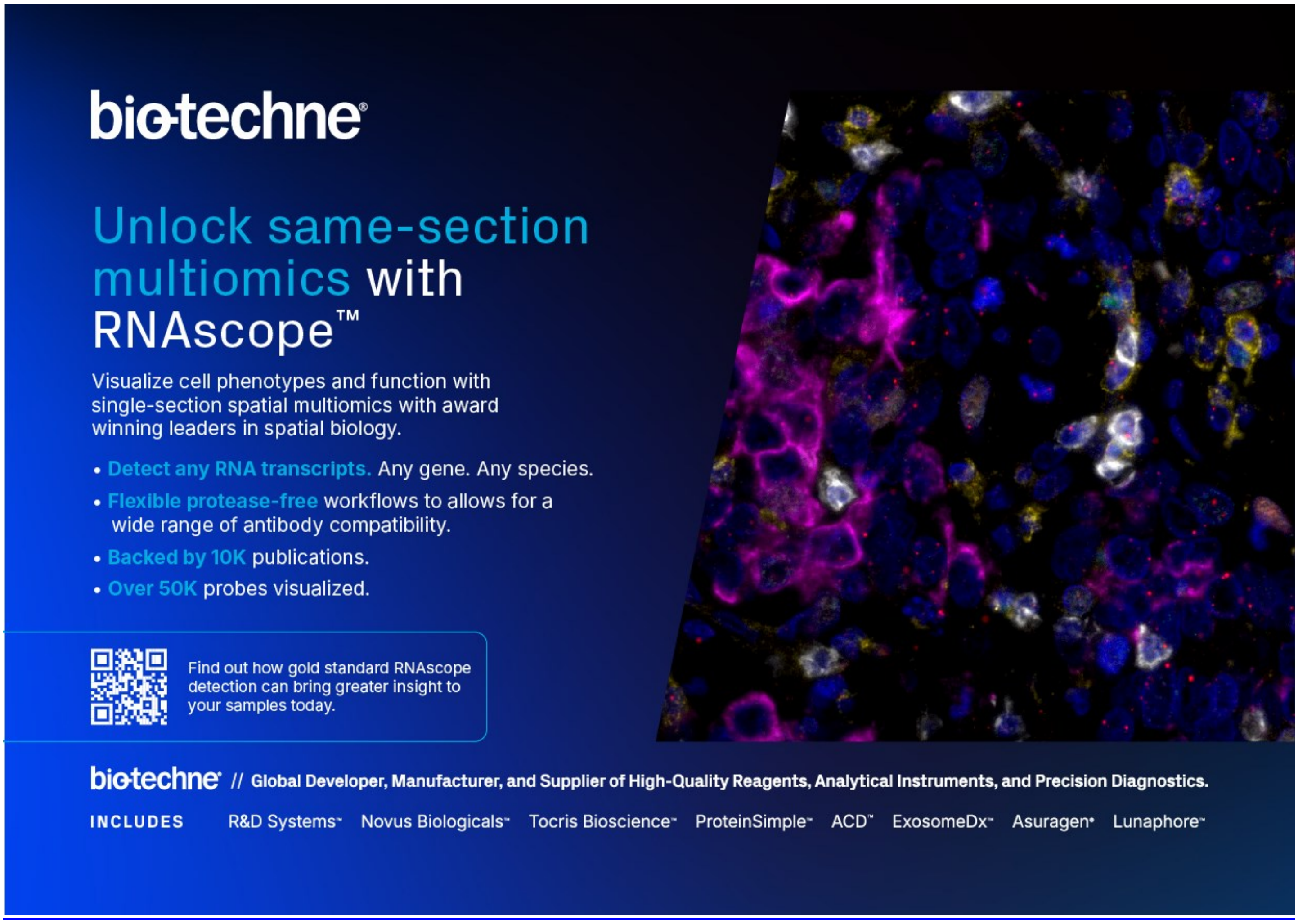

P_18

Tbx1 takes control of planar cell polarity through Daam1-dependent pathway

Daniela Alfano[1], Marchesa Bilio[1], Rosa Ferrentino[1], Olimpia Iacolare[1], Antonio Baldini[2]

[1] *CNR-Institute of Genetics and Biophysics Adriano Buzzati-Traverso, Via Pietro Castellino, Naples, Italy*
[2] *Department of Molecular Medicine and Medical Biotechnology, University of Naples Federico II, Naples, Italy*

Background: Epithelial cardiac progenitor cells of the second heart field (SHF) contribute to growth of the vertebrate heart tube by progressive addition of cells from the dorsal pericardial wall to the cardiac poles. Perturbation of SHF development, including defects in apicobasal or planar polarity, results in shortening of the heart tube and a spectrum of congenital heart defects. Planar cell polarity (PCP) pathway has been proposed to be part of this process by facilitating the incorporation, by intercalation, of mesenchymal cells into the epithelial sheet of the DPW (1,2). TBX1 regulates Wnt5a expression in the SHF (3), suggesting that at least some of the tissue architectural roles of TBX1 may operate through the non-canonical WNT/PCP pathways. However, Wnt5a$^{-/-}$;Tbx1$^{-/-}$ embryos have much more severe SHF-derived heart defects than the individual mutants, providing genetic evidence that the two genes also have non-overlapping functions in the SHF. Dishevelled-associated activator of morphogenesis 1 (Daam-1) is required for heart morphogenesis (4). Our bioinformatic analyses of genome-wide target gene data suggested that Daam-1 (and or -2) is a target of Tbx1.

Methods: In this study, using loss- and gain-of-function genetics approaches, we investigated whether Tbx1 takes control of PCP pathway. Specifically, we took advantage of both *in vitro* (undifferentiated myoblast C2C12 cells) and *in vivo* models (E 9.0 Tbx1 mutant embryos, Tbx1$^{-/-}$).

Results and Conclusion: We found that Tbx1 positively regulates Daam1 gene in both in vitro and in vivo models. Consistently, Tbx1 functionally impacts the PCP signaling pathway; in fact, the cell migration non-canonical Wnt pathway-dependent was affected in Tbx1-depleted cells (Tbx1^{KD}). Daam1 ectopically expressed is able to rescue the defect of migration of Tbx1^{KD} cells. Furthermore, the signaling cascade downstream to PCP pathway (small Rho GTPases activation and JNK phosphorylation) which is dysregulated in cells with high dosage of Tbx1 was fully reverted when Daam1 was inhibited. Dishevelled planar cell polarity pathway is involved also in the mitotic spindle orientation. Intriguingly, we found that E9.5 Tbx1 -/- mutant embryos showed an alteration of spindle orientation compared to WT, suggesting that PCP pathway is altered also in Tbx1 mutant embryo. Moreover, non-canonical Wnt pathway is altered in Tbx1 loss of function system in which other PCP components are dysregulated following Tbx1 deletion (Dvl2, Lgl1, aPKC). These findings suggest that Tbx1 impacts the PCP signaling.

Funding: Ministero dell'Istruzione, dell'Università e della Ricerca (MIUR) (2022JAEY4L) to Daniela Alfano. Ministero dell'Istruzione, dell'Università e della Ricerca (MIUR) (P2022ZXAJ9) to Daniela Alfano. Fondazione Telethon (FT) (GMR22T1012) to Antonio Baldini.

References:

1) Sinha T, Wang B, Evans S, Wynshaw-Boris A, Wang J. (2012). Disheveled mediated planar cell polarity signaling is required in the second heart field lineage for outflow tract morphogenesis. *Dev Biol*. 370(1):135-44. doi: 10.1016/j.ydbio.2012.07.023.
2) Li D, Sinha T, Ajima R, Seo HS, Yamaguchi TP, Wang J. (2016). Spatial regulation of cell cohesion by Wnt5a during second heart field progenitor deployment. *Dev Biol*.412(1):18-31. doi: 10.1016/j.ydbio.2016.02.017.
3) Chen L, Fulcoli FG, Ferrentino R, Martucciello S, Illingworth EA, Baldini A. (2012). Transcriptional control in cardiac progenitors: Tbx1 interacts with the BAF chromatin remodeling complex and regulates Wnt5a. *PLoS Genet*. 8(3):e1002571. doi: 10.1371/journal.pgen.1002571
4) Li D, Hallett MA, Zhu W, Rubart M, Liu Y, Yang Z, Chen H, Haneline LS, Chan RJ, Schwartz RJ, Field LJ, Atkinson SJ, Shou W. (2011). Dishevelled-associated activator of morphogenesis 1 (Daam1) is required for heart morphogenesis. *Development*.138(2):303-15. doi: 10.1242/dev.055566.

P_19

Characterization of DCHS1 Expression in Developing Myocardial Free Wall

Kathryn Byerly, Ranan Phookan, Hannah Parris, Emily Wilson, Lilong Guo, Jordan Morningstar, Taylor Petrucci, Cortney Gensemer, Erika Bistran, and Russell Norris

Department of Regenerative Medicine & Cell Biology, Medical University of South Carolina, Charleston, SC USA

Background: *Dchs1*, an atypical cadherin, was the first gene identified as causal to mitral valve prolapse (MVP). While haploinsufficiency partly explains the valve disease, complete knockout of the gene led to a reported high incidence of neonatal lethality in mice. Excised homozygous *Dchs1*$^{-/-}$ (knockout) P0 hearts appeared grossly immature (smaller and rounder). Therefore, we hypothesize that *Dchs1* is critical for normal maturation and morphogenesis of the developing heart.

Methods: To analyze normal expression of *Dchs1*, we engineered a mouse model with an HA-tagged *Dchs1* locus (referred to as *Dchs1-HA*) and analyzed it using immunohistochemistry and western analyses. To investigate *Dchs1's* role during development, a global knockout mouse model (*Dchs1*$^{-/-}$) was used and analyzed with single-nuclei RNA sequencing (snRNAseq), transmission electron microscopy (TEM), and western analyses.

Results and Conclusion: Analysis of the *Dchs1-HA* mice demonstrated DCHS1 protein localization to the cell membranes of embryonic and neonatal non-myocyte populations, including endothelial and epicardial cells, as well as fibroblasts. Notably, western analyses at embryonic and neonatal timepoints suggested a potential cleavage of the intracellular domain revealing bands corresponding to both the full-length DCHS1 protein and a smaller 50kDa band. The presence of this smaller molecular weight band was possibly dependent on cell density and was statistically increased during late gestation. Single-nuclei RNA sequencing (snRNAseq) of wild-type hearts corroborated non-myocyte expression, with a majority of transcripts identified in fibroblast and endothelial cell populations. It was found 100% of the homozygous *Dchs1*$^{-/-}$ mice died during neonatal stages with evidence of impaired cardiomyocyte differentiation via TEM. Western analyses and snRNAseq datasets showed a statistically significant increase in proliferation across all cardiac lineages, including myocytes. These findings demonstrate a critical role for Dchs1 in regulating myocyte and non-myocyte cell cycle in a cellular non-autonomous manner. Moreover, these data also suggest that cell density and the contribution of epicardial-derived fibroblasts in the heart likely exert a critical influence on myocyte cell-cycle exit through a *Dchs1* mechanism.

Funding: Supported by NIH R01HL131546 and R01HL149696

Hyperlink: https://www.thenorrislab.com

P_20

Sox9 in the second heart field and its role in outflow tract development and septation

Ray Deepe, Jenna Drummond, Hannah Tarolli, Inara Devji, Renélyn Wolters, Andrew Harvey, Andy Wessels

Department of Regenerative Medicine and Cell Biology Medical University of South Carolina

Background: Cardiac septation consists of a series of remodeling events in which parts of the developing heart become physically separated from each other by septal structures. Septal defects are among the most common congenital heart malformations, found in more than 50% of patients with congenital heart disease (CHD). Formation of the cardiac septa is critically dependent on the contribution of cells that derive from the Second Heart Field (SHF). The transcription factor Sox9 has been known for quite some time to play a crucial role in cardiac development, in particular as it relates to the formation of the atrioventricular (AV) cushions and AV valves. In this project we explore the importance of Sox9 expression in the SHF-derived cell populations that are involved in the development of the outflow tract (OFT) including the semilunar valves, developing OFT septum, and the OFT contribution to AV septation. We show that deletion of Sox9 from the SHF has a profound effect on the contribution of SHF-derived cells to the mesenchymal tissues of the OFT which, in turn, leads to valvuloseptal abnormalities.

Methods: Sox9 was conditionally deleted from the SHF using the Mef2-cre mouse model. H&E, immunohistochemistry, and 3D reconstructions were used to observe the impact of this deletion.

Results and Conclusion: Conditional deletion of Sox9 from the SHF resulted in atrioventricular septal defects and isolated ventricular septal defects, hypoplasia of the developing OFT septum and proximal cushions, and loss of SHF-derived cells in developing semilunar valves. These results highlight the critical importance of Sox9 in the SHF and its contribution to proper AV septation and OFT development.

Funding: NIH: R01-HL122906 (A.W.), R01-HL162913 (A.W.), T32-HL007260 (A.B.H.), T32-GM132055 (H.T.), AHA Support: 23PRE1014420 (A.B.H.)

P_21

miR-1 regulates Mef2c activity and Retinoic Acid signaling pathway during early differentiation of cardiac sinoatrial region

Diego Franco[1,2], Carlos Garcia-Padilla[1], Estefanía Lozano-Velasco[1,2], Virginio Garcia-Lopez[3,4], Amelia Aranega[1,2], Virginio Garcia-Martinez[4], Carmen Lopez-Sanchez[4]

[1] *Department of Experimental Biology, University of Jaen, 23071 Jaen, Spain*

[2] *Fundación Medina, 18016 Granada, Spain*

[3] *Department of Medical and Surgical Therapeutics, Pharmacology Area, Faculty of Medicine and Health Sciences, University of Extremadura, Badajoz, Spain*

[4] *Department of Human Anatomy and Embryology, Faculty of Medicine and Health Sciences, Institute of Molecular Pathology Biomarkers, University of Extremadura, Badajoz, Spain*

Background: A wide number of epigenetic factors, including microRNAs and histones modifications, are known to be capable of regulating gene expression without altering DNA sequence itself. In particular, miR-1 is considered the first essential microRNA in cardiac development. This experimental study aims to analyze miR-1 potential functions in early differentiation of the cardiac sinoatrial region.

Methods: Both primitive endocardial tubes of chick embryo culture were microinjected with premiR-1 and anti-miR-1, respectively, to perform gain- and loss-of-function experiments. Subsequently, embryos were subjected to whole mount in situ hybridization with Tbx5, Gata4 and Amhc1 probes, as well as immunohistochemistry with Mef2c, HDAC4, Calmodulin/Calm1 and pErk2/MAPK1 antibodies. Additionally, we carried out RT-qPCR analysis of control and experimental embryos, including CRABPI, CRABPII and retinoic acid receptors (RAR and RXR).

Results and Conclusion: Our results reveal that miR-1 increases specific atrial gene expression, including Tbx5, Gata4 and Amhc1, while this microRNA diminishes Mef2c expression. Also, we observed that miR-1 upregulates CRABPII and RARß, and downregulates CRABPI, which are three crucial factors in retinoic acid signaling pathway. Noticeably, we observed that miR-1 actively interacts with HDAC4, Calmodulin and Erk2/MAPK1, key factors involved in Mef2c regulation. All the above mentioned data suggest that miR-1 functions as an epigenetic factor orchestrating opposite actions between retinoic acid and Mef2c, fundamental to properly assign cardiac cells to their respective heart chambers.

P_22

Foxf1-mediated co-regulation of *miR-495* and *let-7c* modulates epicardial cell migration and myocardial specification

Juan Manuel Castillo-Casas[1], Ángel Dueñas[1], Francisco Hernández-Torres[1,2], Rita Carmona[3,4], Ramón Muñoz-Chápuli[3], Ana Dopazo[5,6], Rebeca Alvarez[5,6], Enrique Vázquez de Luis[5,6], Amelia E Aranega[1,2], Diego Franco[1,2], Estefanía Lozano-Velasco[1,2*]

[1] *Cardiovascular Development Group, Department of Experimental Biology, University of Jaén*

[2] *Fundacion Medina, Granada*

[3] *Department of Animal Biology, University of Málaga*

[4] *Department of Human Anatomy and Embryology, Legal Medicine and History of Science, Faculty of Medicine, University of Málaga*

[5] *Genomic Unit, Centro Nacional de Investigaciones Cardiovasculares, Madrid, Spain*

[6] *CIBER de Enfermedades Cardiovasculares (CIBERCV), Madrid, Spain*

Background: The proepicardium is a transitory cell cluster that develops at the junction between the sinus venosus and the posterior undifferentiated lateral plate mesenchyme that migrates onto the heart and spreads over the surface forming a single squamous epithelium, i.e. the embryonic epicardium. Our understanding of cell lineage contribution of the embryonic epicardium and its epicardial cell derivatives has greatly increased over the last decade, while our understanding of the molecular determinants of these processes remains poorly understood. In this study, we carried out a comprehensive characterization of coding and non-coding gene expression hallmarks at proepicardium and embryonic epicardium development.

Methods: Pregnant females of Wt1GFP/+ mice were used for proepicardium and embryonic epicardial cell collection. RNAseq analysis for mRNAs, long and short non-coding RNAs was performed to analyze the differential expression in these two critical points of development. Furthermore, molecular and cellular techniques as well as cell migration assays were conducted to investigate the biological function of transcription factors and microRNAs differentially expressed in proepicardium and embryonic epicardium during cardiogenesis.

Results and Conclusion: Among the differentially expressed microRNAs, *miR-495 and let-7c* exert significant effects on epicardial cell migration, underscoring the importance of their precise regulation for proper epicardium formation and the migration of epicardial-derived cells. Additionally, our analysis provides the first evidence that differentially expressed microRNAs in proepicardium and embryonic epicardium also regulate the expression of other microRNAs in epicardial cells, supporting thus functional implications for the development of both the proepicardium and the embryonic epicardium. Moreover, the inhibition *Foxf1* modulates *let-7c,* promoting the expression of early and terminal cardiogenic lineage markers in epicardial cells. We demonstrate that differentially expressed *Foxf1* transcription factor exerts regulatory control over *miR-495* and *let-7c*, thereby modulating epicardial cell migration and myocardial specification, underscoring the intricate interplay between transcription factors and microRNAs in governing cardiogenesis.

Funding: This work was supported by grants of the Ministerio de Innovación y Ciencia of the Spanish Government to DF (PID2022-138163OB-C32) and of the Consejería de Universidad, Investigación e Innovación of the Junta de Andalucia Regional Council to DF (ProyExcel_00409).

P_23

Identification of an NKX2-5-dosage dependent progenitor cell population contributing to the Purkinje fibre network

Louise Michel, Gaetano D'Amato, Rachel Sturny, Nicolas Bertrand, Robert G. Kelly, Lucile Miquerol

Aix-Marseille University, CNRS UMR7288, IBDM, Campus de Luminy, Marseille, France

Background: The Purkinje fiber network (PFN) allows fast conduction of the electrical impulse through the ventricles. Defects in the PFN participates in the triggering and/or maintenance of ventricular arrythmias that can lead to sudden cardiac death. A better characterization of the development and maturation of the PFN leading to its complex ellipsoid architecture can help us understand the appearance of such defects. During development, PF recruitment is dependent on the expression level of the transcription factor NKX2-5, and *Nkx2-5* haploinsufficiency leads to a hypoplastic PFN. A preliminary unpublished single cell transcriptomic dataset from embryonic hearts generated in the lab has identified a cardiomyocytes (CM) progenitor cell population that is absent in *Nkx2-5* heterozygous hearts. Our study aims to characterize this novel population and determine the impact of *Nkx2-5* haploinsufficiency leading to architectural defects of the PFN.

Methods: Genetic tracing of cells expressing Lysozyme M (*Lyz2*) was performed at different stages using *Lyz2-Cre* mice crossed with the *Rosa26-TdTomato* reporter line. The Lyz2 lineage contribution to the VCS was established by whole-mount imaging of cleared embryonic hearts and open adult left ventricles from WT and *Nkx2-5* haploinsufficient mice (*Nkx2-5*$^{+/-}$).

Results and Conclusion: Single cell RNA-seq analysis identifies unbalanced trabecular clusters between WT and *Nkx2-5*$^{+/-}$ hearts at E11.5. In particular, a sub-population of trabecular CMs absent in *Nkx2-5*$^{+/-}$ hearts shows a progenitor like-cell transcriptomic profile and expresses *Lyz2*. Analysis of adult hearts reveals that the *Lyz2* genetic lineage contributes to the VCS. *Lyz2* lineage labelling is indistinguishable in *Nkx2-5*$^{+/-}$ and wildtype hearts at E14.5 but strikingly does not contribute to the PFN in *Nkx2-5*$^{+/-}$ hearts. Our results suggest that the *Lyz2* lineage makes a late contribution to the VCS which is dependent on maximal Nkx2-5 levels.

Funding: Fondation pour la recherche Médicale (FRM)

P_24

Spatiotemporal Expression of SOX6, SOX7, and SOX9 in Valvuloseptal Development with a focus on SOX6 in the Outflow Tract

Hannah Tarolli[1], Ray Deepe[1], Inara Devji[1], Jenna Drummond[1], Andrew Harvey[1], Renélyn Wolters[1,2], Andy Wessels[1]

[1] *Medical University of South Carolina, Charleston, SC*

[2] *University of Tokyo, Tokyo, Japan*

Background: The mesenchymal structures that contribute to the valves in the outflow tract (OFT) and atrioventricular (AV) junction play a critical role in cardiovascular development. These structures rely heavily on the contribution of several cell lineages. The Second Heart Field (SHF) derived cells, cardiac neural crest cells (CNCCs), and endocardial-derived cells (ENDCs) contribute to the mesenchyme of the OFT cushions (OFTCs), while the intercalated ridges (ICRs) in the OFT are almost completely comprised of SHF cells. The major and lateral AV cushions (AVCs) initially only become populated by ENDCs, but at later stages, epicardial-derived cells (EPDCs) start to invade the lateral AVCs as well. Numerous transcriptional regulatory mechanisms are described as influencing how the respective cell populations contribute to the heart. In ongoing studies in the lab, we investigate the role of SOX9 in the SHF and EPDCs. These studies have led to a widening of our interest into the role of other SOX family members, including SOX6 and SOX7, in the context of valvuloseptal morphogenesis.

Methods: We use mouse model systems and immunohistochemical methods to better understand the lineage-specific distribution of SOX6, SOX7, and SOX9 expressing cells. To specifically pursue the role of SOX6 in heart development, a Sox6$^{fl/fl}$ mouse is used in combination with the cre-recombinase models to conditionally delete Sox6 in cell lineages of interest.

Results and Conclusion: The respective spatiotemporal expression patterns of the SOX variants suggest that each SOX family member may be playing a unique cell-lineage associated role in valvuloseptal development. Preliminary analysis of mouse embryos in which Sox6 is deleted in a cell-lineage specific manner indicates that SOX6 has an important role in cardiac morphogenesis.

Funding: National Institute of Health; T32GM132055-03 (HT), RO1HL122906 (AW), RO1HL162913 (AW), T32-HL007260 (AH) and American Heart Association; AHA23PRE1014420 (AH)

P_25

Determining who does what (…and how) in the zebrafish cardiac pacemaker

Federico Tessadori[1], Lysander Blankenborg[1], Laurence Garric[1], Olga Veth[1] and Jeroen Bakkers[1,2]

[1] *Hubrecht Institute-KNAW and University Medical Center Utrecht, Utrecht, The Netherlands*

[2] *Department of Pediatric Cardiology, Division of Pediatrics, University Medical Center Utrecht, Utrecht, The Netherlands*

Background: While the heart shape, relative size and chamber number varies across vertebrate species, the rhythmicity and origin of cardiac contractions is evolutionarily conserved. In higher vertebrates such as mammals, specialized pacemaker cells controlling the rhythmicity of cardiac contractions are localized in an anatomically identifiable structure of myocardial origin, the sinoatrial node (SAN). Previously, we have shown that this same functional pacemaker unit has a ring-like organization in a lower vertebrate such the zebrafish. Pivotal to this finding was the identification of the LIM/homeodomain-containing transcription factor Islet-1 (Isl1) as the marker for zebrafish cardiac pacemaker activity.

Methods: We have carried out single-cell RNA sequencing on zebrafish embryonic cardiomyocytes and have identified factors co-expressed with *isl1* as well as transcription regulators potentially playing a role in regulating its expression in pacemaker cells. In parallel, we are assessing the genetic interaction between Isl1 and other specific transcription factors with an established importance in the development of the vertebrate heart such as Tbx5, Nkx2.5 and Shox2*,* amongst others. Their role in defining -or not- pacemaker identity is assessed *in vivo* in a dedicated zebrafish transgenic reporter line. Our approach combines the use of single and combined loss-of-function zebrafish models, transgenic pacemaker identity reporters and double-allelic targeting of genes of interest.

Results and Conclusion: Our systematic dissection of the activity of cardiac effectors allows the analysis of their effect on pacemaker patterning and function. For instance, we have determined that the expression of Shox2 is tightly dependent on Isl1 at the venous pole, and have assessed its importance for the establishment of pacemaker function. Our data suggest that while genetic factors and cellular processes underlying cardiac development and function are conserved, their individual role and the position they occupy in the intricate hierarchy leading to proper cardiac development and function differs across species.

Funding: Dutch Research Council (NWO): Grant NWO/OCENW.GROOT.2019.029

P_26

Noncoding regulation of epicardial EMT during heart development

Mariana Valenzuela Sanchez[1], Carolina Roque Silva[1], Hannah Scheucher[1], Tiago Dias[1], Jacinta Kalisch-Smith[2], Duncan Sparrow[2], Paul Riley[2], Andia Redpath[2], Joaquim Nunes Vieira[2]

[1] *School of Cardiovascular and Metabolic Medicine & Sciences, King's College London, London, United Kingdom*
[2] *Institute of Developmental & Regenerative Medicine - Department of Physiology, Anatomy & Genetics, University of Oxford, Oxford, United Kingdom*

Background: During organogenesis, cells from the outer layer of the heart, the epicardium, undergo epithelial-to-mesenchymal transition (EMT), contributing essential paracrine signals and cells to the growing heart. Epicardial cells are integral to heart regeneration in lower vertebrates and neonatal mammalian injured hearts. Cell fate decisions underpinning EMT are directed by transcription factors such as Wilms' tumour 1 (WT1). Whilst a requirement for *Wt1* in heart development is widely accepted, the upstream regulatory mechanisms underpinning its activation remain elusive. We identified two intronic evolutionary conserved regions (ECRs) shared between mouse and human, located within intron 1 of the *Wt1* locus, and hypothesised that ECRs direct locus activation to support EMT.

Methods: We used CRISPR/Cas9 gene-editing to generate $Wt1^{\Delta ECR}$ mice carrying a sequence deletion containing one or both ECRs. Extensive survival analysis, high-resolution episcopic microscopy (HREM), qPCR, immunostaining, confocal microscopy and epicardial explants were used to characterise heart formation/EMT.

Results & Conclusion: HREM revealed smaller hearts, incidence of myocardial non-compaction and spongy interventricular septum with muscular or membranous ventricular septum defects, tricuspid hypoplasia and enlarged aortic valves in $Wt1^{\Delta ECR/\Delta ECR}$ hearts. Expression of *Wt1* was markedly reduced in mutant hearts, but not in the kidneys, suggesting intronic enhancers are cardiac-specific. Moreover, immunostaining studies revealed abnormal coronary vessel and innervation extension and patterning in the subepicardium, as well as reduced epicardial EMT in $Wt1^{\Delta ECR/\Delta ECR}$.

Conclusion: We demonstrated a requirement for novel *Wt1* intronic enhancers regulating locus activity and impacting on essential epicardial EMT and associated biological processes during normal heart development. Importantly, observation of septum and semilunar valve defects in our ΔECR mutants suggests an hitherto unrecognised role for WT1 and epicardial EMT and opens new avenues of research to improve our understanding of congenital heart disease that affects at least 1:150 live births, with remarkably two thirds of cases having unknown aetiology.

Funding: BHF Fellowship FS/19/31/34158.

P_27

BAF155 regulates the development of intercalated valve swelling (ICVS) participating in cardiac outflow tract septation

Haiyue Feng[1], Hengwei Jin[2], Jie Yao[1], Lijun Zhang[1], Xiaodong Wang[2], Zhongzhou Yang[1]

[1] *Nanjing University Medical School, Nanjing, 210093, China*
[2] *Model Animal Research Center, Nanjing University Medical School, Nanjing, 210061, China*

Background: Previous studies show that the intercalated valve swelling (ICVS) is derived from the *Tnnt2-Cre*+/Isl1+ progenitors in the outflow tract (OFT) wall, which is dependent on Notch-Jag signaling pathway. Here, we are aimed to uncover the novel regulatory insights in ICVS formation.

Methods: Using mouse genetics together with single-cell sequencing, we investigated the new insights in regulating ICVS development and to study its involvement in OFT septation.

Results and Conclusion: We uncovered that the ICVS cells were distinguishable at E9.5. These cells are both Nkx2-5-Cre and Mef2c-Cre positive. The ICVS cells of the prospective pulmonary artery express Isl1, Nkx2-5, Hand2 and Mef2c. We identified Baf155 expression in the ICVS cells and found that ablation of *Baf155* in the second heart field (SHF) using both Nkx2-5-Cre and Mef2c-Cre resulted in expansion of the ICVS. The Baf155 mutant mice showed persistent truncus arteriosus (PTA) probably as a result of main cushion malformation. Thus, we have discovered the new molecule regulating ICVS development and revealed a possible novel function of ICVS in OFT septation.

Funding: Supported by the Natural Science Foundation of China and the key R&D program of China.

P_28

GPAT4 sustains endoplasmic reticulum homeostasis in endocardial cells and safeguards heart development

Tianyang Zhao[1], Kuipei Jin[2], Xiaodong Wang[1], Zhongzhou Yang[1&]

[1] State Key Laboratory of Pharmaceutical Biotechnology, MOE Key Laboratory of Model Animal for Disease Study, Model Animal Research Center, and Jiangsu Key Laboratory of Molecular Medicine, Nanjing University Medical School, Nanjing 210093, China

[2] Laboratory of Lipid Metabolism, Department of Biochemistry and Molecular Biology, Key Laboratory of Neural and Vascular Biology, Ministry of Education, Key Laboratory of Medical Biotechnology of Hebei Province, Cardiovascular Medical Science Center, Hebei Medical University, Shijiazhuang, Hebei 050017, China

Background: The endocardium plays a pivotal role in governing myocardial development, and understanding the intrinsic regulatory insights will help apprehend pathological cardiomyopathy. Glycerol-3-phosphate acyltransferase 4 (GPAT4) is an endoplasmic reticulum (ER) membrane anchored protein. While the role of GPAT4 in glycerophospholipid biosynthesis is well established, its function in the ER is less explored.

Methods: We used 3 global Konckout (*Gpat4/Ifnar1/Sting*) and 4 conditional knockout (*Gpat4 fl/fl* with *Tie2-Cre/Nfatc1-Cre/Mef2c-Cre/cTnt-Cre*) mice, 2 cell lines (HUVECs and H9c2). And RNA-seq, lipidomics, IP-MS, WB, qPCR, IF.

Results and Conclusion: We generated *Gpat4* global and tissue-specific knockout mice and identified the essential role of GPAT4 in endocardial development. Deficiency of GPAT4 provoked endocardial ER stress response and enhanced ER-mitochondrial (ER-mito) communications, leading to mitochondrial calcium overloading and escape of mitochondrial DNA (mtDNA). As a result, the cGAS-STING pathway was trigged to stimulate type I interferon response, which affected endocardial and myocardial development. Finally, abolishment of the cGAS-STING-type I interferon pathway could rescue the heart defects of *Gpat4* deletion mice. These findings uncovered the pivotal role of GPAT4 in the maintenance of ER homeostasis and in fine-tuning ER-Mito contacts during endocardial and heart development. Meanwhile, this study highlights the importance of cGAS-STING pathway in cardiac organogenesis.

Funding: Supported by Postgraduate Research＆Practice Innovation Program of Jiangsu Province, CHINA.

Poster Session 3 – Cardiac Regeneration

Operating on the life science market **since 2004**, Animalab is a well-coordinated team of specialists interested in various areas of life sciences. Their extensive experience in the industry enables the company to offer advice and comprehensive service in Poland, Czechia, Slovakia, Hungary, Lithuania, Latvia, Estonia, Croatia and Slovenia. With the opening of the Germany location in 2022, AnimaLab is expanding the network again and serving growing needs in the European market.

https://animalab.eu

P_29

ZebraReg – a novel platform for discovering regulators of cardiac regeneration using zebrafish

Kateřina Apolínová [1,2], Ferran Arqué Pérez [2,3], Sylvia Dyballa [3], Benedetta Coppe [4,5], Nadia Mercader Huber [4,5,6], Javier Terriente [3*†], Vincenzo Di Donato [1*†]

[1] *ZeClinics, Sant Feliu de Llobregat, Barcelona, Spain*
[2] *Biomedicine, Department of Medicine and Life Sciences, Faculty of Health and Life Sciences, Pompeu Fabra University, Barcelona, Spain*
[3] *ZeCardio Therapeutics, Sant Feliu de Llobregat, Barcelona, Spain*
[4] *Developmental Biology and Regeneration, Institute of Anatomy, University of Bern, Bern, Switzerland*
[5] *Department for Biomedical Research DBMR, University of Bern, Bern, Switzerland*
[6] *Centro Nacional de Investigaciones Cardiovasculares CNIC, Madrid, Spain*

†These authors have contributed equally to this work and share last authorship.

Background: Cardiovascular disease is the leading cause of death worldwide with myocardial infarction being the most prevalent. Adult mammalian hearts display a limited regeneration capacity, but it is insufficient to allow complete myocardial recovery after the massive death of cardiomyocytes. In contrast, the injured zebrafish heart muscle regenerates efficiently through robust proliferation of pre-existing myocardial cells. Thus, zebrafish allows its exploitation for studying the genetic programs behind cardiac regeneration, which may be present, albeit dormant, in the adult human heart.

Methods: We introduce ZebraReg, a novel, versatile method that combines genetic and pharmacological techniques to achieve the ablation of a specific sub-population of ventricular cardiomyocytes in zebrafish larvae. In combination with an automated medium-throughput imaging-based method, heart regeneration kinetics can be determined through a longitudinal analysis of the regenerative process.

Results and Conclusion: We demonstrate that the platform can be integrated with genetic or pharmacological approaches and used for medium-throughput screening of presumed modulators of heart regeneration. The platform is versatile and can identify both anti- and pro-regenerative effects of both genes and drugs. In conclusion, we present a tool which may be utilised to streamline the process of target validation of novel gene regulators of regeneration, and the discovery of new drug therapies to regenerate the heart after myocardial infarction.

Funding: European Union's Horizon 2020 Framework Programme for Research and Innovation under grant agreement No. 874764 (REANIMA 2020) and European Union's Horizon 2020 Innovative Training Network under grant agreement No. 861329 (SCilS).

https://www.zecardiotherapeutics.com/

P_30

Matters of the heart: lizards as models for myocardial regeneration

Martina Gregorovicova[1], Barbora Sankova[1], Martin Bartos[1,2], Bjarke Jensen[3], Tobias Wang[4], David Sedmera[1]

[1] *Institute of Anatomy, First Faculty of Medicine, Charles University, U Nemocnice 3, 128 00 Prague 2, Czech Republic*

[2] *Institute of Dental Medicine, First Faculty of Medicine,Charles University, Katerinska 32, 121 08 Prague, Czech Republic*

[3] *Department of Medical Biology, Amsterdam Cardiovascular Sciences,*
University of Amsterdam, Meibergdreef 9, 1105 AZ Amsterdam, The Netherlands

[4] *Department of Zoophysiology, Aarhus Universitet, C. F. Mollers Alle 3, 8000 Aarhus C, Denmark*

Background: Reptilian myocardial regeneration and its mechanisms are still elusive. Squamates could fill knowledge gap between traditional poikilothermic anamnian (zebrafish) and homeothermic placental (neonatal mammals) models and thus could reveal myocardial regenerative mechanisms not only in reptiles but also in amniotes. Factors, potentially influencing myocardial regenerative potential, are: i) ventricular septation, and ii) taxon-specific regenerative abilities (reflected in metabolism, ecological niche, and phylogeny). Therefore, the crux of the problem is what is the relationship among regenerative potential and those factors.

Methods: Cryoinjury was performed on heart apex in two lizard species (*Eublepharis macularius*, and *Varanus acanthurus*) chosen for opposite life-histories and phylogenetic positions. After various healing intervals, physiological (from *in-vitro* optical mapping) and morphological parameters (from microCT, histology and immunohistochemistry) were used for evaluating myocardial regeneration.

Results and Conclusion: Physiologically, heart rate differed significantly between *Eublepharis* and *Varanus*. Morphologically, recovery period was divided into i) initiating period with macrophages infiltration (first week), ii) fibrotic scar formation (second week), and iii) fibrotic scar resolution and its myocardial replacement (third week). There was no fibrotic scar after three weeks healing period in tested species but species differed in regenerative velocity. *Varanus* was ahead of *Eublepharis* and replaced scar during second week, *Eublepharis* in third one. *Eublepharis* does not have good ventricular septation and together with nocturnal life-style, this could lead to slower cardiac regeneration compared to *Varanus*. Observations are connected to different metabolic rates and thus to different life-histories and ecological niche. The role of the position in the phylogenetic tree is not excluded but not confirmed yet. Faster regeneration in *Varanus* could be explained by high metabolic rate, which goes against the results known from homeothermic studies.

Funding: Supported by Ministry of Education, Youth and Sports, Czech Republic: Progres Q38/1LF, Progres Q29/1LF, Cooperatio 207029 Cardiovascular Sciences, and Cooperatio 207036 Morphological Sciences

P_31

Cardiomyocyte dynamics in the regenerating zebrafish heart

Laura Peces-Barba-Castano, Gamze Aydiner, Miloslav Sanda, Kenny Mattonet, Didier Stainier

Department of Developmental Genetics, Max Planck Institute for Heart and Lung Research, Bad Nauheim, Germany

Background: Cardiac diseases are a leading cause of death worldwide as mammals cannot regenerate their heart beyond the neonatal stages, one of the reasons being insufficient cardiomyocyte (CM) renewal. Regenerating CMs go through dedifferentiation, proliferation and redifferentiation to replace lost CMs. Here, we used the cryoinjury model to study heart regeneration in zebrafish, a regenerative organism. In zebrafish, the peaks for CM dedifferentiation and proliferation are situated at 96 hours post cryoinjury (hpci) and 7 days post cryoinjury (dpci). Nevertheless, the main proteins and phosphorylated targets preceding these timepoints as well as the dynamics of these processes are yet to be fully identified and analysed.

Methods: We extracted cryoinjured hearts at 78 and 150 hpci, which we separated into remote area and borderzone-injury area, as well as untouched hearts. These samples were submitted to mass spectrometry to investigate the protein and phosphosite enrichment in each region and timepoint.

To analyse CM dynamics during regeneration, we extracted hearts at different timepoints: 6, 24, 48, 72, 96 and 120 hpci, as well as 7, 9, 11 and 13 dpci, along with untouched hearts. These samples are being immunostained to characterise how cell adhesion, sarcomere disassembly, CM dedifferentiation and proliferation fluctuate over time. For now, the analysis of the slices has been automated by developing a macro for FiJi.

Conclusion: The protein and phosphoprotein profiles are most similar between remote and untouched and most different between borderzone-injury and untouched. The remote 150 hpci condition was most similar to untouched while the 150 hpci borderzone-injury was most different. The main changes involve metabolic pathways, protein synthesis and DNA replication. The second project aims to provide a useful timeline of the changes in regenerating CMs, to aid in choosing timepoints and characterizing phenotypes in mutant zebrafish or after pharmacological treatments.

Funding: Max Planck Society

Developmental Genetics (Dept. III) | Max Planck Institute for Heart and Lung Research - W. G. Kerckhoff Institute (mpi-hlr.de)

P_32

The ion channel Trpc6a regulates the cardiomyocyte regenerative response to mechanical stretch

Laura Rolland, Jourdano Mancilla Abaroa, Adèle Faucherre, Aurélien Drouard, Chris Jopling✉

Institute of Functional Genomics, University of Montpellier, CNRS, INSERM, LabEx ICST, 141 rue de la Cardonille, 34000 Montpellier, France.

Background: Myocardial damage caused, for example, by cardiac ischemia leads to ventricular volume overload resulting in increased stretch of the remaining myocardium. In adult mammals, these changes trigger an adaptive cardiomyocyte hypertrophic response which, if the damage is extensive, will ultimately lead to pathological hypertrophy and heart failure. Conversely, in response to extensive myocardial damage, cardiomyocytes in the adult zebrafish heart and neonatal mice proliferate and completely regenerate the damaged myocardium. We therefore hypothesized that in adult zebrafish, changes in mechanical loading due to myocardial damage may act as a trigger to induce cardiac regeneration. Based, on this notion we sought to identify mechanosensors which could be involved in detecting changes in mechanical loading and triggering regeneration.

Methods: Here we show using a combination of knockout animals, RNAseq and in vitro assays that the mechanosensitive ion channel Trpc6a is required by cardiomyocytes for successful cardiac regeneration in adult zebrafish. Furthermore, using a cyclic cell stretch assay, we have determined that Trpc6a induces the expression of components of the AP1 transcription complex in response to mechanical stretch

Results and Conclusion: Our data highlights how changes in mechanical forces due to myocardial damage can be detected by mechanosensors which in turn can trigger cardiac regeneration.

Funding: The Jopling lab is part of the Laboratory of Excellence Ion Channel Science and Therapeutics supported by a grant from the ANR. Work in the Jopling lab is supported by a grant from the “la Fondation Leducq” and from the ANR (contract ANR-20-CE14-003-02 MetabOx-Heart and ANR-22-CE14-048-02 IONIC).

Hyperlink: https://doi.org/10.3389/fcvm.2023.1186086

https://www.igf.cnrs.fr/index.php/fr/h-teams-fr/ht-jopling-fr

P_33

Septin15 is required for cardiac regeneration after cryoinjury in adult zebrafish

Suneeta Narumanchi[1], Sanni Perttunen[1], Katariina Immonen[1], Ilkka Tikkanen[1,2], Päivi Lakkisto [1,3], Jere Paavola[1]

[1] *Unit of Cardiovascular Research, Minerva Institute for Medical Research, Biomedicum Helsinki, Helsinki, Finland*

[2] *Abdominal Centre, Nephrology, University of Helsinki and Helsinki University Hospital, Helsinki, Finland*

[3] *Clinical Chemistry and Hematology, University of Helsinki and Helsinki University Hospital, 00014 Helsinki, Finland*

Background: Septins are a family of GTP-binding proteins that are associated with actins. Septin15 is expressed in embryonic and adult zebrafish heart. However, the role of septin15 in cardiac function and regeneration in adult zebrafish is not known.

Methods: Septin15 ENU Knockout (KO) embryos were obtained from sanger's institute (sept15sa10940) and F3 generation adults were used for this study. Cryoinjury, a procedure in which the apex of the ventricle was cooled with a metal probe dipped in liquid nitrogen, was utilized to mimic myocardial infarction in adult zebrafish. Heart samples were collected at 7 days post injury (7dpi), 14dpi and 21dpi for qPCR, histology and immunohistochemistry. Acid Fuchsin Orange G staining (AFOG) was utilized to measure fibrin and collagen accumulation. Immunohistochemistry with proliferating cell nuclear antigen (PCNA) was used to count proliferating cardiomyocytes. qPCR with Plasminogen activator inhibitor-1 (PAI-1) primers was performed.

Results and Conclusion: We found that septin15 KO fish exhibited lower cardiac proliferation at 7 dpi ($p = 0.01$) and increased fibrin/collagen ratio at 21 dpi compared to wildtype (wt) fish (P=7,807E-05). Additionally, at 14dpi, expression of PAI-1, a serine protease inhibitor which inhibits fibrinolysis is more in KO fish when compared to wt (P= 0,025). These results indicate that lack of septin15 is detrimental for cardiac regeneration after cryoinjury in adult zebrafish.

Funding: This research was supported by grants from Finnish Cultural Foundation, Alfred Kordelinin Säätiö, Aarne Koskelon Säätiö, Idamontinin Säätiö and Sydääntutkimussäätiö.

P_34

Single cell transcriptomics of adult mononucleated cardiomyocytes reveals a single transcription factor that regulates CM polyploidization in mammalian hearts

Villa del Campo C., Rivero I., Sierra R., Torres M.,

Cardiovascular Regeneration Program, Centro Nacional de Investigaciones Cardiovasculares (CNIC), Madrid, 28029, Spain

The mammalian heart is a non-regenerative organ due to the almost complete inability of differentiated CMs to proliferate. While neonatal hearts (similarly to lower vertebrates), still contain mostly diploid CMs and regenerate efficiently; physiological postnatal CM polyploidization represents a roadblock to cardiac regeneration. Due to this limitation, diseases that concur with CM loss frequently lead to heart failure. Moreover, in mice, the proportion of mononuclear adult diploid CMs correlates with regenerative ability. It is therefore suggested that these diploid cardiomyocytes can constitute a specific population that carries potential regenerative ability. However, efforts to identify a molecular signature for mononuclear diploid CMs have been unsuccessful, precluding the exploration of their potential for heart regeneration.

Using an improved methodology established in-house for single-CM RNAseq, we have identified a molecular signature of mononuclear diploid cardiomyocytes related to the fetal program and controlled by a single repressor transcription factor. This factor is specifically expressed in the polyploid adult CM population and its inhibition in the postnatal mouse heart increases the abundance of a mononuclear population of cardiomyocytes.

Based on these findings, we have studied whether promoting a boost in the number of mononucleated cardiomyocytes through this factor invokes a regenerative response and the physiological consequences such a mechanism entails.

We are currently exploring the regulatory network that modulates the generation of mononuclear cardiomyocytes and identifying this population in the heart in homeostatic conditions in order to promote their renewal and expansion during aging and disease.

Unravelling these mechanisms and developing ways to target mononuclear cardiomyocytes will enable us to establish new strategies to promote heart regeneration based on the reactivation of an endogenous mechanism, achieving unprecedented advances for cardiac regenerative strategies.

LIFE SCIENCE AND MEDICAL SUPPLIES

Laboratory plastics, cell and tissue culture supplies and other consumables
Serving the Czech Republic, Slovakia and nearby regions

https://www.lifem.cz

P_35

Investigating the role of the extracellular matrix during cardiac valve development

Ritvik Gupta, Agatha Ribeiro da Silva, Thomas Juan, Didier Y.R. Stainier

Max Planck Institute for Heart and Lung Research, Bad Nauheim, Germany

Background: Cardiac valves are essential for cardiac function as they prevent retrograde blood flow, and valve malformation can lead to heart failure. During valve development, the extracellular matrix (ECM) supports valve elongation. However, the ECM factors essential for valve elongation remain poorly described. Recently, we identified the transcription factor *Egr3* as a major regulator of valve elongation in zebrafish, with valve cells failing to invade the ECM in mutants. Suggesting that *Egr3* targets are essential factors to control ECM interaction with valve cells.

Methods: We investigated the role of *nrg1* and *spp1*, both of which are *Egr3* targets specifically expressed in the valve. We generated large deletions of the coding region of these two genes using a CRISPR/Cas9 approach to investigate their role in valve elongation. We also confirmed their valve-specific expression pattern using transcriptomic profiling and *in-situ* hybridization and are currently developing knock-in strategies to visualize their expression *in-vivo*.

Results and Conclusion: The analysis of *nrg1* expression, which is present in only half of the valve cells, suggests that cardiac valves are heterogeneous before the onset of valve elongation. We also identified *rspo2* as a distinct valve marker for the *nrg1*-negative cells and are developing knock-in tools to investigate the origin and function of these populations. Overall, we expect to gain an understanding of ECM interaction with endocardial cells during valve development through the study of these candidate genes.

Funding: IMPRS, MOB and Max Planck Society

P_36

A novel Smad6 knockout mouse model: bicuspid aortic valve and outflow tract-related pathology

Ilse Luyckx[1,2], Bert Wisse[3], Tamara Borsboom[3], Jarl Bastianen[1], Marie-José Goumans[4], Marco DeRuiter[3], Bart Loeys[1,2]

[1] *Centre of Medical Genetics, Faculty of Medicine and Health Sciences, University of Antwerp and Antwerp University Hospital, Prins Boudewijnlaan 43, 2650 Edegem, Belgium*

[2] *Department of Clinical Genetics, Radboud Univeristy Medical Center, Geert Grooteplein Zuid 10, 6525 GA Nijmegen, The Netherlands*

[3] *Department of Anatomy and Embryology, Leiden University Medical Center, Albinusdreef 2, 2333 ZA Leiden, The Netherlands*

[4] *Department of Cell and Chemical Biology, Leiden University Medical Center, Albinusdreef 2, 2333 ZA Leiden, The Netherlands*

Background: Bicuspid aortic valve-related aortopathy (BAV/TAA) is a serious health problem. New pathomechanistic insights are highly needed to identify early predictive biomarkers to recognize BAV individuals at-risk for TAA. To address this, we developed and characterized a novel mouse model lacking the most common known genetic factor implicated in human BAV/TAA disease, i.e. *SMAD6*. Cardiovascular pathology in mutant adults included BAV, aortic valve stenosis, and thoracic aortic wall pathology (unpublished data). At present, we study the effect of Smad6 loss on the development of outflow tract disease.

Methods: Adult *Smad6* knockout mice were bred overnight. Embryos were collected and prepared for analyses. Images were taken with a Leica S9i (Leica) and a Pannoramic 250 Flash III slide scanner (3DHISTECH Ltd). Only statistical significant results are described ((two-tailed) Fisher's exact test).

Results and Conclusion: Analysis of progeny of Smad6$^{+/-}$ intercrosses demonstrates postnatal lethality of Smad6$^{-/-}$ mice without embryonic loss. Macroscopic analysis of E17.5 Smad6$^{-/-}$ embryos (N=5/6, 83%, p=0,003) illustrates nuchal translucency, petechia, delayed digit formation and smaller embryos. Histological cardiovascular characterization revealed structural defects including bicuspid aortic valve (N=7/7, 100%, p=0,0006) and septal defects (N=2/7, 29%) as most obvious anomalies in Smad6$^{-/-}$ embryos (E15.5, E17.5). In Smad6$^{-/+}$ littermates, aortic valve malformation (N=2/6, 33%) was the most prevalent structural anomaly, followed by a huge diameter of the pulmonary right artery and trunk (N=1/6, 17%). In addition to structural defects, an abnormal rotation of the outflow tract in Smad6$^{-/-}$ embryos (E13.5) leads to a more lateral position of the aorta, which keeps the aorta highly connected with the right ventricle, and with only a very small connection to the left ventricle. Altogether, our Smad6 knockout mouse model is a high penetrant model for bicuspid aortic valve, associated with other outflow tract-related pathologies affecting the pulmonary artery and aorta.

Funding: This research was supported by funding from the University of Antwerp (Methusalem-OEC grant "Genomed" FFB190208; KP-BOF 41611) and by the Fonds Hartchirurgie. B.L. holds a consolidator grant from the European Research Council (Genomia – ERC-COG-2017-771945). B.L. is a member of the European Reference Network on rare multisystemic vascular disorders (VASCERN - project ID: 769036 partly co-funded by the European Union Third Health Programme). I.L. was an ERN fellowship (EJP RD WP17). She is supported by the Outreach project (Dutch Heart Foundation) and TKI-LSH Health Holland.

P_37

Mechanisms of age-related cardiac valve calcification in zebrafish *αklotho* mutants

Nuno Valério Santos-Silva[1,2], Luisamaria C. Matiz[1], Bárbara Gonçalves-Cardoso[1], Clémentine Decamps[1], Catherine Robert[4], Camille Humbert[4], Inês Cristo[3], Stephane Zaffran[4], Anabela Bensimon-Brito[1]

[1] *Molecular, Cellular and Developmental Biology (MCD), UMR5077, Centre de Biologie Intégrative (CBI), Université de Toulouse/CNRS, Toulouse, France*

[2] *Aix Marseille University, École Doctorale Sciences de la Vie et de la Santé (EDVS62), Marseille, France*

[3] *CCUL@RISE – Centro Cardiovascular da Universidade de Lisboa, Faculdade de Medicina da Universidade de Lisboa, Portugal*

[4] *Aix Marseille University, Marseille Medical Genetics - U 1251 INSERM – Marseille, France*

Background: Cardiovascular calcification (CVC) is linked with progressive dysfunction of major arteries and cardiac valves due to soft tissue calcification. Ageing is an important risk factor that promotes both vascular and valvular calcification. α-Klotho is an anti-ageing hormone, and its loss is sufficient to induce premature ageing phenotypes, leading to increased morbidity and mortality. Interestingly, zebrafish and mouse α-Klotho mutants present extensive vascular calcification in the outflow tract (OFT) and major arteries, respectively. Although there are reports showing cardiac valve calcification in mouse α-Klotho mutants, no models for valve calcification have been described in zebrafish. We aim to establish a zebrafish model for valve calcification, and elucidate the cellular heterogeneity and molecular mechanisms underlying age-related cardiovascular calcification.

Methods: We have generated a zebrafish *αklotho* mutant to determine the presence of calcification in both the OFT and cardiac valves. For that purpose, we used immunohistochemistry and bone staining techniques combined with imaging analysis and single cell RNA sequencing (sc-RNAseq) approaches.

Results and Conclusion: Characterization of adult hearts from *αklotho* mutants showed that cardiac valves undergo calcification, and that this occurs after OFT calcification. This is, to our knowledge, the first model of cardiac valve calcification in zebrafish, opening important avenues in the field. Immunostaining data in calcified heart sections for multiple ECM components and pan-immune cell markers suggested alterations of the cardiac valve ECM organization and possible increase in the inflammatory status. We are now using sc-RNAseq approaches to determine valve cell heterogeneity that may be linked to the onset of valve calcification. With this project, we expect to provide a unique understanding of the cell heterogeneity and susceptibility to ageing in cardiovascular tissues, highlighting the features that make cells and tissues prone to both vascular and valvular calcification.

Funding: Supported by the European Research Council, StG #101042865, by the Institut National de la Santé et de la Recherche Médicale (Inserm), including the Atip-Avenir Program, by the Centre national de la recherche scientifique (CNRS), and by the Center for Integrative Biology (CBI) - Molecular, Cellular and Developmental Biology (MCD).

Hyperlink: https://cbi-toulouse.fr/fr/equipe-bensimon-brito

P_38

BMP-Notch signaling interaction in endocardial cushion mesenchymal cells plays critical roles in AV valvulogenesis and valve disease progression

Patrick G. Smith[1,2], Miriam M. Atteya[1,2], Maria G. Gonzalez[1,2], Haleigh Ferro[1,2], Kazuaki Maruyama[3], Kyoko Imanaka-Yoshida[3] , Yukiko Sugi[1,3]

[1] *Department of Regenerative Medicine and Cell Biology, Medical University of South Carolina, Charleston, SC 29425, USA*

[2] *College of Charleston Honor's College, Charleston, SC 29425, USA*

[3] *Department of Pathology and Matrix Biology, Graduate School of Medicine, Mie University, Tsu, Mie 514-8507, Japan*

Endocardial cushions in the atrioventricular (AV) canal undergo maturation and remodeling into a ventricular membranous septum and AV valves. Because both BMP2 and Notch2 are expressed in the AV cushion mesenchyme, we have tested our hypothesis that BMP-Notch interaction is critical for AV valvulogenesis and valve disease progression. In this study, we used AV cushion mesenchymal cell cultures and genetically engineered mouse models. In our *in vitro* culture assays, we found that a BMP signaling intermediate, Smad1 interacts with a Notch pathway component, Notch2 intracellular domain (ICD) in the nuclei of AV cushion cells and that BMP2 induces a Notch pathway effector, *Hey1* in the cushion cells. In our *in vivo* studies, we assessed the effect of combining up-regulated BMP signaling and down regulated Notch signaling by generating double mutant, ca*Alk3; Rbpj*$^{flox/+}$; *Nfatc1*^{Cre} mice. The double mutant mice exhibited degenerative phenotypes and aberrant deposition of extracellular matrix (ECM) components in the anterior leaflet of mitral AV valves at 8 weeks old. Echocardiography revealed mitral valve prolapse and regurgitation in the double mutant mice. Because it is intriguing that mature valve cusps/leaflets are known to frequently exhibit many of the same molecules present in the chondrogenic and osteogenic lineages, we further explored the potential that AV cushion mesenchymal cells from our double mutant mice can differentiate into chondrogenic and/or osteogenic lineages. Although a known osteogenic marker *Runx2* nor mature chondrogenic markers were not exhibited in the valve leaflets, expression of early chondrogenic markers, *Sox9* and *Aggrecan* was induced in the anterior leaflet of mitral valves in the double mutant mice. We also found clusters of chondrocytes at the aortic leaflet-wall junction. However, lineage tracing with *Nfatc1*^{Cre} did not reveal evidence that these chondrocytes are derived from an endocardial lineage. Potential interactions between endocardial and other lineages such as neural crest derive cells in our double mutant mice are of interest.

Supported by AHA 18TPA34179356 and NIH/NIGM P20 GM103499 DRP (YS).

Schoeller INSTRUMENTS

Laboratory instruments – sales, services, validation
Life and medical sciences as well as industrial labs
Consumables sold by Schoeller Pharma Praha (https://pharma.cz)

https://instruments.cz

P_39

A novel cluster of putative regulatory sequences modulates Tbx1 gene expression

Sara Allegretti[1], Olga Lanzetta[2], Rosa Ferrentino[2], Ilaria Aurigemma[3], Antonio Baldini[1]

[1] *PhD program in Molecular Medicine and Medical Biotechnology, University Federico II, Naples, Italy*
[2] *Institute of Genetics and Biophysics, National Research Council, Naples, Italy*
[3] *Department of Chemistry e Biology, University of Salerno, Fisciano, Italy*

Background: Tbx1 function is involved in cardiac and pharyngeal development. Information regarding the genetic elements and molecular mechanisms that regulate the gene expression is still incomplete. We used in vitro differentiation, single cell biology, and bioinformatic tools to identify and validate regulatory elements of the gene.

Material and methods: We employed simultaneous single cells RNA-seq and ATAC-seq data from mouse ES cells (mESCs) differentiating into precardiac organoids; on these, we correlated chromatin accessibility and Tbx1 gene expression in distinct cell clusters and identified differentially accessible regions. We applied a machine-learning approach to score the probability of being enhancers using logistic regression. Finally, we manipulated putative enhancers by CRISPR-Cas9 to test their requirement for Tbx1 gene expression.

Results and Conclusion: We identified 14 putative regulatory sequences (PRS) on the Tbx1 locus, through integration of scRNAseq with scATACseq datasets. Using ATAC datasets in public repositories, we confirmed that ATAC peaks corresponding to the PRSs were also present in mouse embryo tissues. We focused on a cluster that includes 3 PRSs, named PRS10, 11, and 12, located approx. 10Kb upstream of the gene. PRS10 and PRS12 had a positive predictive score, while PRS11 had a lower score. With CRISPR-Cas9 technology, we generated mESC lines deleted for the entire cluster and we also deleted the 3 PRSs individually. We then differentiated the engineered clones into precardiac organoids to test the *Tbx1* gene expression profile. Loss of the entire cluster and of PRS10 and PRS12 individually, led to strong, significant reduction of *Tbx1* expression compared to the parental WT line. These results demonstrate that the enhancer cluster is required for *Tbx1* gene regulation. Gene expression analyses of clones lacking only PRS11 is in progress and will be presented at the meeting.

Funding: Telethon Foundation GMR22T1012, and Italy PRIN 2022XFE7M2.

P_40

Functional 3D in vitro myocardial model

Lucie Gágyorová, Štefan Zelenák, Katarzyna Anna Radaszkiewicz, Eliška Kohoutková, Jiří Pacherník

Institute of Experimental Biology, Faculty of Science, Masaryk University, Kamenice 5, 625 00 Brno, Czech Republic

Current cardiology focuses on the search for new sources of cardiomyocytes for transplantation therapy, and for the study of cardiomyopathies in patient-like models. New cardiomyocytes can be obtained from pluripotent stem cells or by primary reprogramming of e.g. fibroblasts. Both procedures can yield genotype-matched cardiomyocytes to those of the patient. At present, the preparation of a sufficient number of cardiomyocytes and the creation of a model simulating the myocardium for in vitro testing remain undefined.

In our laboratory, we focus on the development of cardiomyocytes from human pluripotent stem cells from patients. Using a variety of techniques, we create a 3D in vitro model of the myocardium to better reflect the complexity of the patient's myocardium compared to a 2D cell culture. The quality of the resulting myocardial models is assessed with respect to contraction stability, structure, phenotype and cellular organization. We work with cell spheroid and tissue ring models. Tissue rings in particular appear to be very promising models for future research because they can be used to observe the transmission of electrical activity and mechanical contraction within a single compartment.

To ensure stable contraction, we use the formation of conduction cardiomyocytes together with atrial cardiomyocytes and their subsequent connection with ventricular cardiomyocytes. The model thus becomes more complex, and we hypothesize that it better simulates the function of the real myocardium and its responses to experimental interventions to study pathological conditions and their treatment.

https://www.sci.muni.cz/ofiz/en/jiri-pachernik/

P_41

Scaffold moulding for a heterotypic mini-heart

Mariel Cano[1]*, Elena Cano[2]*, Cristina Pogontke[2], Adrián Ruiz-Villalba[2], José María Pérez-Pomares[2], Marcelo C Ribeiro[1,3], Robert Passier[1,4], Juan Antonio Guadix[2]

[1] *Applied Stem Cell Technologies, TechMed Centre, University of Twente, Enschede, The Netherlands*

[2] *Department of Animal Biology, Faculty of Sciences, University of Málaga; Malaga Institute of Biomedicine (IBIMA) - BIONAND Platform, Junta de Andalucía - University of Málaga, Málaga, Spain*

[3] *River BioMedics, Enschede, The Netherlands*

[4] *Department Anatomy and Embryology, Leiden University Medical Centre, Leiden, The Netherlands*

**These authors contributed equally*

Human cardiac organoids have been established by the scientific community as consistent human cardiac models with a predictivity level of 80%. Currently, cardiac tissue engineering is based on engineered morphogenesis of cardiomyocytes in hydrogels. While some attempts have been made to generate *in vitro* mini-hearts by guided morphogenesis, they are monotypic (only composed only of cardiomyocytes) and lack of cell-driven scaffold deformation which is crucial for physiological remodelling. We aim to generate a three-dimensional heterotypic mini-heart by combining, first, highly efficient hiPSCs differentiation protocols for the generation specific cardiac lineages, and second, a guided morphogenesis procedure based on a sacrificial moulding strategy for their assembly. Casting matrix-encapsulated cell mixtures into a degradable scaffold allows the generation of hollow three-dimensional structures that can be immersed and perfused with media. Interestingly, the encapsulating matrix allows cell remodelling to promote cell self-arrangement and the establishment of intercellular communication. Other advantages of our degradable scaffold moulding strategy are that it is an automated, scalable and customisable technology.

Our first generation mini-hearts are composed of mature cardiomyocytes and cardiac fibroblasts, assembled in a native-like balloon configuration. The matrix-encapsulated cells efficiently compact to form a multi-layered organoid capable of generating an encompassed contraction wave along the mini-heart. The implementation of a glass capillary outlet within the construct allows the measurement of the ejection volume and, therefore, the assessment of potential changes in the mini-heart contractility upon treatment. Producing better predictive cardiac models such as the mini-heart will aid drug discovery as well as cardiac disease modelling.

Funding: European Union. European Innovation Council (EIC-Pathfinder Challenges). BioRobot-MiniHeart; C. nº. 10107095.

Hyperlink: www.decalab.es

ESCIMEDA

Distributor of Eppendorf product for the Czech Republic and Slovakia.

https://www.escimeda.cz

P_42

Deciphering cardiac *versus* skeletal muscle fibrosis in Duchenne Muscular Dystrophy

Lidia Matias-Valiente[1,3], Francisco Hernandez-Torres[2,3], María López-Moreno[4], Cristina Chica-Esteban[4], Adrián Ruiz-Villalba[4], José María Pérez-Pomares[4], Marisol Montolio[5,6] and Amelia Eva Aranega[1,3]

[1] *Department of Experimental Biology, Faculty of Experimental Sciences, University of Jaen, Jaen, Spain*

[2] *Department of Biochemistry and Molecular Biology III and Immunology, Faculty of Medicine, University of Granada, Granada, Spain*

[3] *Medina Foundation, Technology Park of Health Sciences, 18016 Granada, Spain*

[4] *Department of Animal Biology, University of Málaga, Málaga, Spain; Biomedical Research Institute of Málaga (IBIMA-Plataforma BIONAND), Málaga, Spain*

[5] *Department of Cell Biology, Physiology and Immunology, Faculty of Biology, University of Barcelona, Spain.*

[6] *Duchenne Parent Project Spain Madrid, Spain*

Duchenne muscular dystrophy (DMD) is a devastating degenerative disease of skeletal muscles caused by loss of dystrophin, a key protein to maintain muscle integrity. The absence of this protein causes progressive muscle degeneration, aggravated by chronic inflammation, reduced regenerative capacity of muscle stem cells (MuSCs) and replacement of muscle with fibroadipose tissue, leading to cardiorespiratory complications which are the main cause of death. The first symptoms of DMD appear between the ages of 2 and 5 years characterized by muscle weakness and delayed motor development. However, it is recently well-known that by this time, cardiac pathology has already appeared. Due to the great impact heart disease has in these patients, it is imperative to understand the underlying mechanisms by which fibrosis is developed in each tissue. Our study sheds light on differences between the onset of fibrosis in cardiac and skeletal muscle tissues in the mdx mouse model for DMD. By histological analysis, we show that the fibrosis markers are strongly present in young mdx mice in the heart, while their tibialis anterioris muscles only display incipient signs of fibrotic areas. Moreover, comparative immunohistochemistry analyses reveal remarkable differences in how the fibrosis patterns are stablished in the cardiac and the skeletal dystrophic muscles during disease progression.

Overall, we show that cardiac fibrosis appears in the earliest stage of the DMD disease, even before muscle symptoms are detected. This is of particular importance, as a better understanding of the mechanisms that give rise to cardiac pathology in DMD would help to identify new molecular targets to treat the impact of cardiac consequences in this disease.

Funding: This work was partially supported by grants DUCHENNE_2018/001 (Duchenne Parent Project, Spain Foundation), PID2022-138163OB-C31 (Ministerio de Ciencia e Innovación, Gobierno de España) and ProyExcel_00513 (Consejería de Universidad, Investigación e Innovación, Junta de Andalucía).

Hyperlink: https://www.ujaen.es/grupo-de-investigacion-uja-miogenesis-cardiaca-y-esqueletica-regeneracion-muscular-cts-446

P_43

Atrial Structure, Function, and Development in Humans and Other Vertebrates

Neradilova C[1], Gregorovicova M[2], Kvasilova A[2], Kovanda J[1], Melenovsky V[3], Sedmera D[2]

[1] *Children's Heart Center, Second Faculty of Medicine, Charles University and Motol University Hospital, Prague, Czech Republic*

[2] *Institute of Anatomy, First Faculty of Medicine, Charles University, Prague, Czech Republic*

[3] *Department of Cardiology, Institute of Clinical and Experimental Medicine, Prague, Czech Republic*

Background: An increasing number of atrial catheterization and ablation procedures performed on patients raises calls for an improvement in our anatomical and physiological knowledge of the atria.

Methods: We summarized existing information on atrial anatomy, conduction properties, and function. In clinical practice, there are different ways to assess atrial function, such as echocardiography or catheterization. We have also focused on specific structures in the atria called the pectinate muscles (PM). To complement the data from non-model species, we sought to quantify the composition of the developing atria by performing a study of the relative surface area of the PM on histological sections of Siamese crocodile, Corn snake, Central bearded dragon, and Leopard gecko. We measured the total surface area of the PM in the right (RA) and left (LA) atrium during different stages of embryogenesis and expressed it as a percentage of the total atrial myocardial cross-sectional area.

Results and Conclusion: From the available data, it is obvious that the compact (free) wall in the atria of different species is consistently thin compared to the large differences in thickness of the ventricular wall. In our reptile study, we found that in the hearts of Leopard gecko and Central bearded dragon, the surface area of the PM occupied similarly around 20-45% of the total surface area of the RA and the LA. The PM in the heart of Corn snake occupied 40-56% of the total volume of both atria. The most variable results were found in Siamese crocodile hearts, where the PM area varied from 21 to 71% in the RA and from 28% to 61% in the LA. While in general the smaller hearts have a more extensive PM network than the larger ones, these results suggest that other factors may be in play, at least in the reptiles. The atria contain the sinoatrial and atrioventricular nodes and, if their function is not impaired, can also contribute a significant volume to the cardiac output. For all these reasons, they deserve attention from clinicians to avoid any unnecessary damage.

Funding: Supported by Czech Science Foundation 22-05271S and the Czech Health Research Council NU21-02-00402.

P_44

Model of Pressure Overload Imposed During the Proliferative Phase of Rat Heart Development: Mechanical and Electrophysiological Findings

Eva Zabrodska[1], Jaroslav Hrdlicka[2], Alena Kvasilova[1], Michaela Slegrova[1], Anna Jokelova[2], Veronika Olejnickova[1,2]

[1] *Institute of Anatomy, First Faculty of Medicine, Charles University, U Nemocnice 3, 128 00 Prague 2, Czech Republic*

[2] *Institute of Physiology, Czech Academy of Sciences, Videnska 1083, 142 00 Prague 4, Czech Republic*

Background: Cardiac hypertrophy as a response to increased pressure load is associated with impaired cardiac function and a proarrhythmogenic environment with reduced impulse conduction velocity (CV). This study investigates how these detrimental conditions are modified when pressure overload is imposed in the proliferative phase of cardiac development.

Methods: Pressure overload was induced by abdominal aortic constriction (AAC) in rats at postnatal day 2 (AAC-PD2). Control groups included sham-operated animals at PD2 and rats subjected to AAC surgery on PD6 (AAC-PD6). Final evaluations were performed at PD21 (n=5-6 per group). Functional outcomes of the left ventricle (LV) were measured by transthoracic echocardiography (TTE). Cardiomegaly was assessed through morphological and TTE analyses. The changes in myocardial electrophysiology were determined by ECG and optical mapping and accompanied by histological and immunohistochemistry examination.

Results and Conclusion: We observed a similar increase in the heart-to-body weight ratio in AAC-PD2 and AAC-PD6 rats (13.9 ± 6 in AAC-PD2, 12.2 ± 4 in AAC-PD6, and 4.3 ± 0.2 in sham-operated controls). However, while AAC-PD2 rats showed significantly higher relative LV thickness compared to sham controls, this increase was less pronounced in AAC-PD6 group. Both AAC-PD2 and AAC-PD6 animals revealed significantly decreased systolic function, as measured by fractional shortening, compared to the sham-operated animals. Electrophysiological analyses revealed preserved longitudinal and transversal CV (CV_L and CV_T) in the AAC-PD2 group (CV_L 82 ± 29 cm/s, CV_T 53 ± 10 cm/s) compared to the sham controls (CV_L 78 ± 14 cm/s, CV_T 58 ± 14 cm/s). In contrast, the AAC-PD6 group showed important CV reduction (CV_L 65 ± 13 cm/s, CV_T 44 ± 10 cm/s). These findings were corroborated by histological and immunohistochemical examinations. Our data demonstrate important electrophysiological adaptations to pressure overload imposed during the proliferative phase of cardiac development and provide detailed morphological and functional characterizations of this specific model.

This study was supported by the Czech Health Research Council: NU21J-02-00039.

P_45

Functional analysis of new genes potentially involved in ventricular maturation

Marcos Siguero-Álvarez[1,2], Violeta Sebastián-Serrano[1,2], Javier Santos-Cantador[1,2], Cristina Roy-Cordero[1,2], Declan P O'Regan[3], José Luis de la Pompa[1,2]

[1] *Intercellular signalling during development and disease, CNIC, Madrid, Spain*
[2] *CIBER de Enfermedades Cardiovasculares, Madrid, Spain*
[3] *MRC Laboratory of Medical Sciences, Imperial College London, London, UK*

Background: Disruption of myocardial maturation and defects in ventricular development, such as impairment of trabecular compaction leading to a thinned compact myocardium and a poorly formed ventricular conduction system are associated to cardiomyopathies and congenital heart disease. A recent GWAS study has identified candidate SNPs affecting genes involved in cytoskeletal arrangement during dendritic arborization, arterial specification or β-catenin signaling and proliferation, associated to increased trabecular complexity and lower risk of cardiovascular disease. New data coming from a bigger GWAS report point to Golgi Apparatus (GA) implication in trabeculation.

Methods: We have used RNA sequencing to study whether the expression of these genes was affected in our murine models of LVNC. We have generated conditional and standard knock-out mouse models carrying mutations in the identified genes using CRISPR-Cas9, to explore their functional and molecular effects at development, postnatal stages and adulthood, with a special focus on the ventricular conduction system. We have performed phenotypic analysis and have proved by qPCR, ISH and IHC that the deletions introduced lead to abrogation of gene expression.

Results and Conclusion: In our Nrg1 loss-of-function and gain-of-function models, both altering the patterning of the trabecular and compact myocardial wall, we found that, besides metabolic maturation markers, the expression of several of the genes affected by GWAS-identified SNPs was disrupted. We have generated 9 mouse lines, including three conditional lines corresponding to the presumed embryonic or early postnatal lethal knock-outs. Their initial phenotypic and molecular characterization will be reported. For instance, Mecom knock-out mice die before E10.5 and Pdzrn3 LOF mice appear feeble and smaller than WT littermates, but survive longer than previously reported.

We are also generating additional mutants in Cog5, a GA gene, that will be combined with our Gosr2 knock-out line. Preliminary characterization and the first results of the double heterozygotes will be presented.

Funding: Supported by Ministerio de Ciencia, Innovación y Universidades, Fundació La Caixa, FEDER, CIBER

SciMedia is dedicated to helping scientists worldwide improve the quality of their research. We specialize in high speed imaging systems for biological research applications. Our systems are designed to detect voltage, calcium, and intrinsic signals by providing the optimal combination of high speed, high resolution, and high signal to noise ratios.

SciMedia is the exclusive distributor for the MiCAM series High Speed Imaging Systems globally, with the exception of Japan. We have always been committed to providing excellent service worldwide, and we are constantly working to improve our hardware, software, and technical support to help our users.

https://www.scimedia.com/applications/cardiac/

P_46

Investigating the specification and maturation of the cardiac pacemaker

Tanishta Bhattacharya, Marco Tarasco, Thomas Jua[1], Didier Y. R. Stainier

Max Planck Institute for Heart and Lung Research, Bad Nauheim, Germany

Background: Cardiac arrhythmia, caused by abnormal heart pacing, affects up to 5% of the population. The cardiac conduction system plays a crucial role in generating electrical impulses and ensuring rhythmic contraction. Although several key players in the development of the cardiac pacemaker, the sinoatrial node (SAN), have been identified, little is known about the proteins and signaling pathways that lead to SAN maturation. This project aims to identify genes and signaling pathways affecting SAN formation and maturation while also modeling arrhythmias and aiding in the development of therapeutic interventions.

Methods: We used single-cell RNA sequencing (sc-RNA seq) data from zebrafish hearts across different time points and selected genes that were upregulated in the SAN region after heart looping (36 hours post fertilization). These SAN genes exhibit a high degree of conservation from zebrafish to humans. We then performed a crispant screen to identify candidates genes that, when mutated, lead to interesting phenotypic outcomes for further evaluation using stable mutant lines. Our phenotyping was based on functional assays such as cardiac contraction (heart rate, fractional shortening) and Ca+2 signaling analysis to understand how these processes are affected in the crispants.

Result and Conclusion: From a preliminary analysis of the sc-RNA seq data we selected 16 genes differentially upregulated in the SAN region at later stages of development. The crispant screen helped in obtaining quick insight into their potential role. Among these candidate genes is *lyve1a* (lymphatic vessel endothelial hyaluronic receptor 1a), a hyaluronan receptor that is specifically expressed in the SAN region. Another candidate, *atp2b1a* (plasma membrane Ca2+ transporting 1a) appears to control heart looping and heart rate as observed in crispant screen. We hypothesize that these genes, along with crucial signaling pathways, are playing an important role in driving SAN maturation and establishing a stable heartbeat and signal conduction.

Funding: Supported by Max Planck Institute for Heart and Lung Research; IMPRS,MOB and Boehringer Ingelheim Fonds

P_47

Brugada syndrome-associated Transcriptomic Remodeling Occurs Throughout *in vitro* Cardiac Development

Thomas Stervinou[1,†], Bastien Cimarosti[1,†], Robin Canac[1], Aurore Girardeau[1], Virginie Forest[1], Patricia lemarchand[1], Nathalie Gaborit[1,#] and Guillaume Lamirault[1,#]

[1]*Nantes Université, CHU Nantes, CNRS, INSERM, l'institut du thorax, F-44000 Nantes, France*

[† and #] *Contributed equally to this work*

Background: Brugada Syndrome (BrS) is a rare inherited cardiac rhythm disorder that can lead to ventricular fibrillation, syncope, and sudden cardiac death in young adults. Recent genetic studies suggest that abnormal cardiac development contributes to the pathogenesis of BrS. *In vitro* cardiac differentiation of human induced pluripotent stem cells (hiPSCs) mimics heart development at the cellular level up to a prenatal stage. This study aims at defining whether BrS impairs cardiac differentiation of hiPSCs.

Methods: We conducted (1) single-cell RNA-seq at the end of 2D cardiac differentiation (Day 30) on two control and two BrS hiPSC lines with different genetic backgrounds (one with a rare *RRAD* mutation, and one with no identified rare variant) ; (2) bulk RNA-seq analysis at each day of the cardiac differentiation of three control hiPSC lines and three BrS patient hiPSC lines (one with a rare *SCN5A* mutation and the two described above).

Results and Conclusion: (1) Single-cell analysis revealed that our cellular model is composed of three main cell types: cardiomyocyte-like, fibroblast-like, and epicardial-like cells. We observed a higher proportion of fibroblast-like and epicardial-like cells in BrS lines, while control lines have more cardiomyocyte-like cells. Notably, 94.45% of a fibroblast-like sub-cluster were Brugada cells. Pseudobulk transcriptomic analysis revealed differentially expressed genes involved in cardiac development, with altered expression of key cardiac transcription factors such as *HEY2*, *TBX3*, and *NR2F2* in cardiomyocyte-like cells. (2) Based on these findings, we performed bulk transcriptomic analysis in kinetics to analyze temporal alterations during cardiac differentiation. Differential gene expression analysis unveiled gene expression signatures specific to each BrS patients and expression alterations that were common to all 3 BrS-hiPSCs lines. Interestingly, the misexpressed genes were associated with the cell differentiation process.

By combining single-cell and bulk transcriptomic data during hiPSC cardiac differentiation, this study strongly suggests that BrS-associated transcriptomic remodeling occurs during cardiac development.

Funding: This work is funded by grants from The National Research Agency (WIRES ANR-22-CE17-0051-01) and from the Fédération Française de Cardiologie.

Poster Session 8 – Endocardium and vasculature

The **HELAGO** corporation was founded in March 1992 and is currently extending in the following fields:

- layouts, projects and manufacturing of laboratory furniture including fume hoods and laboratory tables
- layouts, projects and manufacturing of pharmacy interiors
- supply of laboratory apparatus, equipment and consumables
- specialized schoolrooms equipment for all kinds of schools and educational institutions
- teaching materials for the natural science classes
- teaching materials for engineering schools and universities
- medical simulators and phantoms for the training of first aid, nursing and medical skills

https://www.helago-cz.com

P_48

ANATOMICAL-CLINICAL STUDY OF THE HUMAN CAROTID SYSTEM IN CARDIOMETABOLIC AND RESPIRATORY DISEASES

Inés Smith-Fernández, M. Carmen Fernández, Víctor Smith, Rita Carmona

Department of Human Anatomy and Embryology, Legal Medicine and History of Science, Faculty of Medicine, University of Málaga. Campus de Teatinos, s/n 20071. Málaga. Spain

The carotid artery system is a crucial vascular network responsible for the irrigation the head, neck and central nervous system. It also plays a role in baroreception and chemoreception through the carotid sinus and carotid body. Various cardiometabolic and respiratory diseases have been associated with morphological variations in the carotid artery network. However, unlike others published using imaging techniques for diagnosis in living individuals, these studies have been performed in the carotid system in 30 donated corpses.

This study presents a descriptive report, followed by anatomical-histological and statistical analysis to identify anatomical-clinical correlations with cardiometabolic and respiratory diseases, in addition to social habits, such as smoking.

Our evidence has identified significant differences between the size and components of the carotid system's arterial wall and diverse pathologies such as arterial hypertension, Type 2 Diabetes mellitus, heart disease, chronic obstructive pulmonary disease and asthma. In particular, diabetic donors show a significantly larger carotid body and smokers show decreased carotid arterial intima-media thickness and percentage of arterial smooth muscle cells. In chronic obstructive pulmonary disease, the common, external and internal carotid arteries show a significantly shorter total and internal diameter than control donors.

Cardiometabolic and respiratory diseases provoke anatomical alterations in the carotid artery system. A better knowledge of these alterations could be important in determining risk factors for some very highly prevalent pathologies, especially in elderly populations.

Keywords: carotid system, carotid body, carotid sinus, respiratory disorders, hypertension, Type 2 Diabetes mellitus, atherosclerosis.

P_49

Akt3 Controls Lamin A/C Expression in Coronary Vasculature; Ablation of Akt3 Results in Dilated Cardiomyopathy

Fatemeh Nasehi[1], Amy Bradshaw[2], Ann C. Foley[1,] and Robin C. Muise-Helmericks[3]

[1] *Department of Bioengineering, Clemson University, USA*
[2] *Division of Cardiology, Medical University of South Carolina, USA*
[3] *Department of Regenerative Medicine and Cell Biology, Medical University of South Carolina, USA*

Background: The hearts of Akt3 mutant mice develop dilated cardiomyopathy, but the mechanism is not fully understood. Members of the Akt/protein kinase B family of serine-threonine kinases have been implicated in many cellular processes, including growth, proliferation, and cell survival. Akt3 is unique among the Akts in its regulation of lamin A/C, a nuclear envelope protein central to the cellular response to substrate stiffness. Lamin A/C mutations are associated with increased collagen I and reductions in junctional integrity.

Methods: Both in vitro analyses and in vivo analyses were performed. In vitro, cultured vascular endothelial cells were used for transduction with shRNAs and overexpression analyses. General biochemical methods were performed. For in vivo analyses, E 14.5 embryos and adult mice were used. In adult animals, ECHOs, MICROFIL casts and morphogenic analyses were performed. Tensile strength analyses were performed on papillary muscles. For all analyses at least n=3 mice per genotype were analyzed.

Results: In vascular endothelial cells Akt3 depletion results in increased expression of lamin A/C resulting in nuclear envelope dysmorphia. On soft substrates, Akt3 expression is higher and localized to the nucleus. This is inversely proportional to lamin A/C expression, which is high in stiff substrates.

In vivo, Akt3 null hearts are smaller than age-matched wild-types during embryonic stages (E14.5), have decreased cell proliferation (Aurora B kinase and Ki67), and increased cell death (Cleaved caspase 3). By ten weeks after birth, Akt3 mutant hearts have severely thinner ventricular walls but increased overall size, resulting in dilated cardiomyopathy (DCM). Akt3 nulls at E14.5 have hemorrhaging and reductions in branching morphogenesis. In adults, Akt3 null hearts have reduced capillary density, a disrupted patterning of blood vessels and, leaky vasculature. MICRIFIL casts of Akt3 null hearts reveal a lack of capillary beds and tortured vasculature. Tortured vasculature occurs due to the misregulation of ECM deposition. Accordingly, adult hearts of Akt3 null animals have increased lamin A/C and collagen I expression and increased muscle stiffness. ECHO analysis shows a DCM phenotype in males that is not replicated in female mice.

Conclusion: Our findings suggest that Akt3 mutant mice might be an excellent model to study the etiology of lamin A/C-based DCM. Alterations in lamin A/C expression or mutation result in nuclear envelope deformations leading to diseases such as DCM. Lamin A/C protein expression is closely correlated with collagen deposition and biomechanical stiffness. Interstitial and perivascular fibrosis increases during the progression of DCM, yet the role of fibroblasts and endothelial cells in DCM progression is poorly understood. We hypothesize that disruption of lamin A/C expression by Akt3 ablation in endothelial cells and fibroblasts results in an aberrant deposition of extracellular matrix affecting DCM progression.

P_50

ACTA2-related Hereditary Thoracic Aortic Aneurysms and Dissections (HTAAD) and Cellular Explorations (_ACTA2_ - FACE Project): Translational research of patients towards the α-actine structure to discover the pathophysiological mechanisms of the disease

Amel SEDDIK [1], Laurence BAL-THEOLEYRE [1,2], Natacha BROUCQSAULT [1], Stéphane ZAFFRAN[1]

[1] *Marseille Medical Genetics, Inserm U1251, Aix-Marseille University, Marseille, France*

[2] *Constitutive Center for Marfan Syndrome and Related Disorders, Aorta Timone Center, Timone University Hospital Center for Adults, Marseille, France*

Background*:* *ACTA2*-related aortic diseases represent 10 to 20% of patients without syndromic HTAAD. *ACTA2* gene encodes α-actin protein, specific protein of vascular smooth muscle cell (VSMC) involved in mechano-transduction through the interaction of actin-myosin filaments with the extracellular matrix (ECM), and the regulation of transcription of others contractile genes. Clinical practice is difficult due to incomplete penetration of *ACTA2* pathogenic variants, unusual cardiovascular expression dealing with cerebrovascular or coronary stenosis, and a higher risk of aortic dissection (AD) without prior aortic aneurysm. Furthermore, recent phenotype-genotype correlations, such as smooth muscle cell deficiency syndrome (SMDS) in the presence of R179 variants, suggest that the localization of the mutated *ACTA2* variant plays a role in VSMCs dysfunction.

Method: To provide novel insight into the genotype-phenotype correlations, we created and validated a new VSMC model from *ACTA2*$^{+/-}$ patients produced by differentiation of human induced pluripotent stem cells (hiPSCs) derived from peripheral blood mononuclear cells. We performed functional analysis of hiPSC-derived VSCMs to examine the cytoskeleton organization, the proliferation, the migration, the contraction, and the transcriptome. Four patients with different variants were compared to three controls: R179H variant associated to SMDS; G148R variant associated to SMDS-like phenotype with stroke and recurrent AD in adolescence; P335R and R212Q variants associated to familial AD before third decade.

Results: *ACTA2*$^{G148R/+}$ VSMCs have a smaller size with less actin filaments, they are hyperproliferative and low migratory capacity. Transcriptomic analysis revealed a down regulation of genes implicated in cell adhesion, differentiation, and in actin polymerization. *ACTA2*$^{R179H/+}$ VSMCs have abnormal nuclear shape surrounded by a large perinuclear ring of globular actin associated with a loss of contractile phenotype through an up-regulation of genes implicated in ECM structure and organization.

Conclusion: Our hiPSC-derived VSMC model successfully distinguished phenotypic differences among *ACTA2* variants *in vitro*, allowing to further identification of key regulators involved in VSMC de-differentiation.

P_51

Pdia3 Regulates PDGF Crosstalk Required for the Diametric Positioning of Two Cardiac Neural Crest Cell Streams to Ensure an Even Division of the Cardiac Outflow Tract

Ye Wang[1,*], Yabo Fang [1,*], Min Zhang[1], Jonathan Klownoski[2], Junjie Yang[1], Cecilia W. Lo[2,#], Zhen Zhang[1,#]

[1] *Pediatric Translational Medicine Institute and Pediatric Congenital Heart Disease Institute, Shanghai Children's Medical Center, Shanghai Jiao Tong University School of Medicine, China*
[2] *Department of Developmental Biology, University of Pittsburgh, U.S.A.*

[*] *Equal contribution,* [#] *Co-corresponding*

Septation of the cardiac outflow tract (OFT) from a single channel into two tubes of similar size represents a distinctive morphogenetic event during embryonic development. Neural crest cells (NCCs), originating from the dorsal neural tube, form two opposing streams in the OFT to ensure an even division. Many conotruncal defects originate from the disproportional division of the OFT, but the regulatory mechanisms governing the positioning of NCC streams within the OFT remain unclear. Mechanistic insights have emerged from analysis of a mouse line recovered from a mutagenesis screen exhibiting conotruncal defects including persistent truncus arteriosus (PTA). Although the entry of NCCs into the OFT was normal in these mutants, the two NCC streams remained in close proximity instead of diverging to assume opposite positions along the OFT, thereby leading to abnormal OFT division. Genetic analysis identified a L16P missense mutation in *Pdia3*, encoding a disulfide isomerase located in the endoplasmic reticulum (ER) that facilitates protein folding. However, we did not detect enhanced ER stress in mutant OFT. Using a substrate trapping strategy, we identified Pdgfra, which is essential for OFT development, as a substrate of Pdia3. Co-immunoprecipitation confirmed Pdia3 interaction with Pdgfra in the OFT. The expression of Pdgfra and its downstream target was significantly reduced in cardiac NCCs of *Pdia3* mutants. NCC-specific deletion of *Pdia3* recapitulated the NCC malpositioning defect observed in the *Pdia3* missense mutant. Loss of Pdgfra signaling led to premature differentiation of NCCs into smooth muscle cells and failed migration in response to polarized Pdfga expression in the OFT wall. Clinical relevance is indicated by the conformity of NCC-related defects in congenital heart disease patients with *PDIA3* variants. These findings indicate that *Pdia3* regulates PDGF crosstalk to guide the diametric positioning of two NCC streams in the OFT, which is a prerequisite for an even division of the OFT.

Interrogating the tumor-immune landscape with a novel automated RNAscope™ assay for multiplexed detection of RNA and protein

Anushka Dikshit[1], Sonali Deshpande[1], Sara Wrobel[2], Ge-Ah Kim[1], Sayantani Basak[1], Li-Chong Wang[1], Ching-Wei Chang[1] and Maithreyan Srinivasan[1]
Advanced Cell Diagnostics, a Bio-Techne brand, Newark, CA, USA, 94560 [1], Bio-Techne Ltd, 19 Barton Ln, Abingdon, OX14 3NB, UK [2].

Introduction

Understanding tissue heterogeneity is critical for elucidating cell-cell interactions with important implications in immuno-oncology, inflammation, and neuroscience. Tissue heterogeneity poses immense challenges to understanding underlying molecular mechanisms using techniques such as qRT-PCR or bulk sequencing. While single-cell RNA sequencing can provide information about precise cellular composition of tissues, data analysis can be cumbersome and spatial context is lost. With single-cell spatial platforms such as RNAscope™, target gene and protein expression can be visualized to characterize cell types and tissue neighborhoods. Here, we demonstrate a novel method for the simultaneous detection of RNA and protein using a modified co-detection assay.

This novel co-detection assay enables visualization of a combination of up to 12 RNA and/or protein targets on the same sample. We used a set of antibodies targeting key immune and tumor cell markers- PD-L1, CD3, CD4, CD8, CD68, FOXP3 and Pan-CK, along with RNA biomarkers to interrogate the tumor microenvironment (TME) in human FFPE tumor samples. Using a combination of RNA and protein targets, we characterized different subtypes of T cells and tumor cells in the TME.

This assay based on the RNAscope™ HiPlex technology offers a powerful technique for visualizing target RNA biomarkers in specific cells identified by cell-marker protein expression. This can enable multiomic analysis to identify novel biomarkers and therapeutic targets.

Method

RNAscope RNA-protein semi-automated co-detection workflow

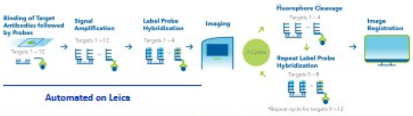

Figure 1: RNA-protein same slide co-detection workflow for up to 12 targets. This workflow is automated on the Leica BOND RX for the initial steps which include addition of all target antibodies and RNA probes, building the amplification trees and first round of label probe hybridization.

A) Human tonsil

RNA	Protein
Hs-POLR2A	CD3a
Hs-GAPDH	CD4
Hs- LDHA	CD8
Hs-RPLPO-X-RPLPOP2	FOXP3
-	PD-1
-	CD68
-	PD-L1
-	PanCK

B) Human cervical cancer

RNA	Protein
Hs-POLR2A	CD3
Hs-PPIB	CD8
Hs-UBC	PanCK-S100A4-S100B
Hs-HRPT1	PD-L1

Results

Immune cell subtypes identified using a targeted multiplex antibody panel with the new RNA-Protein co-detection workflow

FFPE Human Tonsil

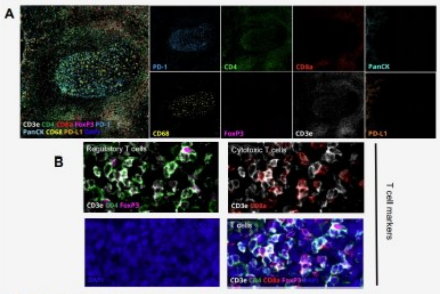

Figure 2: Expression of immune and tumor cell markers detected using an RNAscope compatible antibody panel. A, expression of 8 cell marker proteins in the tonsil tissue, B, T cell subsets such as regulatory T cells and cytotoxic T cells were visualized at a higher magnification using marker-specific antibodies.

Multiplex positive control RNA expression with the new RNA-Protein co-detection workflow

FFPE Human Tonsil

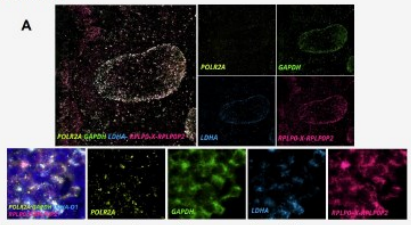

Figure 3: Expression of control RNA marker genes indicate comparable sensitivity and specificity to the standard RNAscope HiPlex assay. A, expression of 4 control RNA genes with varying levels of expression in tonsil tissue. B, punctate expression of control RNA genes visualized with higher magnification.

Multiomic analysis by detection of immune cell markers with positive control RNA targets in FFPE cervical cancer tissue

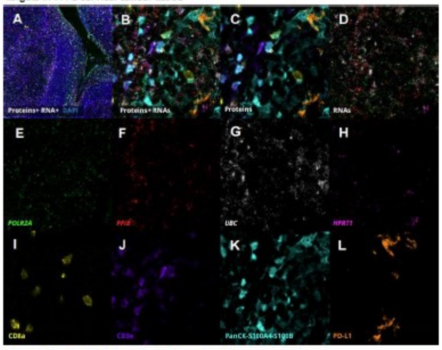

Figure 4: Overlay of 4 RNA and 4 protein targets on the same cervical cancer section. A, low magnification overview of 4 RNAs and 4 proteins using the co-detection workflow, B-D, demonstrating combination of RNA targets, protein targets and an overlay of RNA and protein targets in a region within the cervical cancer tissue, E-H, RNA markers in individual channels, I-L , protein markers in individual channels.

Summary

- The new RNA-protein co-detection workflow allows increased multiplexing capability with detection of up to 12 RNA + protein markers in combination on the same section.
- By combining immune cell marker antibodies with RNA probes for cytokines , this technique can provide a comprehensive landscape of tumor-immune interactions.
- This assay enables development of custom panels based on target proteins and RNAs of interest.
- The partially automated workflow on the Leica BOND RX ensures faster turn around time compared to the fully manual HiPlex workflow.

Conclusion

Built on the flagship RNAscope technology the new RNA-protein co-detection workflow enhances the current capability of the RNAscope co-detection assay by allowing a combination of up to 12 target RNAs and proteins to be detected simultaneously.

bio-techne // Global Developer, Manufacturer, and Supplier of High-Quality Reagents, Analytical Instruments, and Precision Diagnostics.
INCLUDES R&D Systems™ Novus Biologicals™ Tocris Bioscience™ ProteinSimple™ ACD™ ExosomeDx™ Asuragen™ Lunaphore™

INDEX TO ABSTRACTS

Alfano, Daniela P_04, P_18
Allegretti, Sara P_39
Alqahtani, Ahlam O_15
Aranega, Amelia P_21, P_22, P_42
Bakkers, Jeroen P_25
Baldini, Antonio O_17, O_21, P_04, P_07, P_18, P_39
Bamforth, Simon O_33
Bell, Laura O_04, O_28
Bhattacharya, Tanishta P_46
Bibonne, Anne
Bodmer, Rolf O_07, O_32
Bønnelykke, Tobias O_20
Byerly, Kathryn P_19
Campione, Marina
Caño Carrillo, Sheila O_11
Carmona, Rita P_22, P_48
Chaudhry, Bill O_15
Cortes, Claudio O_08, O_20
Crucean, Adrian
D'Amato, Gaetano P_23
de la Pompa , José Luis O_16, O_34, P_45
Deepe, Raymond O_14, P_20, P_24
del Monte Nieto, Gonzalo
Derrick, Chris
Desgrange, Audrey O_18
Devji, Inara O_14, P_20, P_24
Dias, Tiago P_01, P_26
Dondi, Cristiana O_32
Drummond, Jenna O_14, P_20, P_24
Dubois, Nicole
Duong Phu, Duy Max P_02
Durbin, Matthew P_03
Elena, Cano Rincón O_22, O_23
Field, Loren
Firulli, Anthony
Firulli, Beth
Flores Garza, Brenda Giselle O_16
Franco, Diego O_11, P_21, P_22
Fullerton, Millie O_33
Gaborit, Nathalie P_47
Gágyorová, Lucie P_40
Gehlot, Rupal O_13
Godin, Sarah
Gregorovicova, Martina P_30, P_43
Grossfeld, Paul
Guadix Dominguez, Juan Antonio P_41
Gupta, Ritvik P_35
Harvey, Andrew O_14, P_20, P_24
Harvey, Richard KN_1
Henderson, Deborah O_15
Hitz, Marc-Phillip P_08, P_14
Houyel, Lucile
Iacolare, Olimpia P_04, P_18
Ibrahim, Stephanie O_93
Illingworth, Elizabeth O_17, O_21
Ji, Caoyu P_05
Kaminsky, Sabrina O_24
Kelly, Robert O_20, P_23
Kolesova, Hana P_06, P_09
Koubsky, Karel O_02
Kulkarni, Radha Ajay O_27
Kvasilova, Alena P_43, P_44
Lania, Gabriella P_07
Lanzetta, Olga O_21, P_39
Larsen, Lars Allan
Li, Rich (Gang) O_09
Loizzi, Brian
Lozano Velasco, Estefania O_11, P_21, P_22
Lundegaard, Pia Rengtved O_30
Mancilla, Jourdano P_32
Martucciello, Stefania O_17
Masmaliyeva, Rafiga P_08
McCoy, Jamie
Meilhac, Sigolene O_06, O_18, P_12
Michel, Louise P_23
Moazzen, Hoda
Muise-Helmericks, Robin P_49
Narumanchi, Suneeta P_33
Neffeova, Kristyna P_06, P_09
Neradilova, Caroline P_43
Nicol, Thomas
Noel, Emily P_11, P_17
Nunes Vieira, Joaquim O_10, P_01, P_26
Ochandorena-Saa, Amaia P_12
Olejníčková, Veronika P_06, P_09, P_44
Osterwalder, Marco O_25
Palmquist, Paul O_06
Panakova, Daniela
Patra, Chinmoy P_13
Pau Navalón, Alba O_34
Paul, Priyanka
Peces-Barba-Castano, Laura P_31
Pérez Pomares, José María O_06, O_22, P_41, P_42
Philipp, Melanie P_02, P_16
Phillips, Helen O_33
Phookan, Ranan P_19
Postma, Alex P_14
Rai, Aparna
Roland, Virginia O_25
Ruiz Villalba, Adrian O_22, P_41, P_42
Sailer, Steffen Alexander P_16
Saleem Uddin, Sadaf
Sankova, Barbora P_30
Schwach, Verena O_12
Seddik, Amel P_50
Sedmera, David P_09, P_30, P_43
Sendra, Miquel O_05
Serpooshan, Vahid
Shou, Weinian P_03
Siguero-Álvarez, Marcos O_34, P_45

Silva, Nuno P_37
Smith, Kelly O_31
Smits, Anke
Snashall, Corinna P_17
Sparrow, Duncan O_03, O_04, O_28, P_01, P_26
Stainier, Didier O_13, O_24, O_27, O_30, P_31, P_35, P_46
Stathopoulou, Nancy O_26
Stefanovic, Sonia O_03
Steimle, Jeffrey O_29
Stervinou, Thomas P_47
Sugi, Yukiko P_38
Tarasco, Marco O_30, P_46
Tarolli, Hannah O_14, P_20, P_24
Tessadori, Federico P_25
Tomek, Viktor O_01
Torres Sanchez, Miguel O_05, P_34
Tyser, Richard
Valenzuela, Mariana P_26
van den Hoff, Maurice KN_2
Villa Del Campo, Cristina P_34
Wessels, Andy O_14, P_20, P_24
Wu, Bingruo
Wu, Mingfu O_19
Yang, Zhongzhou P_05, P_27, P_28
Zábrodská, Eva P_06, P_09, P_44
Zhang, Zhen P_51
Zhao, Tianyang P_28
Zhou, Bin

LIST OF PARTICIPANTS

Daniela Alfano
Naples
Italy
daniela.alfano@igb.cnr.it

Sara Allegretti
Napoli
Italy
sara.allegretti@unina.it

Ahlam Alqahtani
Newcastle upon Tyne
United Kingdom
Ahlam.Alqahtani@ncl.ac.uk

Amelia Aranega
Jaen
Spain
aaranega@ujaen.es

Jeroen Bakkers
Utrecht
Netherlands
j.bakkers@hubrecht.eu

Antonio Baldini
Napoli
Italy
bldntn@gmail.com

Simon Bamforth
Newcastle upon Tyne
United Kingdom
simon.bamforth@newcastle.ac.uk

Laura Bell
Oxford
United Kingdom
laura.bell@dpag.ox.ac.uk

Tanishta Bhattacharya
Bad Neuheim
Germany
Tanishta.Bhattacharya@mpi-bn.mpg.de

Anne Bibonne
Nantes
France
anne.bibonne@univ-nantes.fr

Rolf Bodmer
La Jolla
United States
rolf@sbpdiscovery.org

Tobias Bønnelykke
Marseille
France
tobias.bonnelykke@univ-amu.fr

Kathryn Byerly
Charleston, SC
United States
byerlyk@musc.edu

Marina Campione
Padova
Italy
campione@bio.unipd.it

Sheila Caño Carrillo
Jaen
Spain
scano@ujaen.es

Rita Carmona
Málaga
Spain
rita@uma.es

Bill Chaudhry
Newcastle upon Tyne
United Kingdom
bill.chaudhry@newcastle.ac.uk

Claudio Cortes
Oxford
United Kingdom
claudio.cortesrodriguez@idrm.ox.ac.uk

Adrian Crucean
Birmingham
United Kingdom
adrian.crucean@nhs.net

Gaetano D'Amato
Marseille
France
gaetano.damato@univ-amu.fr

José Luis de la Pompa
Madrid
Spain
jlpompa@cnic.es

Raymond Deepe
Charleston, SC
United States
deepe@musc.edu

Gonzalo del Monte Nieto
Clayton
Australia
gonzalo.delmontenieto@monash.edu

Chris Derrick
Newcastle upon Tyne
United Kingdom
chris.derrick@ncl.ac.uk

Audrey Desgrange
Paris
France
audrey.desgrange@institutimagine.org

Inara Devji
Charleston, SC
United States
devji@musc.edu

Tiago Dias
London
United Kingdom
tiago.dias@kcl.ac.uk

Cristiana Dondi
San Diego
United States
cdondi@sbpdiscovery.org

Jenna Drummond
Charleston, SC
United States
drummonj@musc.edu

Nicole Dubois
New York, NY
United States
nicole.dubois@mssm.edu

Duy Max Duong Phu
Tübingen
Germany
duy-max.duong-phu@uni-tuebingen.de

Matthew Durbin
Indianapolis, IN
United States
mddurbin@iu.edu

Cano Rincón Elena
Málaga
Spain
ecano@uma.es

Loren Field
Indianapolis, IN
United States
ljfield@iu.edu

Anthony Firulli
Indianapolis, IN
United States
tfirulli@iu.edu

Beth Firulli
Indianapolis, IN
United States
bfirulli@iu.edu

Brenda Giselle Flores Garza
Madrid
Spain
bgfloresg@cnic.es

Diego Franco
Jaen
Spain
dfranco@ujaen.es

Millie Fullerton
Newcastle upon Tyne
United Kingdom
m.fullerton2@newcastle.ac.uk

Nathalie Gaborit
Nantes
France
nathalie.gaborit@univ-nantes.fr

Lucie Gágyorová
Brno
Czech Republic
lucie.gagyorova@seznam.cz

Rupal Gehlot
Bad Neuheim
Germany
rupal.gehlot@mpi-bn.mpg.de

Sarah Godin
Nantes
France
sarah.godin@etu.univ-nantes.fr

Martina Gregorovicova
Prague
Czech Republic
martina.gregorovicova@lf1.cuni.cz

Paul Grossfeld
San Diego
United States
pgrossfeld@health.ucsd.edu

Juan Antonio Guadix Dominguez
Málaga
Spain
jaguadix@uma.es

Ritvik Gupta
Bad Neuheim
Germany
Ritvik.Gupta@mpi-bn.mpg.de

Andrew Harvey
Charleston, SC
United States
harveyan@musc.edu

Richard Harvey
Darlinghurst
Australia
r.harvey@victorchang.edu.au

Deborah Henderson
Newcastle upon Tyne
United Kingdom
deborah.henderson@newcastle.ac.uk

Marc-Phillip Hitz
Oldenburg
Deutschland
Hitz.Marc-Phillip@klinikum-oldenburg.de

Lucile Houyel
Paris
France
latalante@wanadoo.fr

Olimpia Iacolare
Napoli
Italy
olimpia.iacolare@igb.cnr.it

Stephanie Ibrahim
Marseille
France
stephanie.ibrahim@univ-amu.fr

Elizabeth Illingworth
Fisciano
Italy
eillingworth@unisa.it

Caoyu Ji
Nanjing
China (Mainland)
northwest_cpu@163.com

Sabrina Kaminsky
Mannheim
Germany
sabrina.kaminsky@medma.uni-heidelberg.de

Robert Kelly
Marseille
France
Robert.Kelly@univ-amu.fr

Hana Kolesova
Prague
Czech Republic
hkole@lf1.cuni.cz

Karel Koubsky
Prague
Czech Republic
karel.koubsky@fnmotol.cz

Radha Ajay Kulkarni
Bad Neuheim
Germany
Radha.Kulkarni@mpi-bn.mpg.de

Alena Kvasilova
Prague
Czech Republic
alena.kvasilova@lf1.cuni.cz

Gabriella Lania
Naples
Italy
gabriella.lania@igb.cnr.it

Olga Lanzetta
Ischia
Italy
o.lanzetta91@gmail.com

Lars Allan Larsen
Copenhagen
Denmark
larsal@sund.ku.dk

Rich (Gang) Li
Houston, TX
United States
rli@texasheart.org

Brian Loizzi
Charleston, SC
United States
loizzi@musc.edu

Estefania Lozano Velasco
Jaen
Spain
evelasco@ujaen.es

Pia Rengtved Lundegaard
Copenhagen
Denmark
plundegaard@sund.ku.dk

Jourdano Mancilla
Montpellier
France
jourdano.mancilla@igf.cnrs.fr

Stefania Martucciello
Fisciano
Italy
smartucciello@unisa.it

Rafiga Masmaliyeva
Oldenburg
Germany
rafiga.masmaliyeva@uni-oldenburg.de

Jamie McCoy
Sheffield
United Kingdom
j.mccoy@sheffield.ac.uk

Sigolene Meilhac
Paris
France
meilhac@pasteur.fr

Louise Michel
Marseille
France
louise.michel@univ-amu.fr

Hoda Moazzen
Aachen
Germany
hmoazzen@ukaachen.de

Robin Muise-Helmericks
Charleston, SC
United States
musehelm@musc.edu

Suneeta Narumanchi
Helsinki
Finland
suneeta.narumanchi@helsinki.fi

Kristyna Neffeova
Prague
Czech Republic
kristyna.neffeova@lf1.cuni.cz

Caroline Neradilova
Prague
Czech Republic
caroline.neradilova@gmail.com

Thomas Nicol
Oxford
United Kingdom
thomas.nicol@dpag.ox.ac.uk

Emily Noel
Sheffield
United Kingdom
e.s.noel@sheffield.ac.uk

Joaquim Nunes Vieira
London
United Kingdom
joaquim.nunes_vieira@kcl.ac.uk

Amaia Ochandorena-Saa
Paris
France
amaia.ochandorena-saa@pasteur.fr

Veronika Olejníčková
Prague
Czech Republic
Veronika.Olejnickova@lf1.cuni.cz

Marco Osterwalder
Bern
Switzerland
marco.osterwalder@unibe.ch

Paul Palmquist
Paris
France
paul.palmquist@institutimagine.org

Daniela Panakova
Kiel
Germany
daniela.panakova@uksh.de

Chinmoy Patra
Pune
India
cpatra@aripune.org

Alba Pau Navalón
Madrid
Spain
alba.pau@cnic.es

Priyanka Paul
Kiel
Germany
Priyanka.Paul@uksh.de

Laura Peces-Barba-Castano
Bad Neuheim
Germany
laura.peces-barba-castano@mpi-bn.mpg.de

José María Pérez Pomares
Málaga
Spain
jmperezp@uma.es

Melanie Philipp
Tübingen
Germany
melanie.philipp@uni-tuebingen.de

Helen Phillips
Newcastle upon Tyne
United Kingdom
Helen.Phillips@ncl.ac.uk

Ranan Phookan
Charleston, SC
United States
phookan@musc.edu

Alex Postma
Amsterdam
Netherlands
a.v.postma@amsterdamumc.nl

Aparna Rai
Madrid
Spain
aparna.rai@cnic.es

Virginia Roland
Bern
Switzerland
virginia.rolandvictor@unibe.ch

Adrian Ruiz Villalba
Málaga
Spain
adruiz@uma.es

Steffen Alexander Sailer
Tübingen
Germany
steffen-alexander.sailer@uni-tuebingen.de

Sadaf Saleem Uddin
Kiel
Germany
sadaf.saleemuddin@uksh.de

Barbora Sankova
Prague
Czech Republic
barbora.sankova@lf1.cuni.cz

Verena Schwach
Twente
Netherlands
v.schwach@utwente.nl

Amel Seddik
Marseille
France
amel.seddik@univ-amu.fr

David Sedmera
Prague
Czech Republic
david.sedmera@lf1.cuni.cz

Miquel Sendra
Madrid
Spain
miquel.sendra@hotmail.com

Vahid Serpooshan
Atlanta, GA
United States
vahid.serpooshan@emory.edu

Weinian Shou
Indianapolis, IN
United States
wshou@iu.edu

Marcos Siguero-Álvarez
Madrid
Spain
msiguero@cnic.es

Nuno Silva
Toulouse
France
nuno-valerio.santos-silva@univ-tlse3.fr

Kelly Smith
Melbourne
Australia
kelly.smith1@unimelb.edu.au

Anke Smits
Leiden
Netherlands
a.m.smits@lumc.nl

Corinna Snashall
Sheffield
United Kingdom
cmsnashall1@sheffield.ac.uk

Duncan Sparrow
Oxford
United Kingdom
duncan.sparrow@dpag.ox.ac.uk

Didier Stainier
Bad Neuheim
Germany
didier.stainier@mpi-bn.mpg.de

Nancy Stathopoulou
Oxford
United Kingdom
nancy.stathopoulou@paediatrics.ox.ac.uk

Sonia Stefanovic
Marseille
France
sonia.stefanovic@inserm.fr

Jeffrey Steimle
Houston, TX
United States
jeffrey.steimle@bcm.edu

Thomas Stervinou
Nantes
France
thomas.stervinou@univ-nantes.fr

Yukiko Sugi
Mie
Japan
sugi-y@med.mie-u.ac.jp

Marco Tarasco
Bad Neuheim
Germany
Marco.Tarasco@mpi-bn.mpg.de

Hannah Tarolli
Charleston, SC
United States
tarolli@musc.edu

Federico Tessadori
Utrecht
Netherlands
f.tessadori@hubrecht.eu

Viktor Tomek
Prague
Czech Republic
viktor.tomek@fnmotol.cz

Miguel Torres Sanchez
Madrid
Spain
mtorres@cnic.es

Richard Tyser
Cambridge
United Kingdom
rt593@cam.ac.uk

Mariana Valenzuela
London
United Kingdom
mariana.valenzuela_sanchez@kcl.ac.uk

Maurice van den Hoff
Amsterdam
Netherlands
m.j.vandenhoff@amsterdamumc.nl

Cristina Villa Del Campo
Madrid
Spain
CVILLA@CNIC.ES

Andy Wessels
Charleston, SC
United States
wesselsa@musc.edu

Bingruo Wu
Chicago, IL
United States
bingruow@uchicago.edu

Mingfu Wu
Houston, TX
United States
mwu25@central.uh.edu

Zhongzhou Yang
Nanjing
China (Mainland)
zhongzhouyang@nju.edu.cn

Eva Zábrodská
Prague
Czech Republic
eva.zabrodska@lf1.cuni.cz

Zhen Zhang
Shangai
China (Mainland)
zhenzhang@sjtu.edu.cn

Tianyang Zhao
Nanjing
China (Mainland)
zhaoty@smail.nju.edu.cn

Bin Zhou
Chicago, IL
United States
bin.zhou@bsd.uchicago.edu

NOTES

NOTES

A BIG THANKS TO ALL OUR EXHIBITORS AND PARTNERS

The ultrasound machine for the hands-on course and Wednesday afternoon demonstration is kindly provided by GE Healthcare.

ORGANISING SUPPORT

ENDORSEMENT

CATERING

SAVE THE DATE!

International Cardiovascular Development, Anatomy and Regeneration meeting

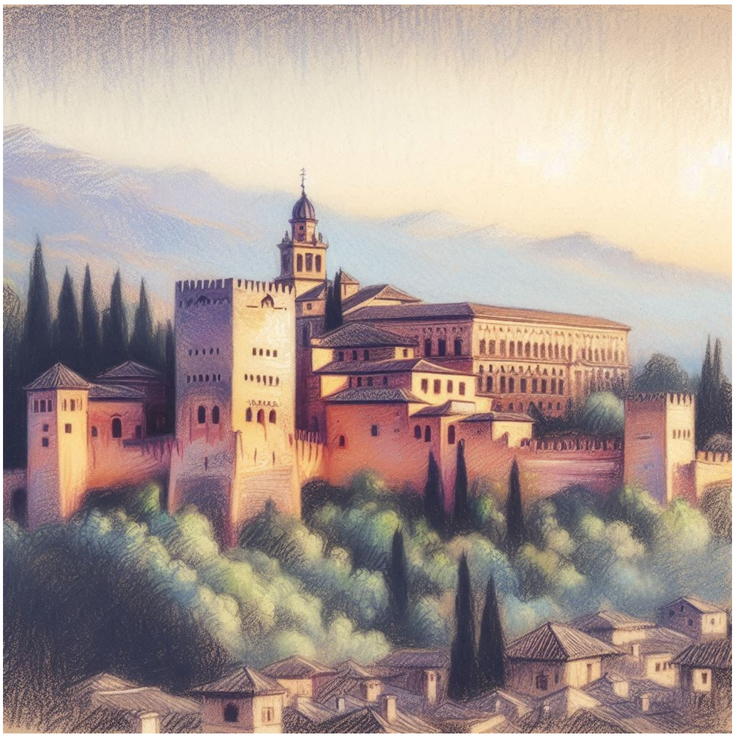

15-18 October 2025

Granada, Spain

Book your agenda for Granada 2025
See you soon in Southern Spain
